# Supplementary material for: Iodine-Catalyzed Cascade Annulation of 4-Hydroxycoumarins with Aurones: Access to Spirocyclic Benzofuran–Furocoumarins
Source: Molecules. 2024 Apr 9;29(8):1701. doi: 10.3390/molecules29081701 (PMC11052457; doi:10.3390/molecules29081701)
Supplement: Supplementary file 1 [file molecules-29-01701-s001.zip › molecules-2951171-supplementary.pdf]

# *Supporting information*

# Iodine-Catalyzed Cascade Annulation of 4-Hydroxycoumarins with Aurones: Access to Spirocyclic Benzofuran–Furocoumarins

Xuequan Wang, [1,*] Changhui Yang, [1] Dan Yue, [1] Mingde Xu, [1] Suyue Duan, [1,*] and Xianfu Shen [2,*]

[1]*School of Chemistry and Resources Engineering, Honghe University, Mengzi, Yunnan 661100, China*

[2]*College of Chemistry and Environmental Science, Qujing Normal University, Qujing, Yunnan 655011, China*

**Correspondence: huolixuanfeng@126.com (X.W.); 15287476872@163.com (S.D.); xianfu_shen@163.com (X.S.)*

***Table of Contents for Supporting Information***

1. General information........................................................................................................2

2. Optimization of reaction conditions......................................................................................2

3. General procedure...........................................................................................................2

4. Characterization data of products.......................................................................................3

5. $^{1}$H-NMR and $^{13}$C-NMR Spectral of products.............................................................18

## 1. General Information.

Unless otherwise specified, starting materials and reagents used in the reactions were commercially available and used without further purification. Aurones **2** were prepared by published procedures. $^{1}$H (400 MHz), $^{13}$C (100 MHz), DEPT 135 (100 MHz) and DEPT 90 (100 MHz) NMR spectra were recorded on a Bruker Avance 400 spectrometer in $CDCl_3$ or DMSO-$d_6$. HRMS were performed on AB QSTAR Pulsar mass spectrometer. Melting points were tested on XT-4A melting-point apparatus without corrected. The reactions were monitored by thin-layer chromatography (TLC) using silica gel GF254. For column chromatography, silica gel (200-300 mesh) was employed.

## 2. Optimization of reaction conditions.

**Table S1.** Optimization of reaction conditions [a].

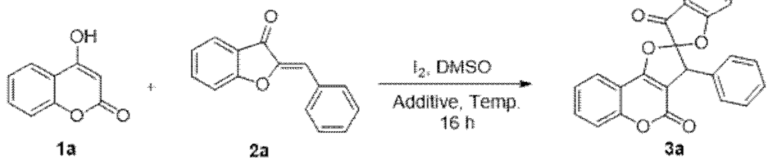


| Entry | $I_2$ (mol %) | 2a:1a | Additive (mol%) | Temp. | Yield (%) [b] | dr [c] |
|---|---|---|---|---|---|---|
| **1** | 20% | 1:1.2 | - | 80 | 54% | >20:1 |
| **2** | 20% | 1:1.2 | - | 100 | 58% | >20:1 |
| **3** | 20% | 1:1.2 | - | 120 | 52% | >20:1 |
| **4** | 30% | 1:1.2 | - | 100 | 57% | >20:1 |
| **5** | 20% | 1:1.2 | *L*-Proline（10%） | 100 | 56% | >20:1 |
| **6** | 20% | 1:1.2 | TBAI（10%） | 100 | 59% | >20:1 |
| **7** | 20% | 1:1.2 | TEBAC（10%） | 100 | 63% | >20:1 |
| **8** | 20% | 1:1.2 | TEBAC（20%） | 100 | 64% | >20:1 |
| **9** | 20% | 1:1.2 | TEBAC（40%） | 100 | 68% | >20:1 |
| **10** | 20% | 1:1.3 | TEBAC（40%） | 100 | 70% | >20:1 |
| **11** | 20% | 1:1.4 | TEBAC（40%） | 100 | 71% | >20:1 |
| **12** | 20% | 1:1.5 | TEBAC（40%） | 100 | 82% | >20:1 |

[a] Reaction conditions: aurone **2a** (0.25 mmol), 4-hydroxycoumarin **1a**, $I_2$ and additive in DMSO (0.5 mL) under air conditions for 16 h; [b] Isolated yields; [c] Based on final product $^{1}$H NMR spectra; TEBAC = benzyltriethylammonium chloride; TBAI = tetrabutylammonium iodide.

## 3. General procedure.

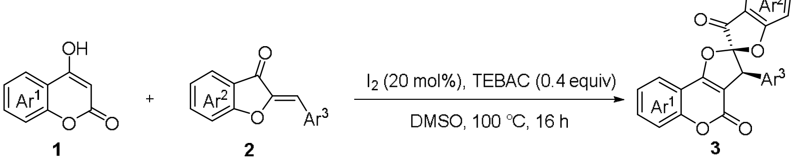


A mixture of 4-hydroxycoumarins **1** (0.375 mmol), aurones **2** (0.25 mmol), TEBAC (40 mol %) and $I_2$ (20 mol %) was stirred in DMSO (1 mL) at 100 °C for 16 h. Then saturated $Na_2S_2O_3$ solution (8 mL) was added to quench the reaction. And the product was extracted with $CH_2Cl_2$ (3 × 8 mL). The combined organic layers were dried over anhydrous $Na_2SO_4$, and

concentrated under reduced pressure. The crude product was subjected to flash column chromatography on silica gel (petroleum ether/ethyl acetate/$CH_2Cl_2$ = 10:1:5) to give the pure products **3aa-3bl** in 51-99% yields.

## 4. Characterization data of products.

**3'-phenyl-3*H*,3'*H*,4'*H*-spiro[benzofuran-2,2'-furo[3,2-*c*]chromene]-3,4'-dione (3aa)**

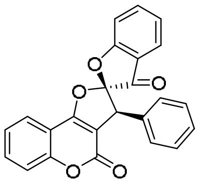

Yield 82%; White solid; Mp 243–244 °C; $^{1}$**H NMR** (400 MHz, DMSO-$d_6$): $\delta$ 7.85 (d, $J$ = 7.6 Hz, 1H), 7.81–7.75 (m, 3H), 7.59 (d, $J$ = 8.8 Hz, 1H), 7.47–7.43 (m, 1H), 7.31–7.28 (m, 4H), 7.25–7.21 (m, 2H), 7.06 (m, 1H), 5.14 (s, 1H); $^{13}$**C NMR** (100 MHz, DMSO-$d_6$): $\delta$ 194.13 (C), 170.13 (C), 164.76 (C), 157.99 (C), 155.19 (C), 141.21 (CH), 134.30 (CH), 132.61 (C), 129.45 (CH), 128.73 (CH), 128.47 (CH), 125.95 (CH), 125.27 (CH), 124.85 (CH), 123.31 (CH), 118.10, 117.39 (CH), 113.55 (CH), 111.38 (C), 111.14 (C), 104.50 (C), 50.88 (CH); **HRMS** (ESI-TOF): m/z calcd for $C_{24}H_{14}O_5Na$ $[M+Na]^+$: 405.0739, found: 405.0742.

**8'-methyl-3'-phenyl-3*H*,3'*H*,4'*H*-spiro[benzofuran-2,2'-furo[3,2-*c*]chromene]-3,4'-dione (3ab)**

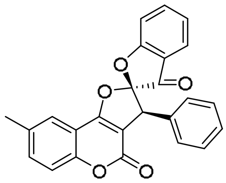

Yield 79%; White solid; Mp 263–264 °C; $^{1}$**H NMR** (400 MHz, $CDCl_3$): $\delta$ 7.66 (d, $J$ = 7.6 Hz, 1H), 7.50 (t, $J$ = 8.0 Hz, 1H), 7.42 (s, 1H), 7.36 (dd, $J$ = 8.4, 1.6 Hz, 1H), 7.26 (d, $J$ = 8.4 Hz, 1H), 7.18–7.17 (m, 3H), 7.09–7.06 (m, 3H), 6.74 (d, $J$ = 8.4 Hz, 1H), 5.02 (s, 1H), 2.33 (s, 3H); $^{13}$**C NMR** (100 MHz, $CDCl_3$): $\delta$ 194.36 (C), 170.46 (C), 165.38 (C), 158.76 (C), 153.56 (C), 139.84 (CH), 134.36 (CH), 134.22 (C), 131.49 (C), 128.88 (CH), 128.44 (CH), 128.21 (CH), 125.34 (CH), 123.68 (CH), 122.62 (CH), 118.46 (C), 116.86 (CH), 113.10 (CH), 111.37 (C), 110.94 (C), 103.77 (C), 52.06 (CH), 20.86 ($CH_3$); **HRMS** (ESI-TOF): m/z calcd for $C_{25}H_{16}O_5Na$ $[M+Na]^+$: 419.0895, found: 419.0896.

**7'-methyl-3'-phenyl-3*H*,3'*H*,4'*H*-spiro[benzofuran-2,2'-furo[3,2-*c*]chromene]-3,4'-dione (3ac)**

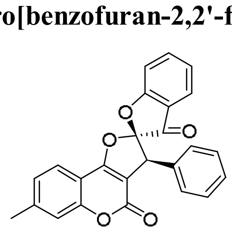

Yield 87%; Yellow solid; Mp 277–278 °C; $^{1}$**H NMR** (400 MHz, $CDCl_3$): $\delta$ 7.67 (dd, $J$ = 8.0, 0.8 Hz, 1H), 7.53–7.48 (m, 2H), 7.20–7.17 (m, 4H), 7.10–7.06 (m, 4H), 6.74 (d, $J$ = 8.4 Hz, 1H), 5.01 (s, 1H), 2.42 (s, 3H); $^{13}$**C NMR** (100 MHz, $CDCl_3$): $\delta$ 194.42 (C), 170.45 (C), 165.61 (C), 158.79 (C), 155.54 (C), 144.79 (C), 139.83 (CH), 131.56 (C), 128.86 (CH), 128.43 (CH), 128.19 (CH),

125.58 (CH), 125.34 (CH), 123.65 (CH), 122.66 (CH), 118.51 (C), 117.25 (CH), 113.09 (CH), 110.96 (C), 109.18 (C), 102.79 (C), 52.06 (CH), 22.12 ($CH_3$); **HRMS** (ESI-TOF): m/z calcd for $C_{25}H_{16}O_5Na$ $[M+Na]^+$: 419.0895, found: 419.0897.

**7'-methoxy-3'-phenyl-3*H*,3'*H*,4'*H*-spiro[benzofuran-2,2'-furo[3,2-*c*]chromene]-3,4'-dione (3ad)**

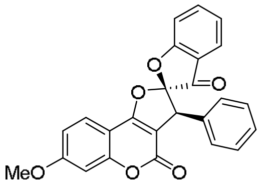


Yield 81%; Yellow solid; Mp 285–286 °C; $^1$**H NMR** (400 MHz, $CDCl_3$): $\delta$ 7.67 (d, $J$ = 8.0 Hz, 1H), 7.51 (t, $J$ = 8.4 Hz, 2H), 7.19–7.17 (m, 3H), 7.09–7.06 (m, 3H), 6.85 (d, $J$ = 1.6 Hz, 1H), 6.84–6.81 (m, 1H), 6.74 (d, $J$ = 8.4 Hz, 1H), 5.00 (s, 1H), 3.83 (s, 3H); $^{13}$**C NMR** (100 MHz, $CDCl_3$): $\delta$ 194.45 (C), 170.45 (C), 165.78 (C), 164.01 (C), 158.92 (C), 157.43 (C), 139.82 (CH), 131.68 (C), 128.86 (CH), 128.41 (CH), 128.15 (CH), 125.33 (CH), 124.02 (CH), 123.63 (CH), 118.53 (C), 113.08 (CH), 112.90 (CH), 110.97 (C), 104.90 (C), 100.90 (CH), 100.72 (C), 55.91 (CH), 51.96 (CH); **HRMS** (ESI-TOF): m/z calcd for $C_{25}H_{16}O_6Na$ $[M+Na]^+$: 435.0845, found: 435.0846.

**1'-phenyl-1'*H*,3*H*,11'*H*-spiro[benzofuran-2,2'-benzo[*h*]furo[3,2-*c*]chromene]-3,11'-dione (3ae)**

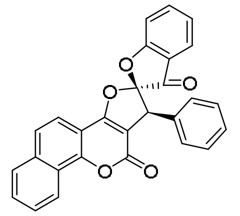

Yield 86%; Yellow solid; Mp 268–269 °C; $^1$**H NMR** (400 MHz, $CDCl_3$): $\delta$ 8.54 (dd, $J$ = 6.4, 3.2 Hz, 1H), 7.84–7.82 (m, 1H), 7.68 (d, $J$ = 7.6 Hz, 1H), 7.66–7.56 (m, 4H), 7.54–7.49 (m, 1H), 7.22–7.18 (m, 3H), 7.13–7.07 (m, 3H), 6.76 (d, $J$ = 8.0 Hz, 1H), 5.10 (s, 1H); $^{13}$**C NMR** (100 MHz, $CDCl_3$): $\delta$ 194.41 (C), 170.49 (C), 166.45 (C), 158.52 (C), 153.43 (C), 139.87 (CH), 135.51 (C), 131.48 (C), 129.35 (CH), 128.90 (CH), 128.48 (CH), 128.24 (CH), 128.10 (C), 127.52 (CH), 125.37 (CH), 124.55 (CH), 123.69 (CH), 123.10 (CH), 122.99 (C), 118.53 (C), 118.09 (CH), 113.11 (CH), 111.08 (C), 106.92 (C), 103.14 (C), 52.06 (CH); **HRMS** (ESI-TOF): m/z calcd for $C_{28}H_{16}O_5Na$ $[M+Na]^+$: 455.0895, found: 455.0893.

**8'-fluoro-3'-phenyl-3*H*,3'*H*,4'*H*-spiro[benzofuran-2,2'-furo[3,2-*c*]chromene]-3,4'-dione (3af)**

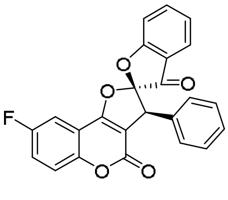


Yield 77%; White solid; Mp 245–246 °C; $^1$**H NMR** (400 MHz, $CDCl_3$): $\delta$ 7.67–7.65 (m, 1H),

7.53–7.49 (m, 1H), 7.37–7.34 (m, 1H), 7.31–7.25 (m, 2H), 7.20–7.17 (m, 3H), 7.10–7.06 (m, 3H), 6.75 (d, $J$ = 8.4 Hz, 1H), 5.03 (s, 1H); **$^{13}$C NMR** (100 MHz, $CDCl_3$): $\delta$ 194.05 (C), 170.43 (C), 164.57 (d, $J$ = 3.0 Hz, C), 158.54 (d, $J$ = 244.0 Hz, C), 158.11 (C), 151.50 (d, $J$ = 2.0 Hz, C), 139.96 (CH), 131.11 (C), 128.89 (CH), 128.52 (CH), 128.36 (CH), 125.42 (CH), 123.84 (CH), 120.92 (d, $J$ = 25.0 Hz, CH), 118.91 (d, $J$ = 9.0 Hz, CH), 118.33 (C), 113.12 (CH), 112.39 (d, $J$ = 10.0 Hz, C), 110.90 (C), 108.71 (d, $J$ = 26.0 Hz, CH), 104.91 (C), 52.01 (CH); **HRMS** (ESI-TOF): m/z calcd for $C_{24}H_{13}FO_5Na$ $[M+Na]^+$: 423.0645, found: 423.0644.

**8'-chloro-3'-phenyl-3*H*,3'*H*,4'*H*-spiro[benzofuran-2,2'-furo[3,2-*c*]chromene]-3,4'-dione (3ag)**

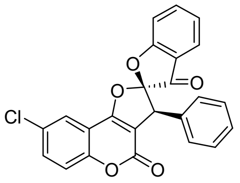


Yield 86%; White solid; Mp 278-279 °C; **$^{1}$H NMR** (400 MHz, $CDCl_3$): $\delta$ 7.68–7.66 (m, 1H), 7.61 (d, $J$ = 2.4 Hz, 1H), 7.54–7.49 (m, 2H), 7.32 (d, $J$ = 8.8 Hz, 1H), 7.21–7.18 (m, 3H), 7.11–7.06 (m, 3H), 6.76 (d, $J$ = 8.4 Hz, 1H), 5.03 (s, 1H); **$^{13}$C NMR** (100 MHz, $CDCl_3$): $\delta$ 194.01 (C), 170.43 (C), 164.23 (C), 157.93 (C), 153.64 (C), 139.97 (CH), 133.28 (CH), 131.06 (C), 129.88 (C), 128.89 (CH), 128.53 (CH), 128.38 (CH), 125.44 (CH), 123.86 (CH), 122.53 (CH), 118.61 (CH), 118.30 (C), 113.13 (CH), 112.75 (C), 110.90 (C), 104.92 (C), 51.93 (CH); **HRMS** (ESI-TOF): m/z calcd for $C_{24}H_{13}ClO_5Na$ $[M+Na]^+$: 439.0349, found: 439.0352.

**8'-bromo-3'-phenyl-3*H*,3'*H*,4'*H*-spiro[benzofuran-2,2'-furo[3,2-*c*]chromene]-3,4'-dione (3ah)**

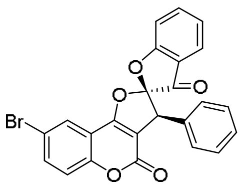


Yield 65%; Yellow solid; Mp 286–287 °C; **$^{1}$H NMR** (400 MHz, $CDCl_3$): $\delta$ 7.77 (d, $J$ = 2.4 Hz, 1H), 7.68–7.63 (m, 2H), 7.52 (t, $J$ = 7.6 Hz, 1H), 7.26 (d, $J$ = 8.8 Hz, 1H), 7.21–7.19 (m, 3H), 7.11–7.06 (m, 3H), 6.77 (d, $J$ = 8.4 Hz, 1H), 5.03 (s, 1H); **$^{13}$C NMR** (100 MHz, $CDCl_3$): $\delta$ 193.98 (C), 170.43 (C), 164.09 (C), 157.85 (C), 154.10 (C), 139.95 (CH), 136.06 (CH), 131.06 (C), 128.89 (CH), 128.53 (CH), 128.37 (CH), 125.55 (CH), 125.43 (CH), 123.85 (CH), 118.85 (CH), 118.31 (C), 117.08 (C), 113.22 (C), 113.12 (CH), 110.90 (C), 104.92 (C), 51.91 (CH); **HRMS** (ESI-TOF): m/z calcd for $C_{24}H_{13}BrO_5Na$ $[M+Na]^+$: 482.9844, found: 482.9848.

**5-methyl-3'-phenyl-3*H*,3'*H*,4'*H*-spiro[benzofuran-2,2'-furo[3,2-*c*]chromene]-3,4'-dione (3ai)**

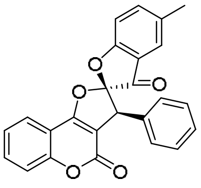


Yield 80%; White solid; Mp 289–290 °C; **$^{1}$H NMR** (400 MHz, $CDCl_3$): $\delta$ 7.62 (dd, $J$ = 7.6, 1.2 Hz, 1H), 7.58–7.54 (m, 1H), 7.44 (s, 1H), 7.37 (d, $J$ = 8.4 Hz, 1H), 7.32 (dd, $J$ = 8.4, 1.6 Hz, 1H), 7.25

(t, $J$ = 7.6 Hz, 1H), 7.21–7.17 (m, 3H), 7.08–7.06 (m, 2H), 6.65 (d, $J$ = 8.4 Hz, 1H), 5.01 (s, 1H), 2.28 (s, 3H); **$^{13}$C NMR** (100 MHz, $CDCl_3$): $\delta$ 194.40 (C), 168.94 (C), 165.43 (C), 158.58 (C), 155.33 (C), 141.05 (CH), 133.57 (C), 133.23 (CH), 131.50 (C), 128.87 (CH), 128.44 (CH), 128.19 (CH), 124.77 (CH), 124.30 (CH), 123.05 (CH), 118.33 (C), 117.12 (CH), 112.69 (CH), 111.73 (C), 111.24 (C), 103.91 (C), 52.01 (CH), 20.69 ($CH_3$); **HRMS** (ESI-TOF): m/z calcd for $C_{25}H_{16}O_5Na$ $[M+Na]^+$: 419.0895, found: 419.0900.

**5-methoxy-3'-phenyl-3*H*,3'*H*,4'*H*-spiro[benzofuran-2,2'-furo[3,2-*c*]chromene]-3,4'-dione (3aj)**

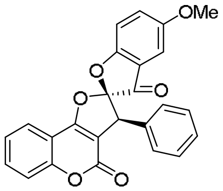


Yield 87%; White solid; Mp 269–270 °C; **$^1$H NMR** (400 MHz, $CDCl_3$): $\delta$ 7.63 (d, $J$ = 8.0 Hz, 1H), 7.58–7.54 (m, 1H), 7.37 (d, $J$ = 8.4 Hz, 1H), 7.26 (t, $J$ = 7.6 Hz, 1H), 7.20–7.18 (m, 3H), 7.13–7.04 (m, 4H), 6.67 (d, $J$ = 9.2 Hz, 1H), 5.01 (s, 1H), 3.73 (s, 3H); **$^{13}$C NMR** (100 MHz, $CDCl_3$): $\delta$ 194.64 (C), 165.79 (C), 165.43 (C), 158.56 (C), 156.04 (C), 155.32 (C), 133.25 (CH), 131.46 (C), 129.42 (CH), 128.85 (CH), 128.44 (CH), 128.23 (CH), 124.31 (CH), 123.04 (CH), 118.46 (C), 117.13 (CH), 114.03 (CH), 111.71 (C), 111.67 (C), 105.20 (CH), 103.93 (C), 56.01 ($CH_3$), 52.13 (CH); **HRMS** (ESI-TOF): m/z calcd for $C_{25}H_{16}O_6Na$ $[M+Na]^+$: 435.0845, found: 435.0848.

**7-methoxy-3'-phenyl-3*H*,3'*H*,4'H-spiro[benzofuran-2,2'-furo[3,2-*c*]chromene]-3,4'-dione (3ak)**

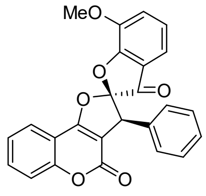


Yield 96% White solid; Mp 257–258 °C; **$^1$H NMR** (400 MHz, $CDCl_3$): $\delta$ 7.62 (dd, $J$ = 8.0, 1.6 Hz, 1H), 7.57–7.53 (m, 1H), 7.36 (d, $J$ = 8.4 Hz, 1H), 7.26–7.23 (m, 2H), 7.19–7.17 (m, 3H), 7.12–7.10 (m, 2H), 7.04–6.97 (m, 2H), 5.04 (s, 1H), 3.57 (s, 3H); **$^{13}$C NMR** (100 MHz, $CDCl_3$): $\delta$ 194.45 (C), 165.57 (C), 160.31 (C), 158.53 (C), 155.30 (C), 145.80 (C), 133.27 (CH), 131.23 (C), 128.93 (CH), 128.47 (CH), 128.24 (CH), 124.33 (CH), 124.17 (CH), 123.05 (CH), 122.70 (CH), 119.85 (C), 117.09 (CH), 116.60 (CH), 111.70 (C), 111.08 (C), 103.78 (C), 56.95 ($CH_3$), 52.53 (CH); **HRMS** (ESI-TOF): m/z calcd for $C_{25}H_{16}O_6Na$ $[M+Na]^+$: 435.0845, found: 435.0847.

**6-ethoxy-3'-phenyl-3*H*,3'*H*,4'*H*-spiro[benzofuran-2,2'-furo[3,2-*c*]chromene]-3,4'-dione (3al)**

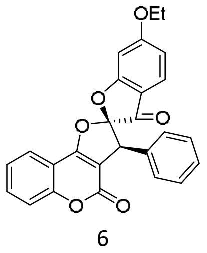

Yield 64%; White solid; Mp 246–247 °C; **$^1$H NMR** (400 MHz, $CDCl_3$): $\delta$ 7.76–7.72 (m, 1H), 7.69–7.63 (m, 2H), 7.50–7.45 (m, 1H), 7.39–7.28 (m, 4H), 7.21–7.17 (m, 2H), 6.72–6.67 (m, 1H), 6.24 (d, $J$ = 9.6 Hz, 1H), 5.14 (d, $J$ = 11.6 Hz, 1H), 4.07–4.00 (m, 2H), 1.45–1.39 (m, 3H); **$^{13}$C NMR** (100 MHz, $CDCl_3$): $\delta$ 191.43 (C), 173.07 (C), 169.19 (C), 165.49 (C), 158.62 (C), 155.35 (C), 133.21 (CH), 131.64 (C), 128.90 (CH), 128.45 (CH), 128.15 (CH), 126.57 (CH), 124.30 (CH), 123.07 (CH), 117.11 (CH), 113.32 (CH), 112.02 (C), 111.77 (C), 111.11 (C), 103.92 (C), 96.69 (CH), 64.78 ($CH_2$), 51.92 (CH), 14.40 ($CH_3$); **HRMS** (ESI-TOF): m/z calcd for $C_{26}H_{18}O_6Na$ $[M+Na]^+$: 449.1001, found: 449.1005.

**6-fluoro-3'-phenyl-3*H*,3'*H*,4'*H*-spiro[benzofuran-2,2'-furo[3,2-*c*]chromene]-3,4'-dione (3am)**

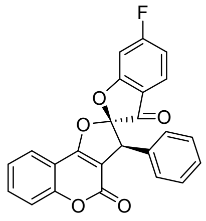

Yield 75%; White solid; Mp 224–225 °C; **$^1$H NMR** (400 MHz, $CDCl_3$): $\delta$ 7.62 (dd, $J$ = 7.6, 1.2 Hz, 1H), 7.59–7.54 (m, 1H), 7.37 (d, $J$ = 8.4 Hz, 1H), 7.32–7.30 (m, 1H), 7.28–7.26 (m, 1H), 7.25–7.23 (m, 1H), 7.21–7.17 (m, 3H), 7.08–7.05 (m, 2H), 6.73–6.70 (m, 1H), 5.02 (s, 1H); **$^{13}$C NMR** (100 MHz, $CDCl_3$): $\delta$ 193.99 (d, $J$ = 2.0 Hz, C), 166.57 (C), 165.30 (C), 158.51 (d, $J$ = 244.0 Hz, C), 158.42 (C), 155.34 (C), 133.37 (CH), 131.15 (C), 128.85 (CH), 128.52 (CH), 128.37 (CH), 127.37 (d, $J$ = 26.0 Hz, CH), 124.39 (CH), 122.99 (CH), 119.05 (d, $J$ = 8.0 Hz), 117.16 (CH), 114.39 (d, $J$ = 8.0 Hz, CH), 111.67 (d, $J$ = 14.0 Hz, C), 110.59 (d, $J$ = 24.0 Hz, CH), 103.85 (C), 52.32 (CH); **HRMS** (ESI-TOF): m/z calcd for $C_{24}H_{13}FO_5Na$ $[M+Na]^+$: 423.0645, found: 423.0646.

**6-chloro-3'-phenyl-3*H*,3'*H*,4'*H*-spiro[benzofuran-2,2'-furo[3,2-*c*]chromene]-3,4'-dione (3an)**

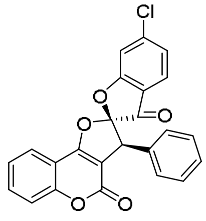

Yield 77%; White solid; Mp 265–266 °C; **$^1$H NMR** (400 MHz, $CDCl_3$): $\delta$ 7.62–7.55 (m, 3H), 7.38 (d, $J$ = 8.4 Hz, 1H), 7.26 (t, $J$ = 7.2 Hz, 1H), 7.22–7.19 (m, 3H), 7.08–7.05 (m, 3H), 6.78 (s, 1H), 5.03 (s, 1H); **$^{13}$C NMR** (100 MHz, $CDCl_3$): $\delta$ 192.75 (C), 170.55 (C), 165.25 (C), 158.40 (C), 155.36 (C), 146.25 (C), 133.37 (CH), 131.05 (C), 128.85 (CH), 128.58 (CH), 128.44 (CH), 126.08 (CH), 124.74 (CH), 124.39 (CH), 122.97 (CH), 117.17 (CH), 117.03 (C), 113.73 (CH), 111.58 (C), 111.43 (C), 103.85 (C), 52.22 (CH); **HRMS** (ESI-TOF): m/z calcd for $C_{24}H_{13}ClO_5Na$ $[M+Na]^+$: 439.0349, found: 439.0350.

**5-bromo-3'-phenyl-3*H*,3'*H*,4'*H*-spiro[benzofuran-2,2'-furo[3,2-*c*]chromene]-3,4'-dione (3ao)**

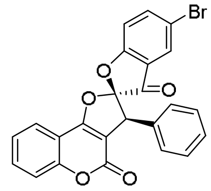

Yield 88%; White solid; Mp 250–251 °C; **$^{1}$H NMR** (400 MHz, $CDCl_3$): $\delta$ 7.77 (d, $J$ = 2.0 Hz, 1H), 7.62 (dd, $J$ = 8.0, 1.6 Hz, 1H), 7.59–7.55 (m, 2H), 7.37 (d, $J$ = 8.4 Hz, 1H), 7.26 (t, $J$ = 7.6 Hz, 1H), 7.20–7.18 (m, 3H), 7.07–7.05 (m, 2H), 6.66 (d, $J$ = 8.4 Hz, 1H), 5.02 (s, 1H); **$^{13}$C NMR** (100 MHz, $CDCl_3$): $\delta$ 193.04 (C), 169.13 (C), 165.27 (C), 158.38 (C), 155.35 (C), 142.32 (CH), 133.39 (CH), 131.03 (C), 128.84 (CH), 128.56 (CH), 128.41 (CH), 127.79 (CH), 124.40 (CH), 122.98 (CH), 120.15 (C), 117.17 (CH), 116.28 (C), 114.86 (CH), 111.57 (C), 111.29 (C), 103.81 (C), 52.35 (CH); **HRMS** (ESI-TOF): m/z calcd for $C_{24}H_{13}BrO_5Na$ $[M+Na]^+$: 482.9844, found: 482.9849.

**3'-(*p*-tolyl)-3*H*,3'*H*,4'*H*-spiro[benzofuran-2,2'-furo[3,2-*c*]chromene]-3,4'-dione (3ap)**

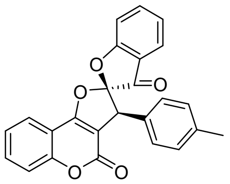

Yield 80%; White solid; Mp 232-233 °C; **$^{1}$H NMR** (400 MHz, $CDCl_3$): $\delta$ 7.76 (dd, $J$ = 7.6, 0.8 Hz, 1H), 7.72 (dd, $J$ = 8.0, 1.6 Hz, 1H), 7.67–7.59 (m, 2H), 7.47 (d, $J$ = 8.4 Hz, 1H), 7.37–7.33 (m, 1H), 7.18 (t, $J$ = 7.6 Hz, 1H), 7.11–7.06 (m, 4H), 6.88 (d, $J$ = 8.4 Hz, 1H), 5.10 (s, 1H), 2.31 (s, 3H); **$^{13}$C NMR** (100 MHz, $CDCl_3$): $\delta$ 194.46 (C), 170.53 (C), 165.23 (C), 158.58 (C), 155.30 (C), 139.84 (CH), 137.92 (C), 133.21 (CH), 129.23 (CH), 128.77 (CH), 128.27 (C), 125.35 (CH), 124.30 (CH), 123.68 (CH), 123.01 (C), 118.44 (C), 117.12 (CH), 113.20 (CH), 111.73 (C), 110.93 (C), 104.15 (C), 51.69 (CH), 21.25 ($CH_3$); **HRMS** (ESI-TOF): m/z calcd for $C_{25}H_{16}O_5Na$ $[M+Na]^+$: 419.0895, found: 419.0899.

**3'-(4-(*tert*-butyl)phenyl)-3*H*,3'*H*,4'*H*-spiro[benzofuran-2,2'-furo[3,2-*c*]chromene]-3,4'-dione (3aq)**

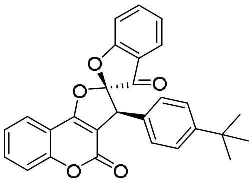

Yield 94%; Yellow oil; **$^{1}$H NMR** (400 MHz, $CDCl_3$): $\delta$ 7.66 (d, $J$ = 8.0 Hz, 1H), 7.62 (dd, $J$ = 7.6, 1.2 Hz, 1H), 7.57–7.48 (m, 2H), 7.36 (dd, $J$ = 8.4, 2.8 Hz, 1H), 7.26–7.22 (m, 1H), 7.20–7.18 (m, 2H), 7.10–7.05 (m, 1H), 6.99 (d, $J$ = 8.4 Hz, 2H), 6.75 (dd, $J$ = 8.4, 3.2 Hz, 1H), 5.00 (s, 1H), 1.18 (s, 9H); **$^{13}$C NMR** (100 MHz, $CDCl_3$): $\delta$ 194.48 (C), 170.55 (C), 165.23 (C), 158.61 (C), 155.29 (C), 150.93 (C), 139.75 (CH), 133.20 (CH), 128.46 (CH), 128.27 (C), 125.39 (CH), 125.34 (C), 124.30 (CH), 123.66 (CH), 123.02 (CH), 118.48 (C), 117.10 (CH), 113.16 (CH), 111.73 (C), 111.05 (C), 104.15 (C), 51.56 (CH), 34.54 (C), 31.29 ($CH_3$); **HRMS** (ESI-TOF): m/z calcd for

$C_{28}H_{22}O_5Na$ $[M+Na]^+$: 461.1365, found: 461.1367.

**3'-(2-methoxyphenyl)-3*H*,3'*H*,4'*H*-spiro[benzofuran-2,2'-furo[3,2-*c*]chromene]-3,4'-dione (3ar)**

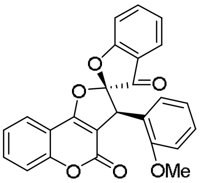

Yield 93%; Yellow solid; Mp 257–258 °C; **$^1$H NMR** (400 MHz, $CDCl_3$): $\delta$ 7.74 (d, $J$ = 7.6 Hz, 1H), 7.60 (d, $J$ = 8.0 Hz, 1H), 7.58–7.49 (m, 2H), 7.39 (d, $J$ = 8.4 Hz, 1H), 7.24 (t, $J$ = 7.6 Hz, 1H), 7.20–7.18 (m, 1H), 7.15 (d, $J$ = 4.8 Hz, 1H), 7.09 (d, $J$ = 7.6 Hz, 1H), 6.90 (t, $J$ = 7.2 Hz, 1H), 6.74 (d, $J$ = 8.4 Hz, 1H), 6.57 (d, $J$ = 8.0 Hz, 1H), 5.26 (s, 1H), 3.09 (s, 3H); **$^{13}$C NMR** (100 MHz, $CDCl_3$): $\delta$ 194.36 (C), 170.26 (C), 165.70 (C), 159.12 (C), 156.51 (C), 155.33 (C), 138.90 (CH), 133.21 (CH), 129.13 (CH), 128.63 (CH), 125.02 (CH), 124.29 (CH), 123.36 (CH), 123.03 (CH), 121.02 (C), 120.62 (CH), 118.67 (C), 117.06 (CH), 112.76 (CH), 111.75 (C), 111.27 (C), 109.25 (CH), 102.31 (C), 53.93 ($CH_3$), 46.66 (CH); **HRMS** (ESI-TOF): m/z calcd for $C_{25}H_{16}O_6Na$ $[M+Na]^+$: 435.0845, found: 435.0848.

**3'-(4-methoxyphenyl)-3*H*,3'*H*,4'*H*-spiro[benzofuran-2,2'-furo[3,2-*c*]chromene]-3,4'-dione (3as)**

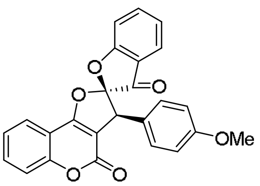

Yield 88%; Yellow solid; Mp 220–221 °C; **$^1$H NMR** (400 MHz, $CDCl_3$): $\delta$ 7.65 (dd, $J$ = 7.6, 0.8 Hz, 1H), 7.62 (dd, $J$ = 8.0, 1.2 Hz, 1H), 7.57–7.49 (m, 2H), 7.36 (d, $J$ = 8.4 Hz, 1H), 7.27–7.23 (m, 1H), 7.08 (t, $J$ = 7.2 Hz, 1H), 7.03–6.99 (m, 2H), 6.78 (d, $J$ = 8.4 Hz, 1H), 6.72 (d, $J$ = 8.8 Hz, 2H), 4.98 (s, 1H), 3.67 (s, 3H); **$^{13}$C NMR** (100 MHz, $CDCl_3$): $\delta$ 194.50 (C), 170.47 (C), 165.19 (C), 159.38 (C), 158.59 (C), 155.29 (C), 139.87 (CH), 133.23 (CH), 130.05 (CH), 125.32 (CH), 124.32 (CH), 123.68 (CH), 123.32 (C), 123.01 (CH), 118.47 (C), 117.11 (CH), 113.90 (CH), 113.19 (CH), 111.72 (C), 110.89 (C), 104.15 (C), 55.21 (CH), 51.47 (CH); **HRMS** (ESI-TOF): m/z calcd for $C_{25}H_{16}O_6Na$ $[M+Na]^+$: 435.0845, found: 435.0847.

**3'-(3,4,5-trimethoxyphenyl)-3*H*,3'*H*,4'*H*-spiro[benzofuran-2,2'-furo[3,2-*c*]chromene]-3,4'-dione (3at)**

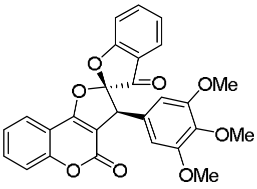

Yield 99%; White solid; Mp 279–280 °C; **$^1$H NMR** (400 MHz, $CDCl_3$): $\delta$ 7.68 (dd, $J$ = 7.6, 0.4 Hz, 1H), 7.65 (d, $J$ = 8.0 Hz, 1H), 7.61–7.53 (m, 2H), 7.40 (d, $J$ = 8.0 Hz, 1H), 7.30–7.26 (m, 1H),

7.13–7.09 (m, 1H), 6.79 (d, $J$ = 8.4 Hz, 1H), 6.24 (s, 2H), 4.99 (s, 1H), 3.72 (s, 3H), 3.63 (s, 6H); **$^{13}$C NMR** (100 MHz, $CDCl_3$): $\delta$ 194.40 (C), 170.61 (C), 165.66 (C), 158.62 (C), 155.35 (C), 153.10 (C), 140.04 (CH), 137.69 (C), 133.42 (CH), 126.80 (C), 125.26 (CH), 124.42 (CH), 123.74 (CH), 123.10 (CH), 118.52 (C), 117.16 (CH), 113.24 (CH), 111.68 (C), 110.87 (C), 105.85 (CH), 103.39 (C), 60.78 ($CH_3$), 56.10 ($CH_3$), 52.55 (CH); **HRMS** (ESI-TOF): m/z calcd for $C_{27}H_{20}O_8Na$ $[M+Na]^+$: 495.1056, found: 495.1060.

**3'-(3-nitrophenyl)-3*H*,3'*H*,4'*H*-spiro[benzofuran-2,2'-furo[3,2-*c*]chromene]-3,4'-dione (3av)**

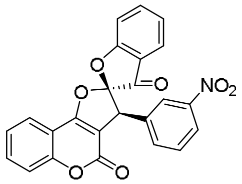


Yield 82%; White solid; Mp 239–240 °C; **$^1$H NMR** (400 MHz, $CDCl_3$): $\delta$ 8.17 (d, $J$ = 8.0 Hz, 1H), 8.09 (s, 1H), 7.77 (d, $J$ = 7.6 Hz, 1H), 7.74–7.67 (m, 2H), 7.63 (t, $J$ = 8.0 Hz, 1H), 7.54 (d, $J$ = 7.6 Hz, 1H), 7.49 (t, $J$ = 7.2 Hz, 2H), 7.38 (t, $J$ = 7.6 Hz, 1H), 7.21 (t, $J$ = 7.2 Hz, 1H), 6.86 (d, $J$ = 8.4 Hz, 1H), 5.20 (s, 1H); **$^{13}$C NMR** (100 MHz, $CDCl_3$): $\delta$ 193.44 (C), 170.13 (C), 165.93 (C), 158.39 (C), 155.45 (C), 148.26 (C), 140.23 (CH), 135.28 (CH), 133.94 (C), 133.79 (CH), 129.53 (CH), 125.68 (CH), 124.61 (CH), 124.23 (CH), 124.09 (CH), 123.52 (CH), 123.20 (CH), 118.19 (C), 117.30 (CH), 113.14 (CH), 111.44 (C), 110.34 (C), 103.01 (C), 51.25 (CH); **HRMS** (ESI-TOF): m/z calcd for $C_{24}H_{13}NO_7Na$ $[M+Na]^+$: 450.0590, found: 450.0588.

**3'-(3-fluorophenyl)-3H,3'H,4'H-spiro[benzofuran-2,2'-furo[3,2-c]chromene]-3,4'-dione (3aw)**

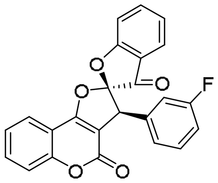


Yield 85%; White solid; Mp 206–207 °C; **$^1$H NMR** (400 MHz, $CDCl_3$): $\delta$ 7.75 (d, $J$ = 7.6 Hz, 1H), 7.70 (d, $J$ = 7.6 Hz, 1H), 7.68–7.60 (m, 2H), 7.46 (d, $J$ = 8.4 Hz, 1H), 7.34 (t, $J$ = 7.6 Hz, 1H), 7.24–7.17 (m, 2H), 6.99–6.93 (m, 2H), 6.92–6.89 (m, 1H), 6.87 (d, $J$ = 8.4 Hz, 1H), 5.09 (s, 1H); **$^{13}$C NMR** (100 MHz, $CDCl_3$): $\delta$ 193.98 (C), 170.41 (C), 165.61 (C), 162.71 (d, $J$ = 244.0 Hz), 158.45 (C), 155.37 (C), 140.05 (CH), 133.97 (d, $J$ = 7.0 Hz), 133.50 (CH), 129.99 (d, $J$ = 8.0 Hz, CH), 125.49 (CH), 124.71 (d, $J$ = 3.0 Hz, CH), 124.45 (CH), 123.93 (CH), 123.10 (CH), 118.30 (C), 117.19 (CH), 116.01 (d, $J$ = 52.0 Hz, CH), 115.41 (d, $J$ = 20.0 Hz, CH), 113.15 (CH), 111.55 (C), 110.64 (C), 103.45 (C), 51.51 (d, $J$ = 2.0 Hz, CH); **HRMS** (ESI-TOF): m/z calcd for $C_{24}H_{13}FO_5Na$ $[M+Na]^+$: 423.0645, found: 423.0644.

**3'-(4-fluorophenyl)-3H,3'H,4'H-spiro[benzofuran-2,2'-furo[3,2-c]chromene]-3,4'-dione (3ay)**

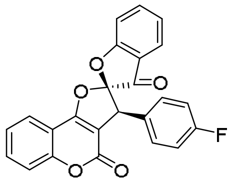

Yield 97%; White solid; Mp 206–207 °C; **$^1$H NMR** (400 MHz, $CDCl_3$): $\delta$ 7.75 (d, $J$ = 7.2 Hz, 1H), 7.71 (d, $J$ = 7.6 Hz, 1H), 7.67–7.60 (m, 2H), 7.46 (d, $J$ = 8.4 Hz, 1H), 7.34 (t, $J$ = 7.6 Hz, 1H), 7.20–7.13 (m, 3H), 6.96 (t, $J$ = 8.4 Hz, 2H), 6.86 (d, $J$ = 8.4 Hz, 1H), 5.09 (s, 1H); **$^{13}$C NMR** (100 MHz, $CDCl_3$): $\delta$ 194.13 (C), 170.34 (C), 165.46 (C), 162.57 (d, $J$ = 246 Hz, C), 158.53 (C), 155.34 (C), 140.04 (CH), 133.43 (CH), 130.60 (d, $J$ = 9.0 Hz, CH), 127.20 (d, $J$ = 4.0 Hz, C), 125.42 (CH), 124.43 (CH), 123.86 (CH), 123.07 (CH), 118.40 (C), 117.17 (CH), 115.52 (d, $J$ = 22.0 Hz, CH), 113.12 (CH), 111.61 (C), 110.68 (C), 103.69 (C), 51.36 (CH); **HRMS** (ESI-TOF): m/z calcd for $C_{24}H_{13}FO_5Na$ $[M+Na]^+$: 423.0645, found: 423.0647.

**methyl 4-(3,4'-dioxo-3*H*,3'*H*,4'*H*-spiro[benzofuran-2,2'-furo[3,2-*c*]chromen]-3'-yl)benzoate (3az)**

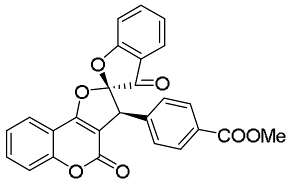


Yield 51%; White solid; Mp 228–229 °C; **$^1$H NMR** (400 MHz, $CDCl_3$): $\delta$ 7.87 (d, $J$ = 8.4 Hz, 2H), 7.68 (dd, $J$ = 8.0, 0.8 Hz, 1H), 7.63 (dd, $J$ = 7.6, 0.8 Hz, 1H), 7.60–7.56 (m, 1H), 7.54–7.50 (m, 1H), 7.38 (d, $J$ = 8.4 Hz, 1H), 7.29–7.25 (m, 1H), 7.17 (d, $J$ = 8.4 Hz, 2H), 7.10 (t, $J$ = 7.2 Hz, 1H), 6.74 (d, $J$ = 8.4 Hz, 1H), 5.08 (s, 1H), 3.80 (s, 3H); **$^{13}$C NMR** (100 MHz, $CDCl_3$): $\delta$ 193.87 (C), 170.29 (C), 166.74 (C), 165.69 (C), 158.45 (C), 155.39 (C), 140.08 (CH), 136.69 (C), 133.51 (CH), 130.06 (C), 129.76 (CH), 129.02 (CH), 125.46 (CH), 124.46 (CH), 123.93 (CH), 123.10 (CH), 118.30 (C), 117.20 (CH), 113.12 (CH), 111.56 (C), 110.67 (C), 103.37 (C), 52.20 ($CH_3$), 51.80 (CH); **HRMS** (ESI-TOF): m/z calcd for $C_{26}H_{16}O_7Na$ $[M+Na]^+$: 463.0794, found: 463.0792.

**4-(3,4'-dioxo-3*H*,3'*H*,4'*H*-spiro[benzofuran-2,2'-furo[3,2-*c*]chromen]-3'-yl)benzonitrile (3ba)**

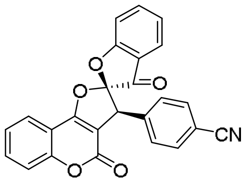


Yield 56%; Yellow solid; Mp 217-218 °C; **$^1$H NMR** (400 MHz, $CDCl_3$): $\delta$ 7.69 (d, $J$ = 7.6 Hz, 1H), 7.65–7.60 (m, 2H), 7.59–7.55 (m, 1H), 7.52–7.50 (m, 2H), 7.39 (d, $J$ = 8.4 Hz, 1H), 7.29 (t, $J$ = 7.6 Hz, 1H), 7.23 (d, $J$ = 8.0 Hz, 2H), 7.13 (t, $J$ = 7.6 Hz, 1H), 6.78 (d, $J$ = 8.4 Hz, 1H), 5.06 (s, 1H); **$^{13}$C NMR** (100 MHz, $CDCl_3$): $\delta$ 193.48 (C), 170.17 (C), 165.94 (C), 158.41 (C), 155.42 (C), 140.27 (CH), 137.09 (C), 133.76 (CH), 132.31 (CH), 129.79 (CH), 125.60 (CH), 124.60 (CH), 124.17 (CH), 123.16 (CH), 118.54 (C), 118.21 (C), 117.27 (CH), 113.11 (CH), 112.25 (C), 111.43 (C), 110.45 (C), 102.86 (C), 51.68 (CH); **HRMS** (ESI-TOF): m/z calcd for $C_{25}H_{13}NO_5Na$ $[M+Na]^+$: 430.0691, found: 430.0689.

**3'-(3-chlorophenyl)-3*H*,3'*H*,4'*H*-spiro[benzofuran-2,2'-furo[3,2-*c*]chromene]-3,4'-dione (3bb)**

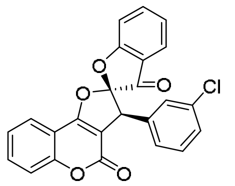


Yield 88%; Yellow solid; Mp 241–242 °C; **$^1$H NMR** (400 MHz, $CDCl_3$): $\delta$ 7.70-7.67 (m, 1H), 7.64–7.61 (m, 1H), 7.60–7.53 (m, 2H), 7.38 (dd, $J$ = 8.0, 3.6 Hz, 1H), 7.29–7.25 (m, 1H), 7.19–7.10 (m, 4H), 6.98 (d, $J$ = 7.2 Hz, 1H), 6.81 (dd, $J$ = 8.0, 3.2 Hz, 1H), 4.98 (s, 1H); **$^{13}$C NMR** (100 MHz, $CDCl_3$): $\delta$ 193.92 (C), 170.37 (C), 165.57 (C), 158.40 (C), 155.37 (C), 140.04 (CH), 134.37 (C), 133.59 (C), 133.50 (CH), 129.71 (CH), 129.05 (CH), 128.60 (CH), 127.25 (CH), 125.52 (CH), 124.44 (CH), 123.95 (CH), 123.10 (CH), 118.29 (C), 117.21 (CH), 113.20 (CH), 111.55 (C), 110.62 (C), 103.47 (C), 51.42 (CH); **HRMS** (ESI-TOF): m/z calcd for $C_{24}H_{13}ClO_5Na$ $[M+Na]^+$: 439.0349, found: 439.0350.

**3'-(3-bromophenyl)-3*H*,3'*H*,4'*H*-spiro[benzofuran-2,2'-furo[3,2-*c*]chromene]-3,4'-dione (3bc)**

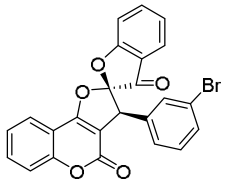


Yield 72%; Yellow solid; Mp 260–261 °C; **$^1$H NMR** (400 MHz, $CDCl_3$): $\delta$ 7.68 (dd, $J$ = 8.0, 1.2 Hz, 1H), 7.64–7.60 (m, 1H), 7.58–7.53 (m, 2H), 7.38 (d, $J$ = 8.4 Hz, 1H), 7.34–7.32 (m, 1H), 7.29–7.25 (m, 2H), 7.12 (t, $J$ = 7.6 Hz, 1H), 7.08-7.02 (m, 2H), 6.81 (d, $J$ = 8.4 Hz, 1H), 4.97 (s, 1H); **$^{13}$C NMR** (100 MHz, $CDCl_3$): $\delta$ 193.91 (C), 170.36 (C), 165.56 (C), 158.39 (C), 155.37 (C), 140.05 (CH), 133.85 (C), 133.50 (CH), 131.91 (CH), 131.51 (CH), 129.99 (CH), 127.72 (CH), 125.52 (CH), 124.44 (CH), 123.96 (CH), 123.10 (CH), 122.56 (C), 118.29 (C), 117.21 (C), 113.21 (CH), 111.55 (C), 110.63 (C), 103.47 (C), 51.37 (CH); **HRMS** (ESI-TOF): m/z calcd for $C_{24}H_{13}BrO_5Na$ $[M+Na]^+$: 482.9844, found: 482.9841.

**3'-(4-iodophenyl)-3*H*,3'*H*,4'*H*-spiro[benzofuran-2,2'-furo[3,2-*c*]chromene]-3,4'-dione (3bd)**

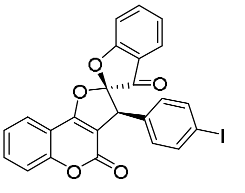


Yield 84%; White solid; Mp 255–256 °C; **$^1$H NMR** (400 MHz, $CDCl_3$): $\delta$ 7.67 (dd, $J$ = 7.6, 0.8 Hz, 1H), 7.62 (dd, $J$ = 7.6, 0.8 Hz, 1H), 7.58 (dd, $J$ = 7.6, 1.6 Hz, 1H), 7.55–7.51 (m, 3H), 7.38 (d, $J$ = 8.4 Hz, 1H), 7.26 (t, $J$ = 7.6 Hz, 1H), 7.11 (t, $J$ = 7.6 Hz, 1H), 6.85 (d, $J$ = 8.4 Hz, 2H), 6.81 (d, $J$ = 8.4 Hz, 1H), 4.96 (s, 1H); **$^{13}$C NMR** (100 MHz, $CDCl_3$): $\delta$ 193.97 (C), 170.36 (C), 165.58 (C), 158.46 (C), 155.35 (C), 140.09 (CH), 137.61 (CH), 133.47 (CH), 131.24 (C), 130.83 (CH), 125.45 (CH), 124.44 (CH), 123.93 (CH), 123.08 (CH), 118.30 (C), 117.19 (CH), 113.25 (CH), 111.57 (C), 110.56 (C), 103.44 (C), 94.22 (C), 51.50 (CH); **HRMS** (ESI-TOF): m/z calcd for $C_{24}H_{13}IO_5Na$ $[M+Na]^+$: 530.9705, found: 530.9706.

**3'-(2,4-dichlorophenyl)-3*H*,3'*H*,4'*H*-spiro[benzofuran-2,2'-furo[3,2-*c*]chromene]-3,4'-dione (3be)**

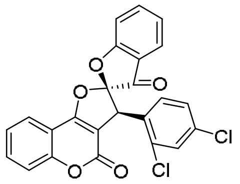

Yield 64%; Yellow solid; Mp 253–254 °C; $^{1}$**H NMR** (400 MHz, $CDCl_3$): $\delta$ 7.80 (d, $J$ = 7.6 Hz, 1H), 7.72–7.65 (m, 3H), 7.49 (d, $J$ = 8.4 Hz, 1H), 7.36 (t, $J$ = 7.6 Hz, 1H), 7.32–7.30 (m, 1H), 7.28 (d, $J$ = 2.4 Hz, 1H), 7.25 (t, $J$ = 5.6 Hz, 2H), 6.93 (d, $J$ = 8.4 Hz, 1H), 5.51 (s, 1H); $^{13}$**C NMR** (100 MHz, $CDCl_3$): $\delta$ 193.70 (C), 170.39 (C), 165.90 (C), 158.47 (C), 155.40 (C), 139.69 (CH), 135.06 (C), 134.70 (C), 133.65 (CH), 130.88 (CH), 129.18 (CH), 129.08 (C), 127.35 (CH), 125.62 (CH), 124.52 (CH), 124.08 (CH), 123.15 (CH), 118.15 (C), 117.22 (CH), 113.08 (CH), 111.46 (C), 110.40 (C), 102.28 (C), 47.76 (CH); **HRMS** (ESI-TOF): m/z calcd for $C_{24}H_{12}Cl_2O_5Na$ $[M+Na]^+$: 472.9959, found: 472.9958.

**3'-(4-(trifluoromethyl)phenyl)-3*H*,3'*H*,4'*H*-spiro[benzofuran-2,2'-furo[3,2-*c*]chromene]-3,4'-dione (3bf)**

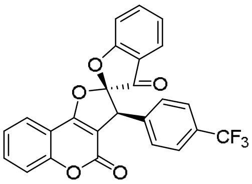

Yield 90%; Yellow solid; Mp 220–221 °C; $^{1}$**H NMR** (400 MHz, $CDCl_3$): $\delta$ 7.69 (dd, $J$ = 7.6, 0.8 Hz, 1H), 7.64 (dd, $J$ = 8.0, 1.6 Hz, 1H), 7.61–7.59 (m, 1H), 7.57–7.53 (m, 1H), 7.47 (d, $J$ = 8.0 Hz, 2H), 7.39 (d, $J$ = 8.4 Hz, 1H), 7.30–7.26 (m, 1H), 7.22 (d, $J$ = 8.0 Hz, 2H), 7.12 (t, $J$ = 7.6 Hz, 1H), 6.78 (d, $J$ = 8.0 Hz, 1H), 5.08 (s, 1H); $^{13}$**C NMR** (100 MHz, $CDCl_3$): $\delta$ 193.76 (C), 170.30 (C), 165.74 (C), 158.45 (C), 155.41 (C), 140.13 (CH), 135.65 (CH), 133.59 (C), 130.42 (q, $J$ = 32.0 Hz, $CF_3$), 129.35 (CH), 125.50 (q, $J$ = 4.0 Hz, CH), 124.50 (CH), 124.02 (CH), 123.12 (CH), 118.26 (C), 117.23 (CH), 113.17 (CH), 111.53 (CH), 110.60 (CH), 103.25 (C), 51.52 (CH); **HRMS** (ESI-TOF): m/z calcd for $C_{25}H_{13}F_3O_5Na$ $[M+Na]^+$: 473.0613, found: 473.0616.

**3'-([1,1'-biphenyl]-4-yl)-3*H*,3'*H*,4'*H*-spiro[benzofuran-2,2'-furo[3,2-*c*]chromene]-3,4'-dione (3bg)**

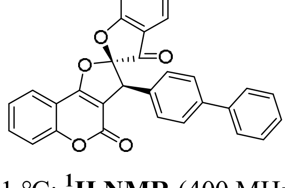

Yield 88%; White solid; Mp 280–281 °C; $^{1}$**H NMR** (400 MHz, $CDCl_3$): $\delta$ 7.68 (dd, $J$ = 8.0, 0.8 Hz, 1H), 7.63 (dd, $J$ = 7.6, 1.2 Hz, 1H), 7.58–7.54 (m, 1H), 7.52–7.50 (m, 1H), 7.49–7.47 (m, 1H), 7.46 (s, 1H), 7.43–7.41 (m, 2H), 7.38 (d, $J$ = 8.0 Hz, 1H), 7.34–7.30 (m, 2H), 7.28–7.22 (m, 2H), 7.15 (d, $J$ = 8.4 Hz, 2H), 7.10–7.06 (m, 1H), 6.77 (d, $J$ = 8.4 Hz, 1H), 5.07 (s, 1H); $^{13}$**C NMR** (100 MHz, $CDCl_3$): $\delta$ 194.32 (C), 170.50 (C), 165.44 (C), 158.61 (C), 155.36 (C), 140.97 (C), 140.55

(C), 139.92 (CH), 133.33 (CH), 130.47 (C), 129.31 (CH), 128.76 (CH), 127.40 (CH), 127.19 (CH), 127.06 (CH), 125.40 (CH), 124.38 (CH), 123.77 (CH), 123.07 (CH), 118.45 (C), 117.17 (CH), 113.23 (CH), 111.71 (C), 110.96 (C), 103.93 (C), 51.73 (CH); **HRMS** (ESI-TOF): m/z calcd for $C_{30}H_{18}O_5Na$ $[M+Na]^+$: 481.1052, found: 481.1053.

**3'-(naphthalen-2-yl)-3*H*,3'*H*,4'*H*-spiro[benzofuran-2,2'-furo[3,2-*c*]chromene]-3,4'-dione (3bh)**

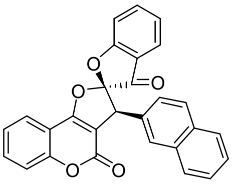

Yield 89%; White solid; Mp 257–258 °C; **$^1$H NMR** (400 MHz, $CDCl_3$): $\delta$ 7.82–7.74 (m, 5H), 7.69 (s, 1H), 7.68–7.65 (m, 1H), 7.56–7.52 (m, 1H), 7.50–7.45 (m, 3H), 7.39–7.35 (m, 1H), 7.32 (dd, $J$ = 8.4, 1.6 Hz, 1H), 7.15 (t, $J$ = 7.6 Hz, 1H), 6.79 (d, $J$ = 8.4 Hz, 1H), 5.32 (s, 1H); **$^{13}$C NMR** (100 MHz, $CDCl_3$): $\delta$ 194.36 (C), 170.52 (C), 165.43 (C), 158.60 (C), 155.39 (C), 139.89 (CH), 133.35 (CH), 133.19 (C), 129.12 (C), 128.34 (CH), 128.21 (CH), 128.04 (CH), 127.67 (CH), 126.51 (CH), 126.21 (CH), 126.15 (CH), 125.40 (CH), 124.39 (CH), 123.77 (CH), 123.09 (CH), 118.31 (C), 117.18 (CH), 113.23 (CH), 111.73 (C), 111.03 (C), 104.07 (C), 52.05 (CH); **HRMS** (ESI-TOF): m/z calcd for $C_{28}H_{16}O_5Na$ $[M+Na]^+$: 455.0895, found: 455.0895.

**3'-(furan-2-yl)-3*H*,3'*H*,4'*H*-spiro[benzofuran-2,2'-furo[3,2-*c*]chromene]-3,4'-dione (3bi)**

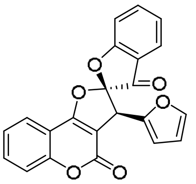

Yield 69%; Yellow solid; Mp 194–195 °C; **$^1$H NMR** (400 MHz, $CDCl_3$): $\delta$ 7.70 (d, $J$ = 7.6 Hz, 1H), 7.62–7.54 (m, 3H), 7.37 (d, $J$ = 8.4 Hz, 1H), 7.27–7.23 (m, 2H), 7.14 (t, $J$ = 7.6 Hz, 1H), 6.91 (d, $J$ = 8.4 Hz, 1H), 6.26–6.23 (m, 2H), 5.11 (s, 1H); **$^{13}$C NMR** (100 MHz, $CDCl_3$): $\delta$ 193.87 (C), 170.74 (C), 165.33 (C), 158.37 (C), 155.27 (C), 145.52 (C), 143.07 (CH), 139.94 (CH), 133.46 (CH), 125.55 (CH), 124.39 (CH), 123.92 (CH), 123.09 (CH), 118.12 (C), 117.14 (CH), 113.21 (CH), 111.56 (C), 110.61 (CH), 110.34 (C), 110.04 (CH), 101.82 (C), 45.50 (CH); **HRMS** (ESI-TOF): m/z calcd for $C_{22}H_{12}O_6Na$ $[M+Na]^+$: 395.0532, found: 395.0537.

**3'-(thiophen-2-yl)-3*H*,3'*H*,4'*H*-spiro[benzofuran-2,2'-furo[3,2-*c*]chromene]-3,4'-dione (3bj)**

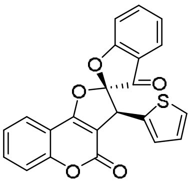

Yield 84%; Yellow solid; Mp 194–195 °C; **$^1$H NMR** (400 MHz, $CDCl_3$): $\delta$ 7.68 (dd, $J$ = 7.6, 0.8 Hz, 1H), 7.62 (dd, $J$ = 8.0, 1.6 Hz, 1H), 7.60–7.55 (m, 2H), 7.37 (d, $J$ = 8.4 Hz, 1H), 7.28–7.24 (m, 1H), 7.20–7.18 (m, 1H), 7.14–7.10 (m, 1H), 6.89 (d, $J$ = 8.4 Hz, 1H), 6.86–6.84 (m, 2H), 5.33 (s,

1H); **$^{13}$C NMR** (100 MHz, $CDCl_3$): $\delta$ 193.98 (C), 170.67 (C), 165.10 (C), 158.29 (C), 155.31 (C), 139.99 (CH), 133.68 (C), 133.47 (CH), 128.09 (CH), 126.72 (CH), 126.40 (CH), 125.46 (CH), 124.38 (CH), 123.87 (CH), 123.14 (CH), 118.39 (C), 117.17 (CH), 113.24 (CH), 111.57 (C), 110.14 (C), 103.89 (C), 47.15 (CH); **HRMS** (ESI-TOF): m/z calcd for $C_{22}H_{12}O_5SNa$ $[M+Na]^+$: 411.0303, found: 411.0305.

**5-nitro-3'-phenyl-3*H*,3'*H*,4'*H*-spiro[benzofuran-2,2'-furo[3,2-*c*]chromene]-3,4'-dione (3bk)**

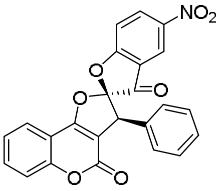


Yield 62%; White solid; Mp 265–266 °C; **$^1$H NMR** (400 MHz, $CDCl_3$): $\delta$ 8.57 (s, 1H), 8.41 (d, *J* = 9.2 Hz, 1H), 7.64-7.59 (m, 2H), 7.41 (d, *J* = 8.4 Hz, 1H), 7.30 (t, *J* = 7.6 Hz, 1H), 7.21-7.19 (m, 3H), 7.08 (s, 2H), 6.90 (d, *J* = 9.2 Hz, 1H), 5.10 (s, 1H); **$^{13}$C NMR** (100 MHz, $CDCl_3$): $\delta$ 192.51 (C), 172.82 (C), 165.17 (C), 158.19 (C), 155.41 (C), 143.90 (C), 134.57 (CH), 133.62 (CH), 130.41 (C), 128.79 (CH), 128.72 (CH), 124.54 (CH), 122.90 (CH), 121.84 (CH), 119.01 (C), 117.27 (CH), 113.93 (CH), 112.15 (C), 111.38 (C), 103.66 (C), 52.86 (CH); **HRMS** (ESI-TOF): m/z calcd for $C_{24}H_{13}NO_7Na$ $[M+Na]^+$: 450.0590, found: 450.0596.

**8'-nitro-3'-phenyl-3*H*,3'*H*,4'*H*-spiro[benzofuran-2,2'-furo[3,2-*c*]chromene]-3,4'-dione (3bl)**

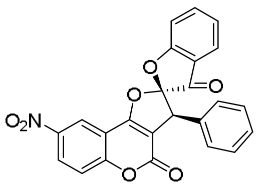


Yield 81%; Yellow solid; Mp 315–317 °C; **$^1$H NMR** (400 MHz, $CDCl_3$): $\delta$ 8.56 (d, *J* = 2.4 Hz, 1H), 8.43 (dd, *J* = 9.2, 2.4 Hz, 1H), 7.70 (d, *J* = 7.6 Hz, 1H), 7.56 (t, *J* = 8.0 Hz, 1H), 7.51 (d, *J* = 9.2 Hz, 1H), 7.41-7.37 (m, 1H), 7.24-7.22 (m, 2H), 7.15-7.10 (m, 3H), 6.81 (d, *J* = 8.0 Hz, 1H), 5.06 (s, 1H); **$^{13}$C NMR** (100 MHz, $CDCl_3$): $\delta$ 193.65 (C), 170.44 (C), 164.07 (C), 158.40 (C), 156.86 (C), 143.77 (C), 140.12 (CH), 130.62 (C), 128.89 (CH), 128.64 (CH), 128.58 (CH), 127.80 (CH), 125.56 (CH), 124.07 (CH), 119.51 (CH), 118.35 (CH), 118.15 (C), 113.17 (CH), 112.06 (C), 110.92 (C), 105.86 (C), 51.78 (CH); **HRMS** (ESI-TOF): m/z calcd for $C_{24}H_{13}NO_7Na$ $[M+Na]^+$: 450.0590, found: 450.0592.

**4-hydroxy-3-((4-nitrophenyl)(3-oxobenzofuran-2(3*H*)-ylidene)methyl)-2*H*-chromen-2-one (4au)**

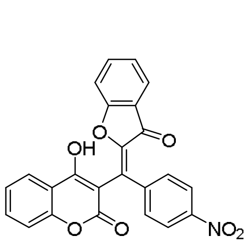


Yield 78%; Yellow solid; Mp 238–239 °C; **$^1$H NMR** (400 MHz, $CDCl_3$): $\delta$ 11.46 (s, 1H), 8.21 (d, *J* = 8.8 Hz, 2H), 7.96 (dd, *J* = 7.6, 1.2 Hz, 1H), 7.84 (dd, *J* = 8.0, 1.6 Hz, 1H), 7.67 (d, *J* = 8.8 Hz,

2H), 7.64–7.59 (m, 1H), 7.48–7.44 (m, 2H), 7.40 (t, $J$ = 8.0 Hz, 1H), 6.98 (d, $J$ = 8.4 Hz, 1H), 6.83–6.79 (m, 1H); **$^{13}$C NMR** (100 MHz, $CDCl_3$): $\delta$ 186.27 (C), 163.66 (C), 158.81 (C), 156.53 (C), 153.71 (C), 148.10 (C), 147.67 (C), 137.47 (CH), 135.04 (C), 133.12 (CH), 131.76 (CH), 131.38 (CH), 131.03 (C), 125.26 (CH), 123.33 (CH), 121.86 (CH), 119.33 (CH), 118.83 (CH), 118.56 (C), 117.71 (CH), 111.66 (C), 110.01 (C); **HRMS** (ESI-TOF): m/z calcd for $C_{24}H_{13}NO_7Na$ $[M+Na]^+$: 450.0590, found: 450.0589.

**3-((2-fluorophenyl)(3-oxobenzofuran-2(3*H*)-ylidene)methyl)-4-hydroxy-2*H*-chromen-2-one (4ax)**

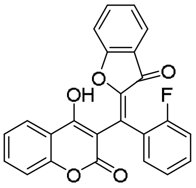


Yield 77%; White solid; Mp 196–197 °C; **$^1$H NMR** (400 MHz, $CDCl_3$): $\delta$ 7.80 (dd, $J$ = 7.6, 0.8 Hz, 1H), 7.72–7.69 (m, 1H), 7.67–7.62 (m, 2H), 7.49 (d, $J$ = 8.4 Hz, 1H), 7.38–7.34 (m, 1H), 7.32–7.26 (m, 2H), 7.24–7.20 (m, 1H), 7.16 (t, $J$ = 7.2 Hz, 1H), 6.95 (t, $J$ = 9.6 Hz, 1H), 6.89 (d, $J$ = 8.4 Hz, 1H), 5.38 (s, 1H); **$^{13}$C NMR** (100 MHz, $CDCl_3$): $\delta$ 193.80 (C), 170.39 (C), 158.67 (C), 155.34 (C), 139.67 (CH), 133.43 (CH), 130.01 (d, $J$ = 8.0 Hz, CH), 125.58 (CH), 124.42 (CH), 124.25 (d, $J$ = 3.0 Hz, CH), 123.90 (CH), 123.09 (CH), 119.38 (d, $J$ = 14.0 Hz, (C)), 118.16 (C), 117.15 (CH), 115.09 (d, $J$ = 22.0 Hz, CH), 113.00 (CH), 111.61 (C), 110.59 (C); **HRMS** (ESI-TOF): m/z calcd for $C_{24}H_{13}FO_5Na$ $[M+Na]^+$: 423.0645, found: 423.0644.

**methyl-4-((4-hydroxy-2-oxo-2*H*-chromen-3-yl)(3-oxobenzofuran-2(3*H*)-ylidene)methyl)benzoate (4az)**

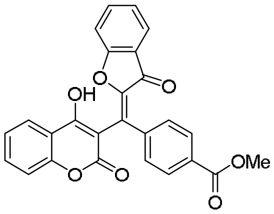


Yield 16%; Yellow solid; Mp 214–215 °C; **$^1$H NMR** (400 MHz, $CDCl_3$): $\delta$ 11.51 (s, 1H), 8.02–8.00 (m, 2H), 7.96 (dd, $J$ = 7.6, 1.2 Hz, 1H), 7.73 (dd, $J$ = 8.0, 1.6 Hz, 1H), 7.62–7.58 (m, 1H), 7.56–7.53 (m, 2H), 7.45–7.41 (m, 2H), 7.40–7.36 (m, 1H), 6.96 (dd, $J$ = 8.4, 0.8 Hz, 1H), 6.75–6.71 (m, 1H), 3.86 (s, 3H); **$^{13}$C NMR** (100 MHz, $CDCl_3$): $\delta$ 186.79 (C), 166.60 (C), 163.44 (C), 158.75 (C), 156.62 (C), 153.67 (C), 147.32 (C), 137.22 (CH), 132.87 (CH), 132.84 (C), 132.07 (C), 131.95 (CH), 130.68 (C), 130.36 (CH), 129.42 (CH), 125.08 (CH), 121.86 (CH), 119.16 (CH), 118.61 (CH), 117.62 (CH), 111.82 (C), 110.09 (C), 52.32 ($CH_3$), 29.74 (C); **HRMS** (ESI-TOF): m/z calcd for $C_{26}H_{16}O_7Na$ $[M+Na]^+$: 463.0794, found: 463.0791.

**4-((4-hydroxy-2-oxo-2*H*-chromen-3-yl)(3-oxobenzofuran-2(3*H*)-ylidene)methyl)benzonitrile (4ba)**

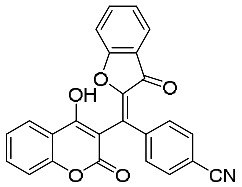

Yield 26%; Yellow solid; Mp 259–260 °C; **$^1$H NMR** (400 MHz, $CDCl_3$): $\delta$ 11.46 (s, 1H), 7.96 (d, $J$ = 7.6 Hz, 1H), 7.78 (dd, $J$ = 8.0, 0.8 Hz, 1H), 7.65–7.59 (m, 5H), 7.48–7.44 (m, 2H), 7.39 (t, $J$ = 8.0 Hz, 1H), 6.99 (d, $J$ = 8.8 Hz, 1H), 6.80–6.77 (m, 1H); **$^{13}$C NMR** (100 MHz, $CDCl_3$): $\delta$ 186.41 (C), 163.59 (C), 158.82 (C), 156.55 (C), 153.70 (C), 147.53 (C), 137.41 (CH), 133.08 (C), 133.05 (CH), 131.86 (CH), 131.78 (CH), 131.28 (C), 131.08 (CH), 125.21 (CH), 121.85 (CH), 119.26 (CH), 118.78 (CH), 118.55 (C), 118.50 (C), 117.69 (CH), 113.01 (C), 111.69 (C), 109.93 (C); **HRMS** (ESI-TOF): m/z calcd for $C_{25}H_{13}NO_5Na$ $[M+Na]^+$: 430.0691, found: 430.0691.

# 5. $^{1}H$ NMR and $^{13}C$ NMR spectral of products.

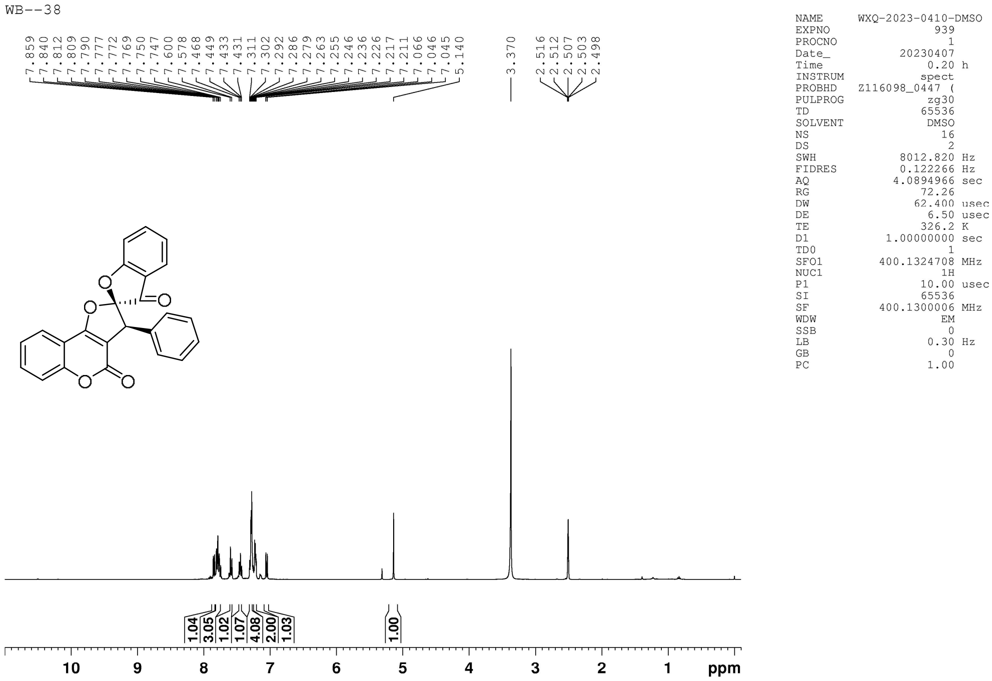


**Figure S1.** $^{1}H$ NMR (400 MHz, DMSO-$d_6$) spectra of compound **3aa**

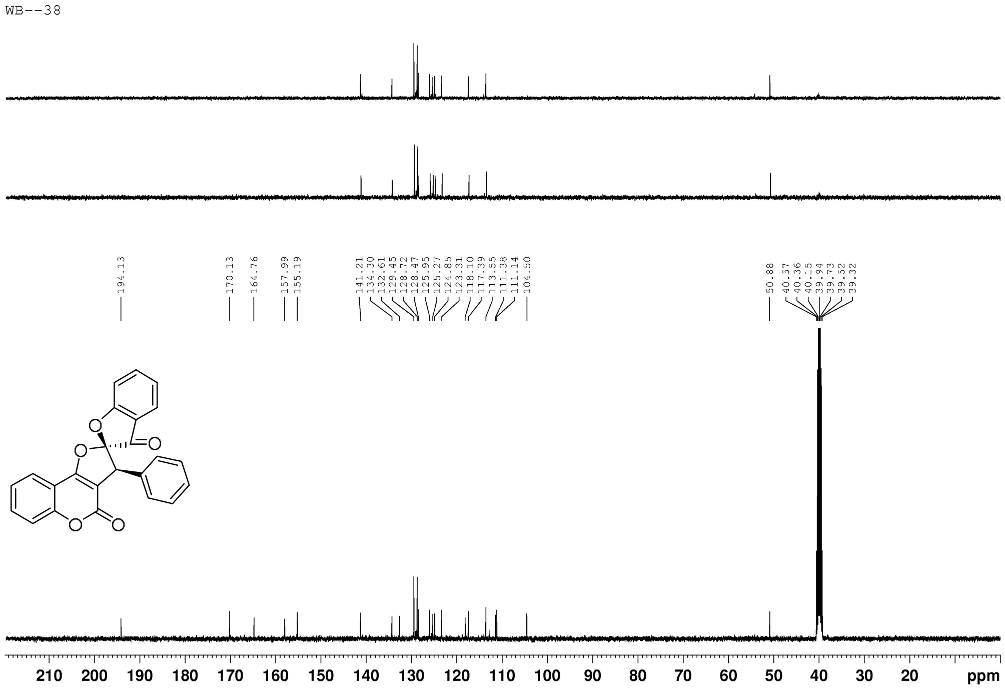


**Figure S2.** $^{13}C$ NMR (100 MHz, DMSO-$d_6$) spectra of compound **3aa**

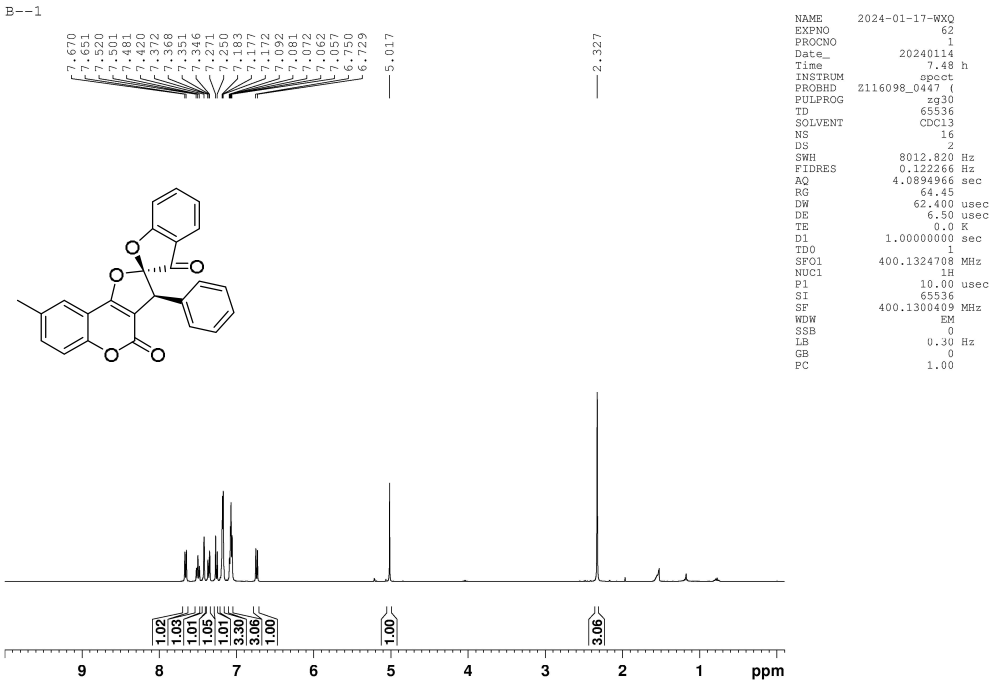

**Figure S3.** $^{1}H$ NMR (400 MHz, $CDCl_3$) spectra of compound **3ab**

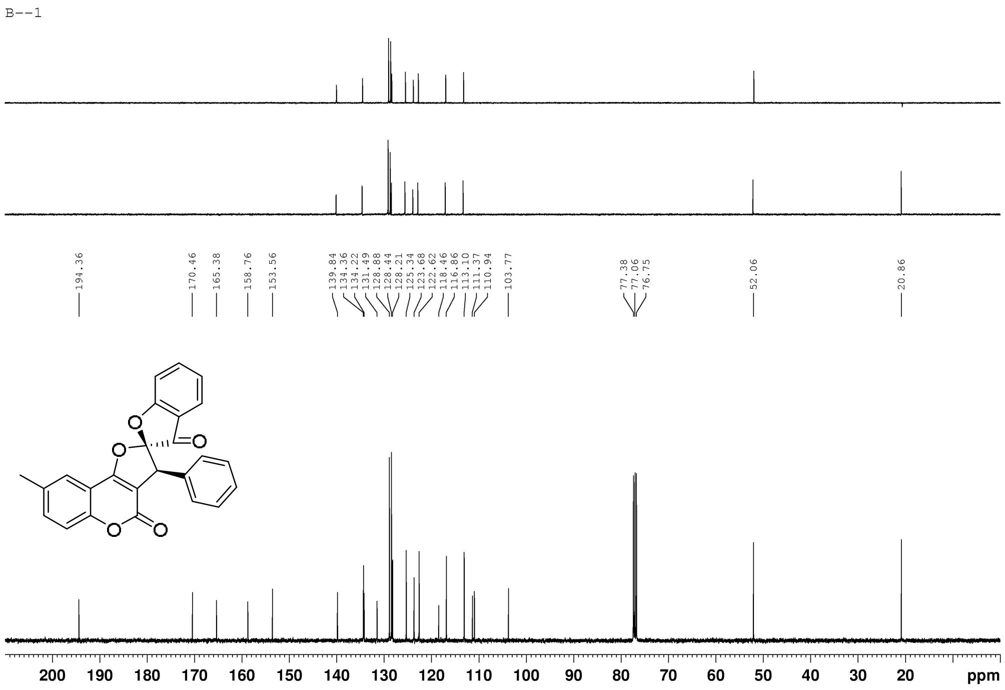

**Figure S4.** $^{13}C$ NMR (100 MHz, $CDCl_3$) spectra of compound **3ab**

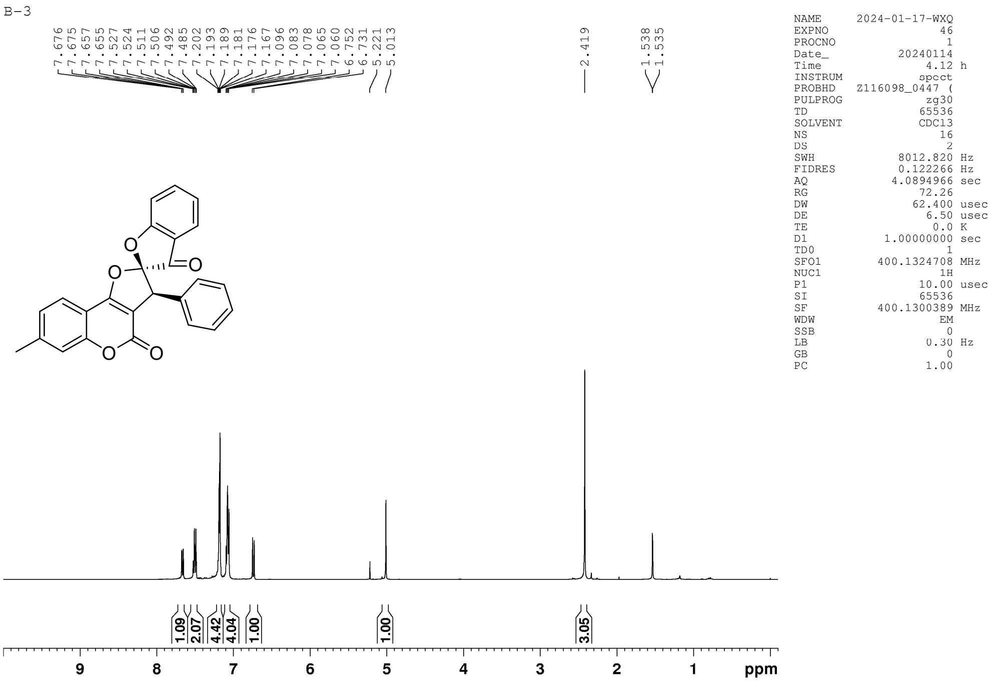


**Figure S5.** $^{1}$H NMR (400 MHz, $CDCl_3$) spectra of compound **3ac**

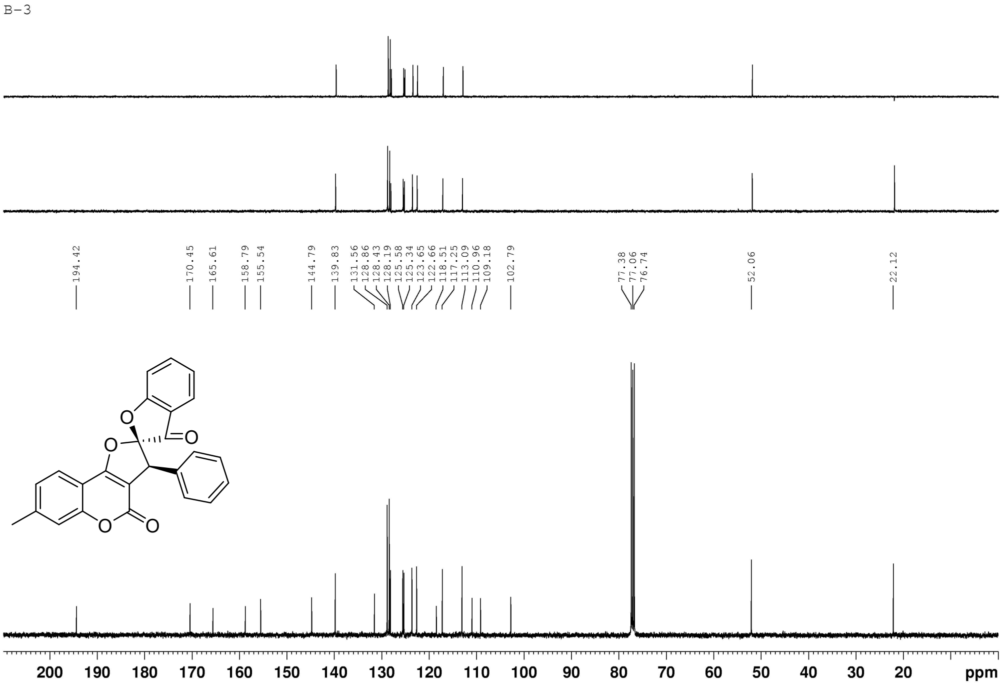


**Figure S6.** $^{13}$C NMR (100 MHz, $CDCl_3$) spectra of compound **3ac**

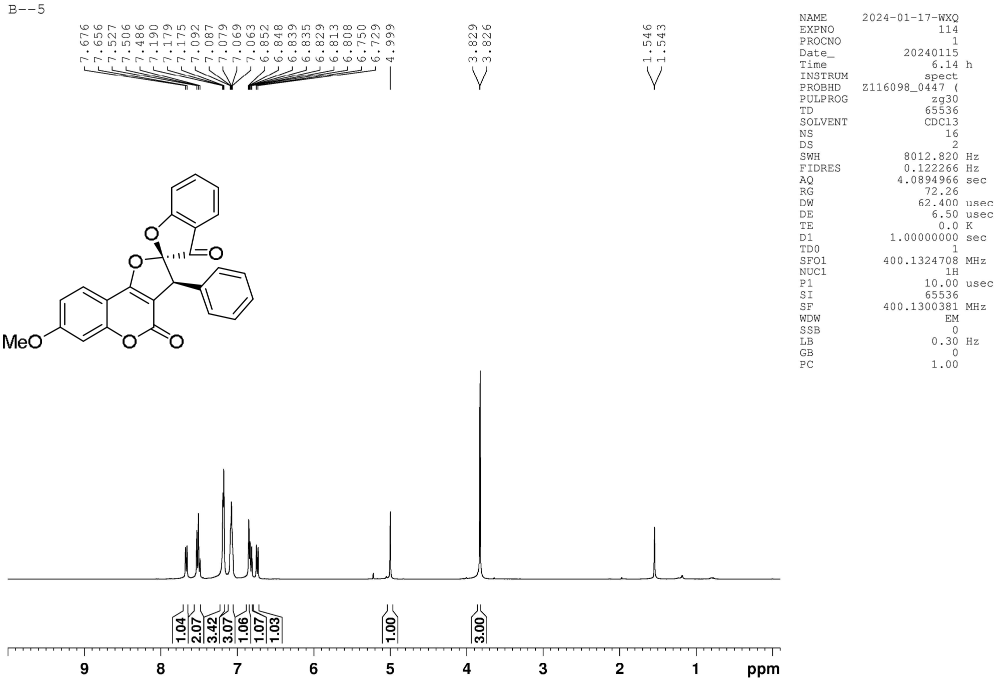


**Figure S7.** $^{1}H$ NMR (400 MHz, $CDCl_3$) spectra of compound **3ad**

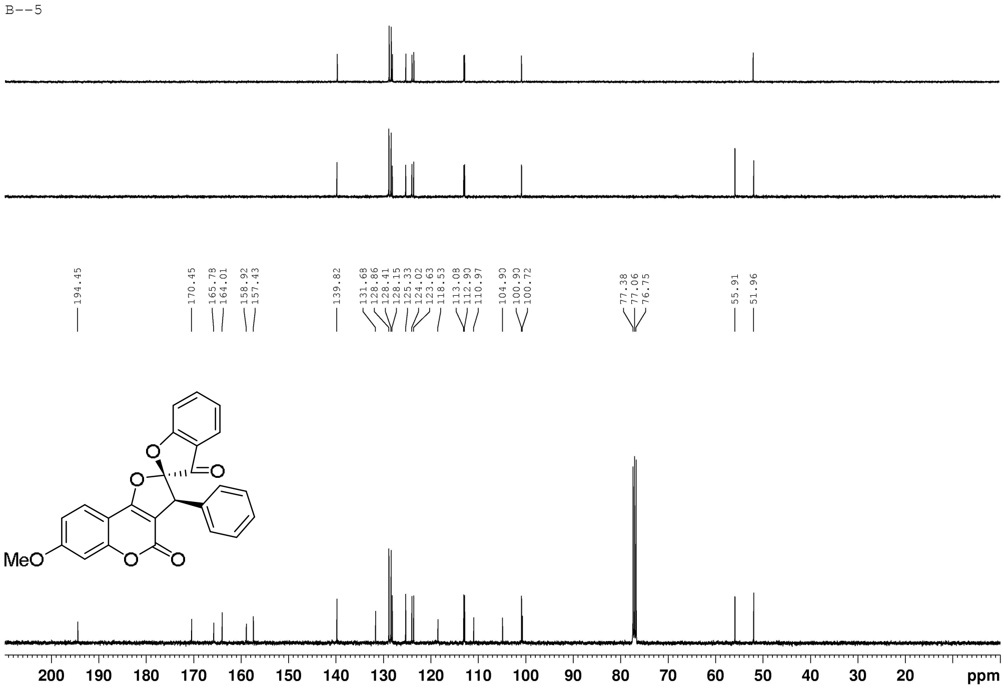


**Figure S8.** $^{13}C$ NMR (100 MHz, $CDCl_3$) spectra of compound **3ad**

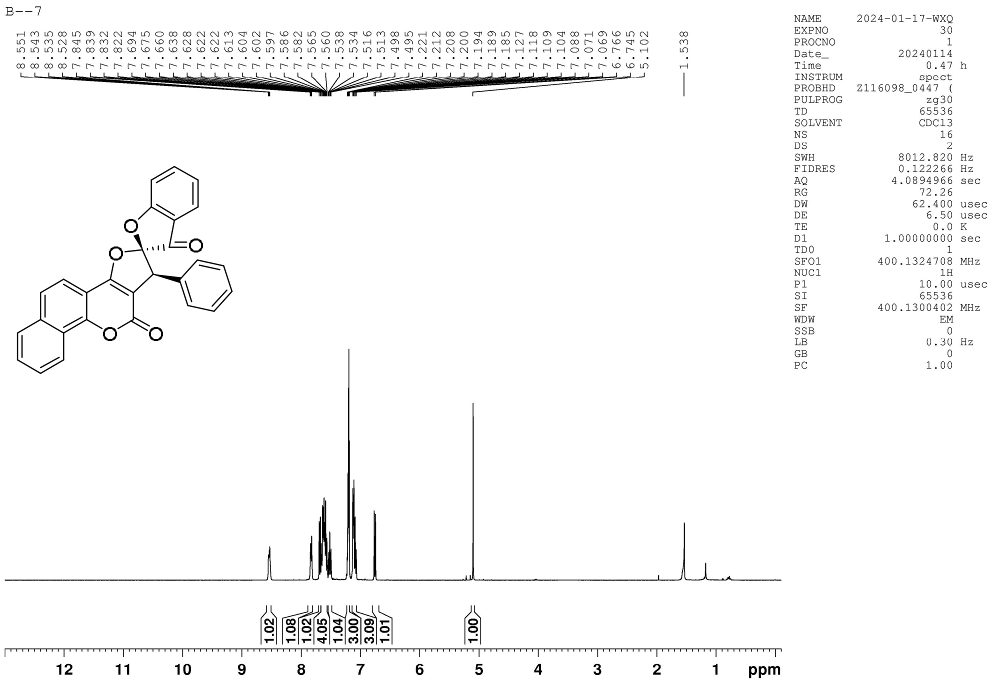


**Figure S9.** $^{1}$H NMR (400 MHz, $CDCl_3$) spectra of compound **3ae**

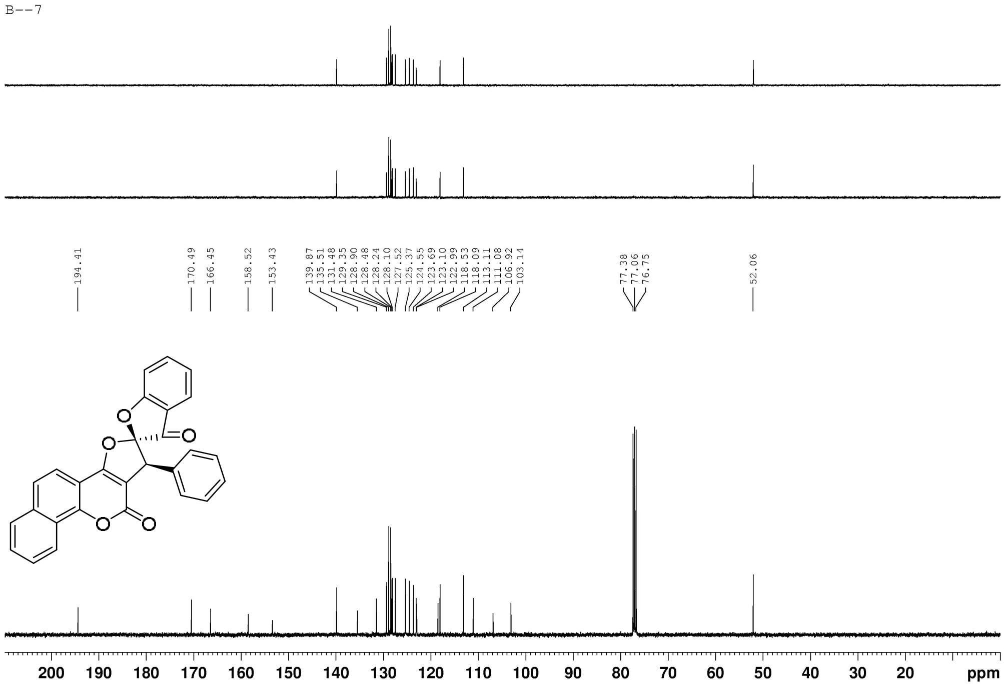


**Figure S10.** $^{13}$C NMR (100 MHz, $CDCl_3$) spectra of compound **3ae**

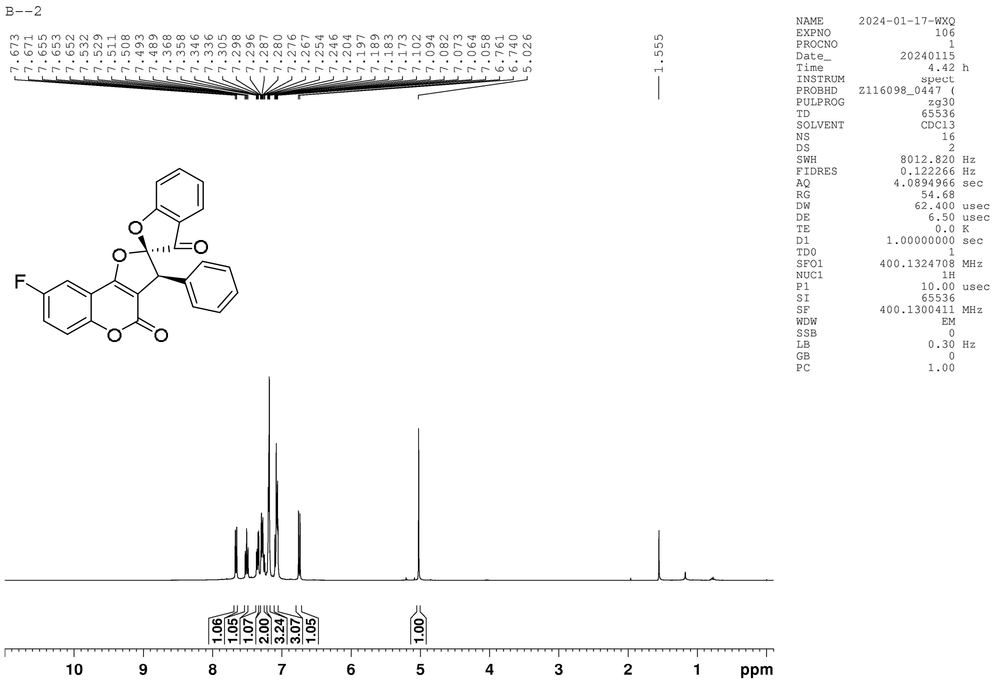


**Figure S11.** $^{1}$H NMR (400 MHz, $CDCl_3$) spectra of compound **3af**

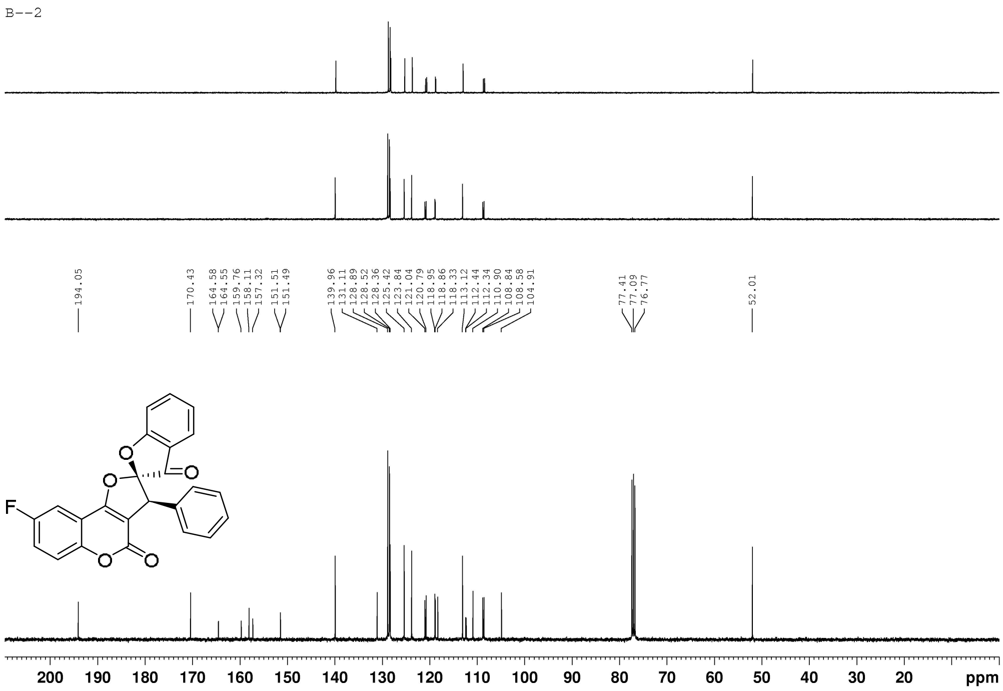


**Figure S12.** $^{13}$C NMR (100 MHz, $CDCl_3$) spectra of compound **3af**

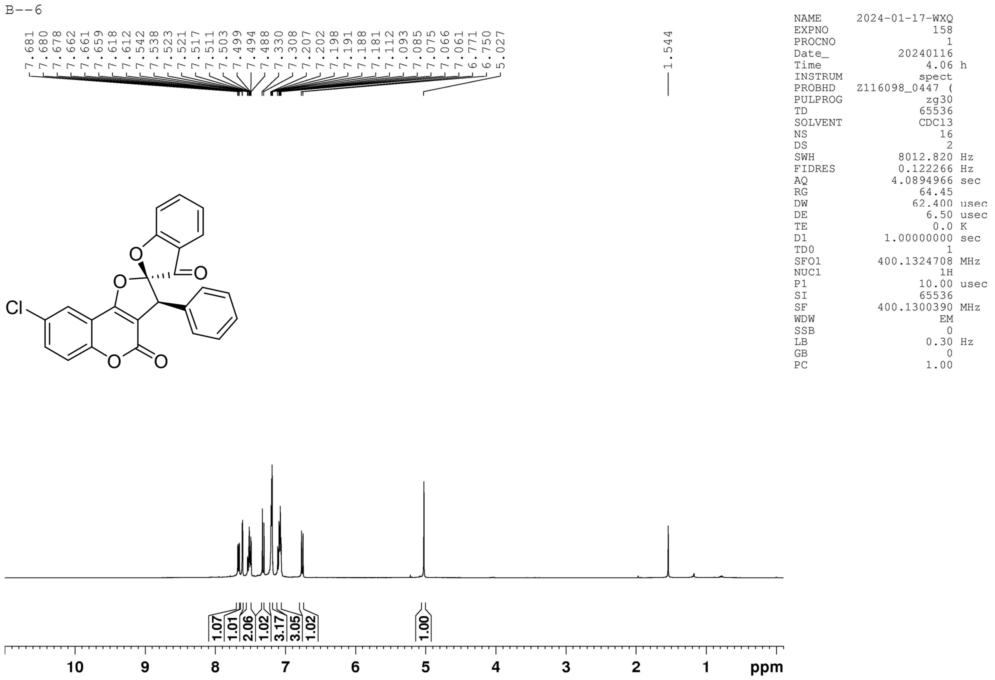


**Figure S13.** $^{1}$H NMR (400 MHz, $CDCl_3$) spectra of compound **3ag**

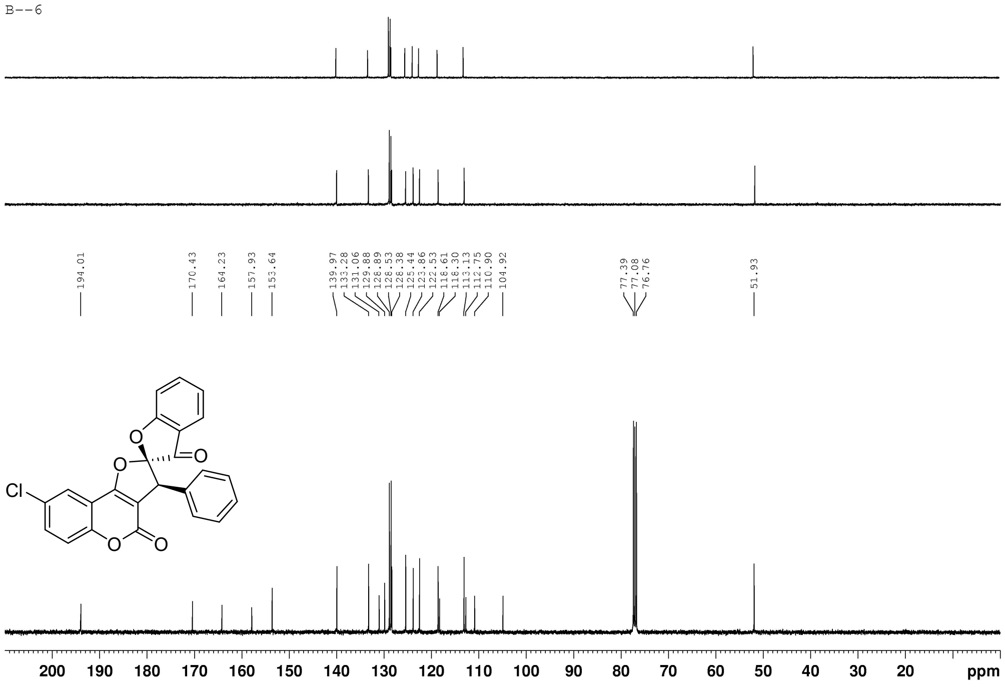


**Figure S14.** $^{13}$C NMR (100 MHz, $CDCl_3$) spectra of compound **3ag**

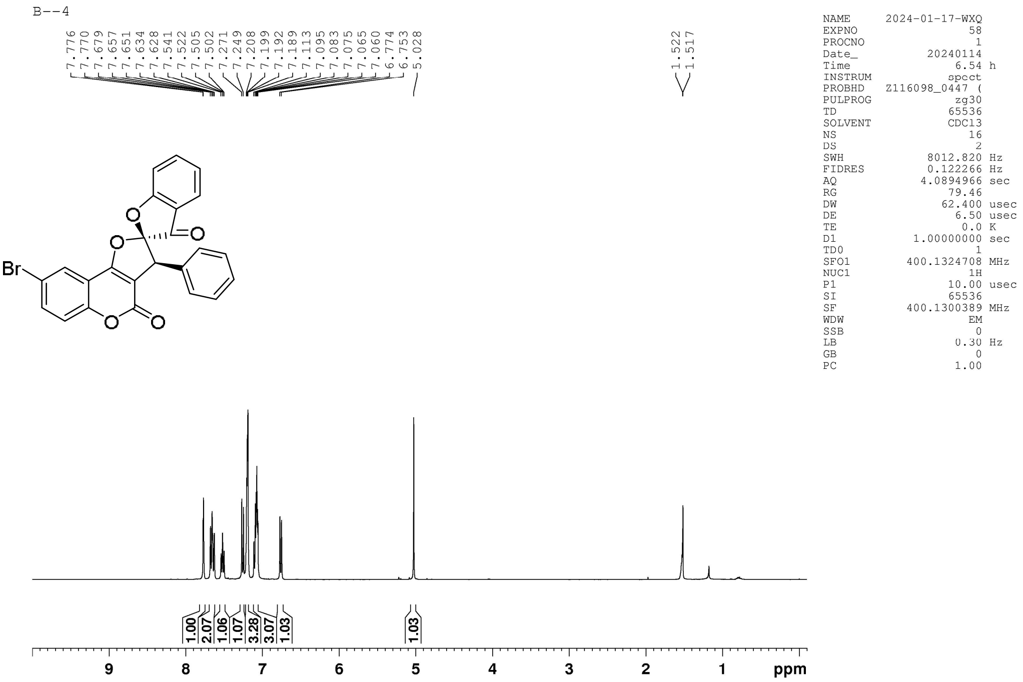


**Figure S15.** $^{1}$H NMR (400 MHz, $CDCl_3$) spectra of compound **3ah**

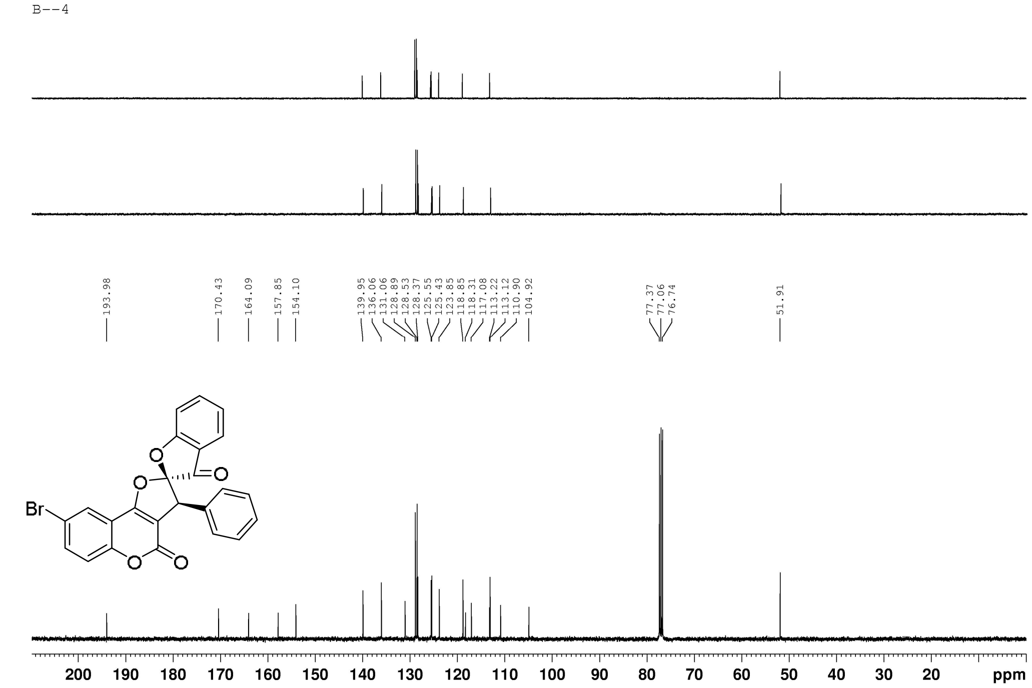


**Figure S16.** $^{13}$C NMR (100 MHz, $CDCl_3$) spectra of compound **3ah**

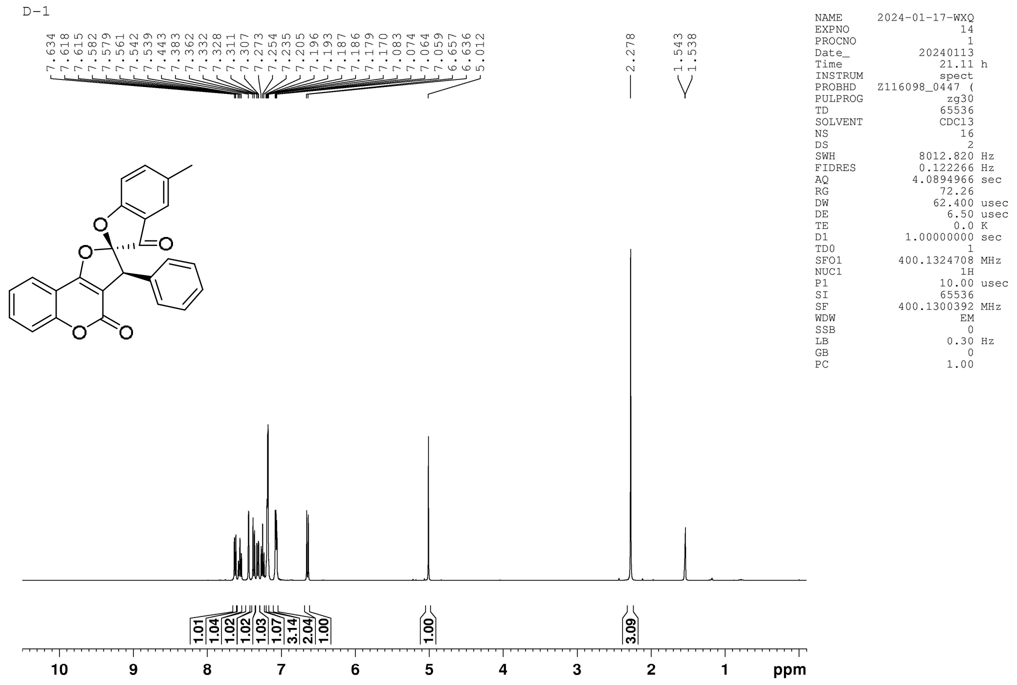


**Figure S17.** $^{1}$H NMR (400 MHz, $CDCl_3$) spectra of compound **3ai**

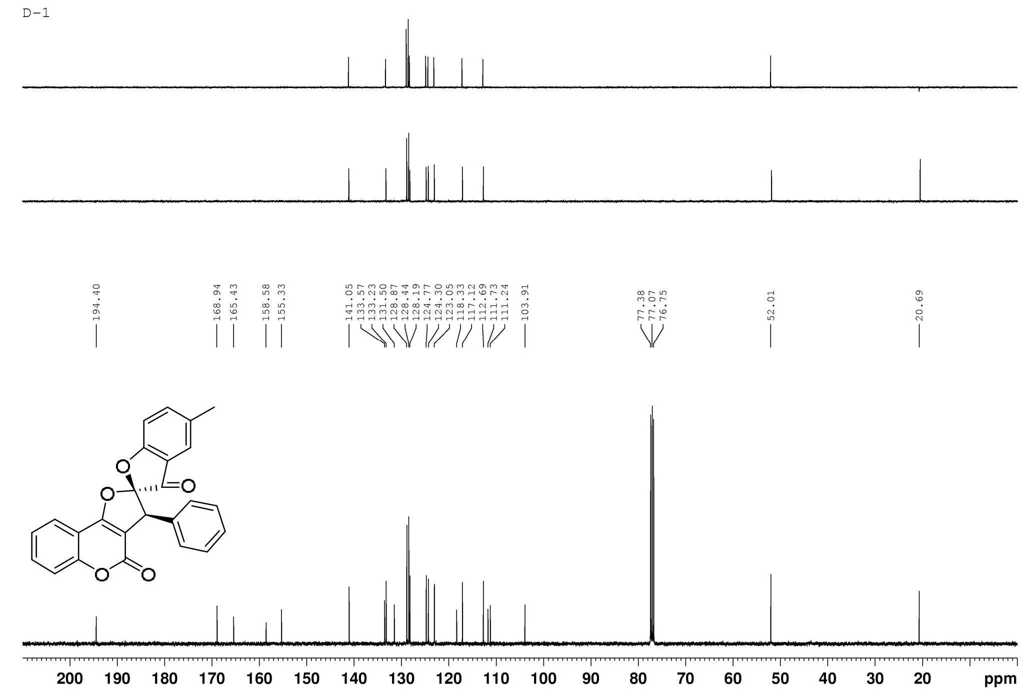


**Figure S18.** $^{13}$C NMR (100 MHz, $CDCl_3$) spectra of compound **3ai**

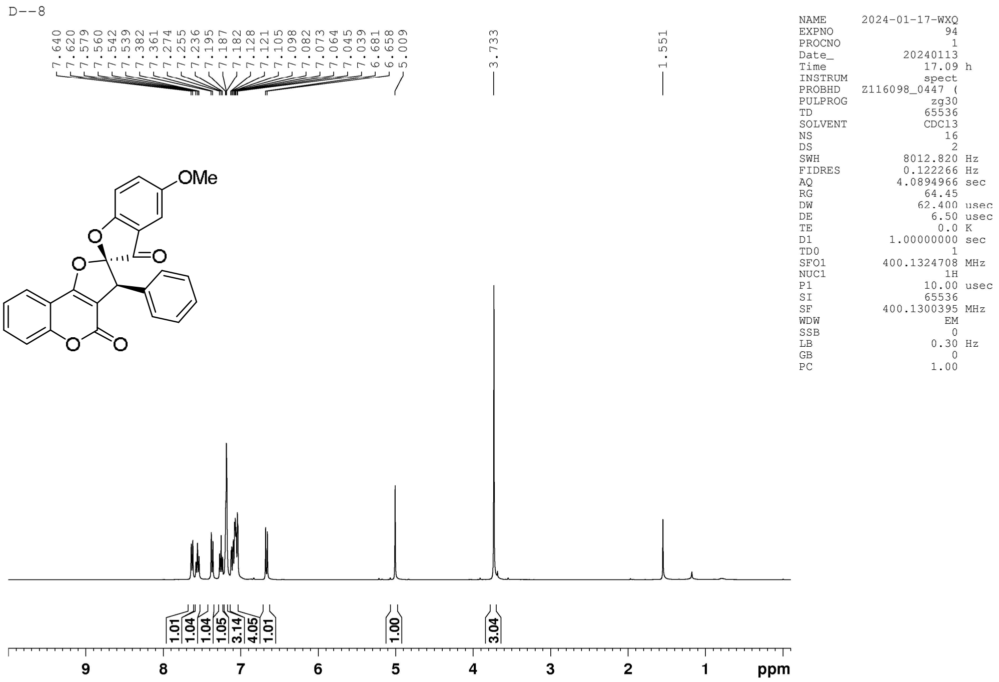


**Figure S19.** $^{1}H$ NMR (400 MHz, $CDCl_3$) spectra of compound **3aj**

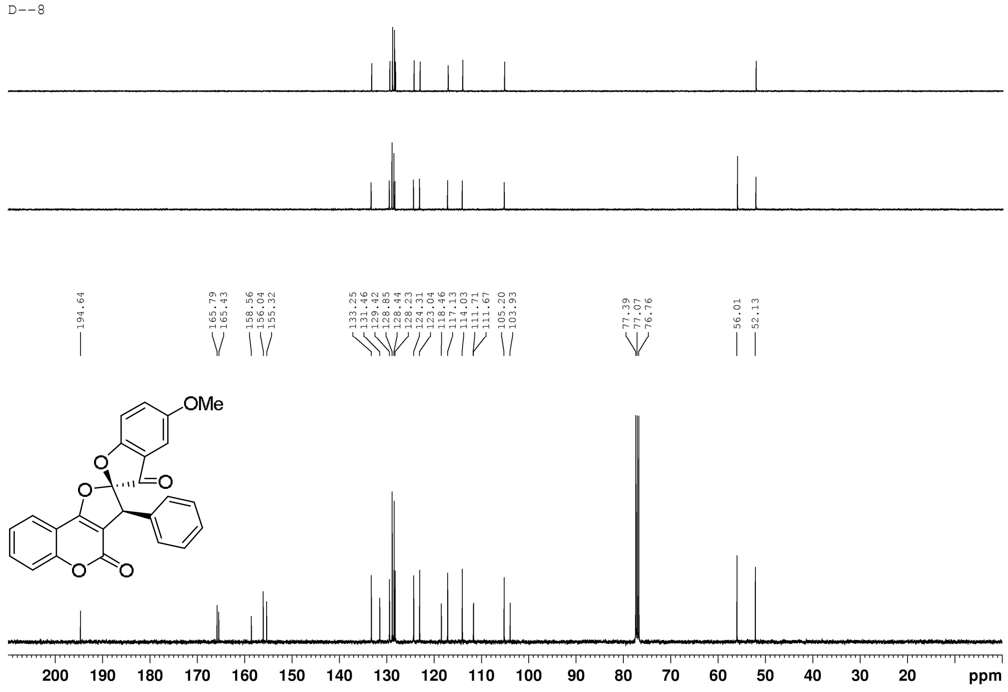


**Figure S20.** $^{13}C$ NMR (100 MHz, $CDCl_3$) spectra of compound **3aj**

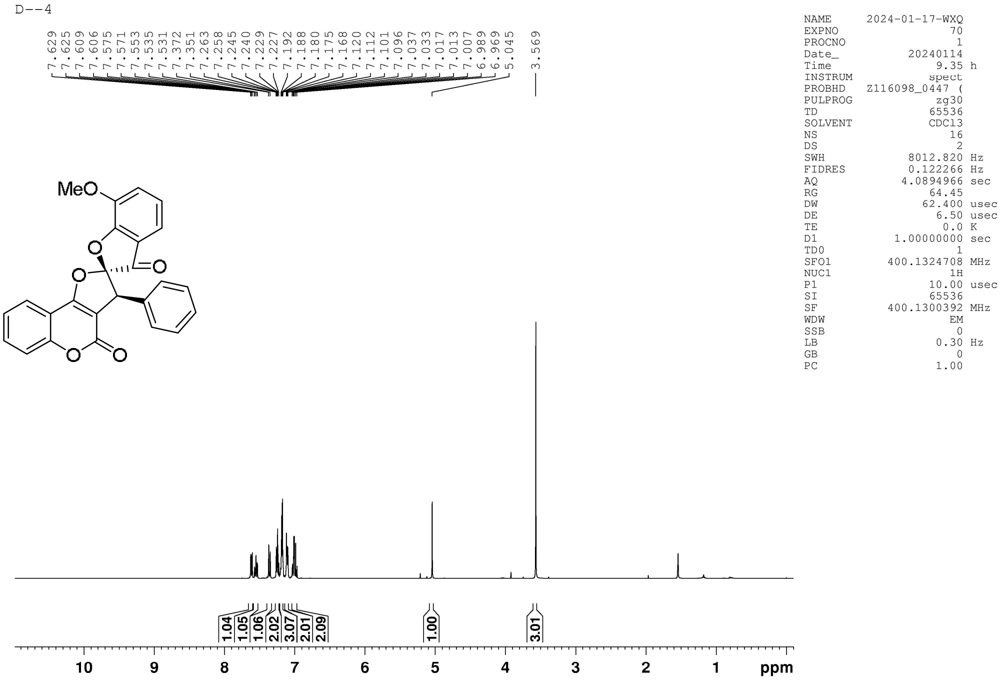


**Figure S21.** $^{1}$H NMR (400 MHz, $CDCl_3$) spectra of compound **3ak**

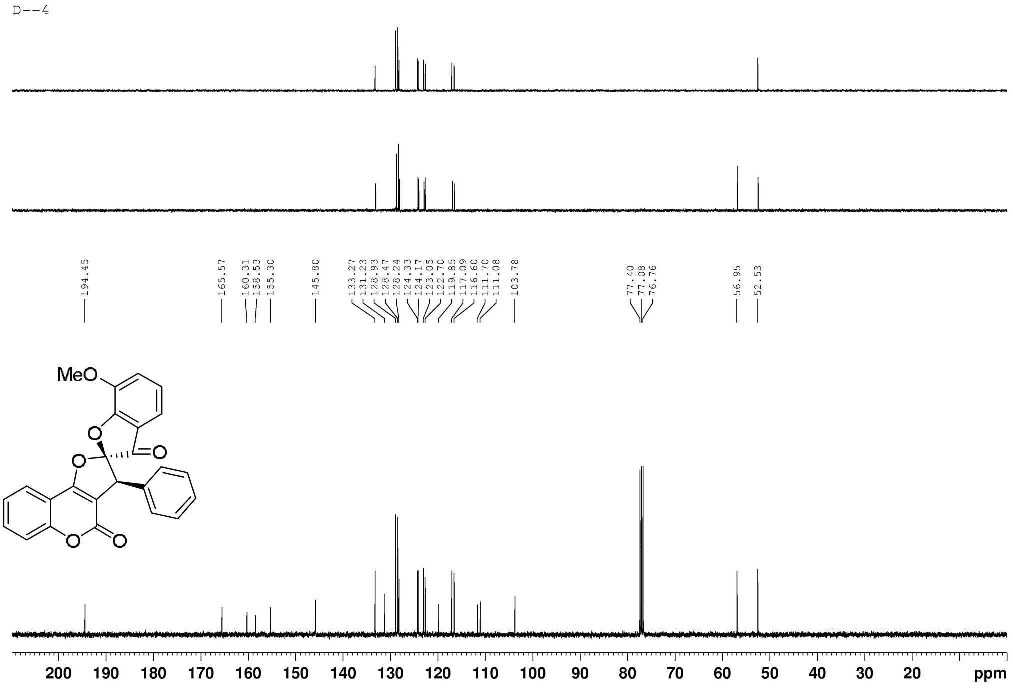


**Figure S22.** $^{13}$C NMR (100 MHz, $CDCl_3$) spectra of compound **3ak**

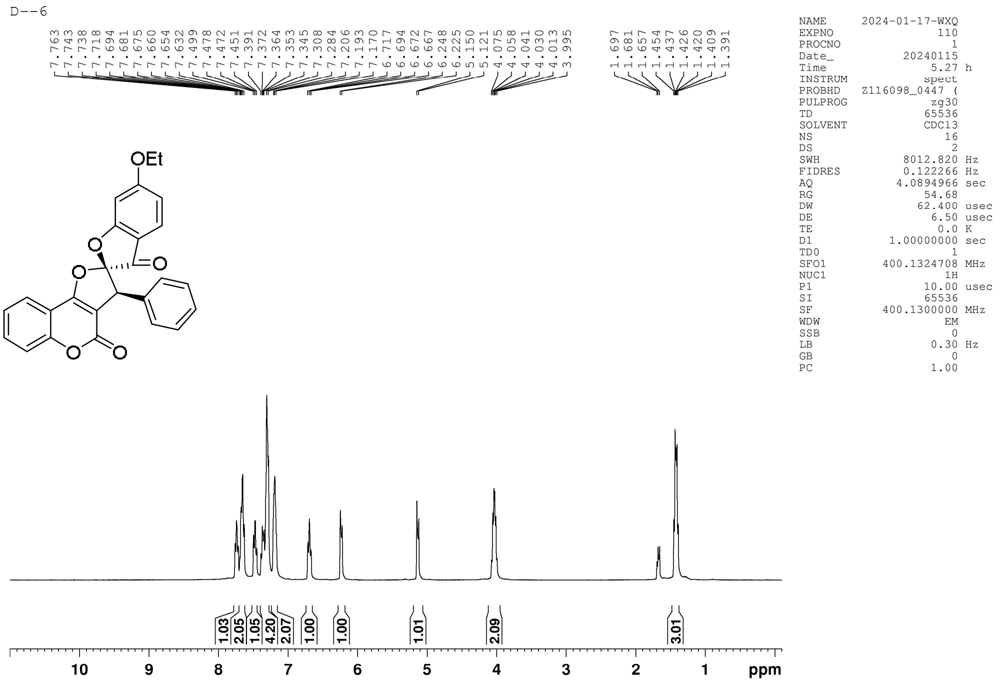


**Figure S23.** $^{1}$H NMR (400 MHz, $CDCl_3$) spectra of compound **3al**

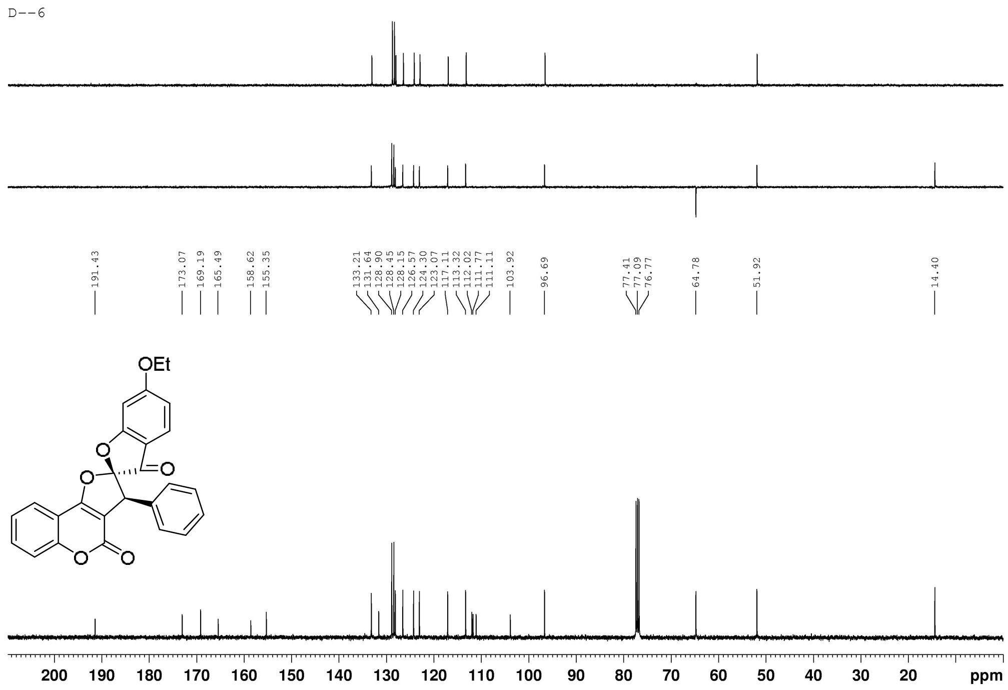


**Figure S24.** $^{13}$C NMR (100 MHz, $CDCl_3$) spectra of compound **3al**

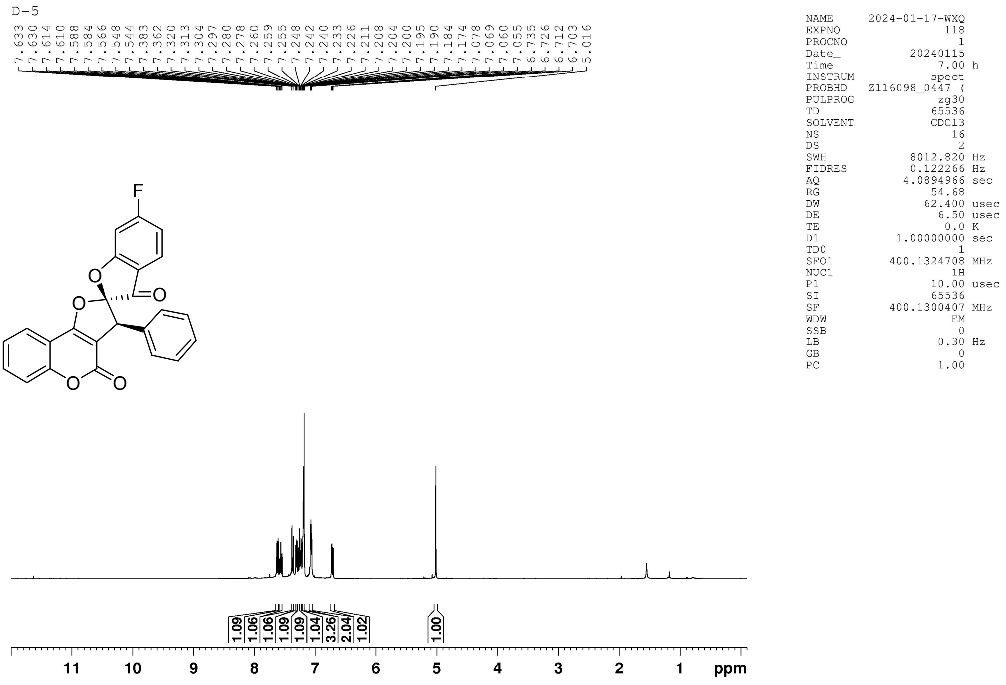

**Figure S25.** $^{1}$H NMR (400 MHz, $CDCl_3$) spectra of compound **3am**

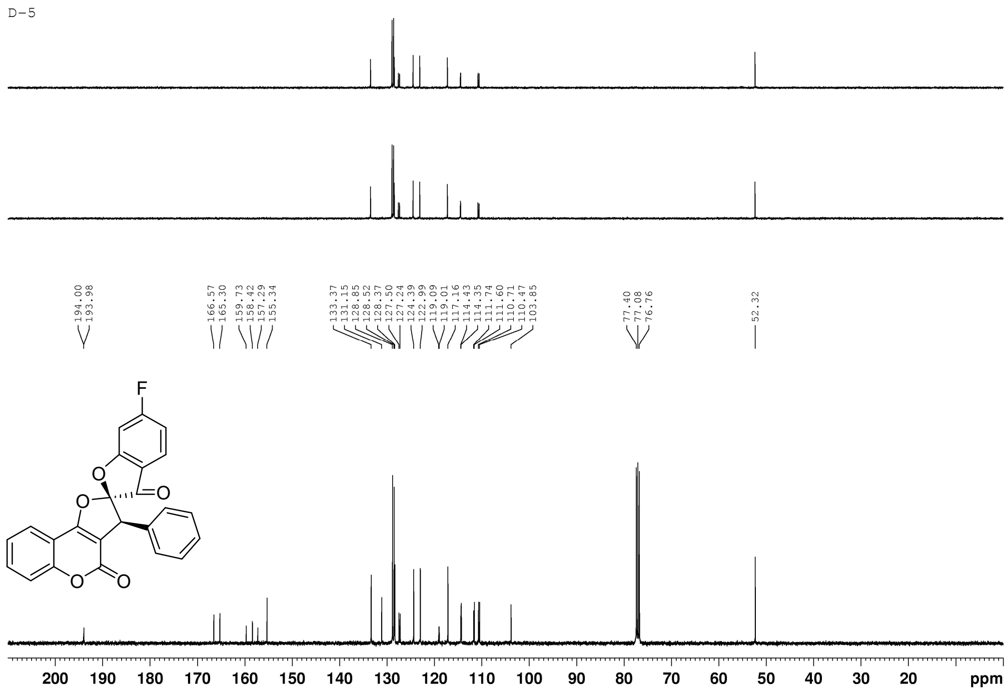

**Figure S26.** $^{13}$C NMR (100 MHz, $CDCl_3$) spectra of compound **3am**

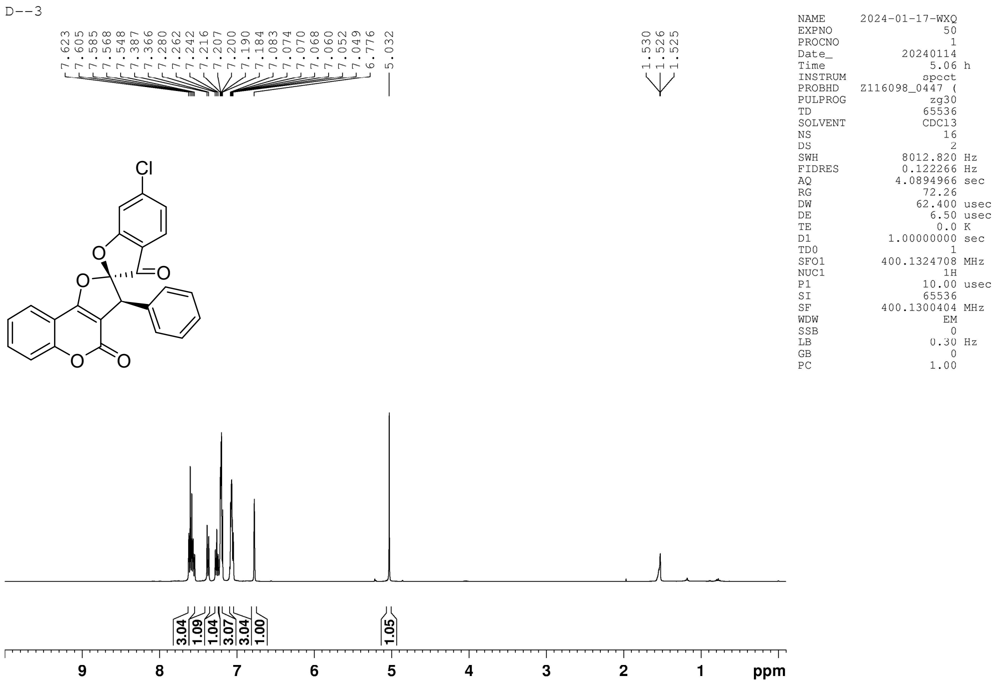


**Figure S27.** $^{1}$H NMR (400 MHz, $CDCl_3$) spectra of compound **3an**

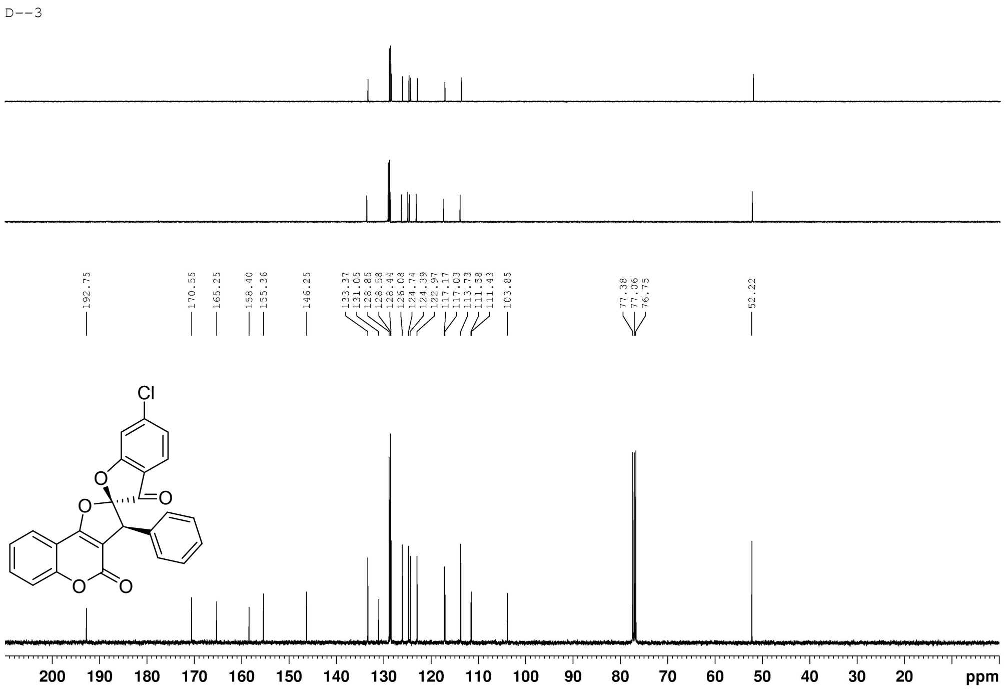


**Figure S28.** $^{13}$C NMR (100 MHz, $CDCl_3$) spectra of compound **3an**

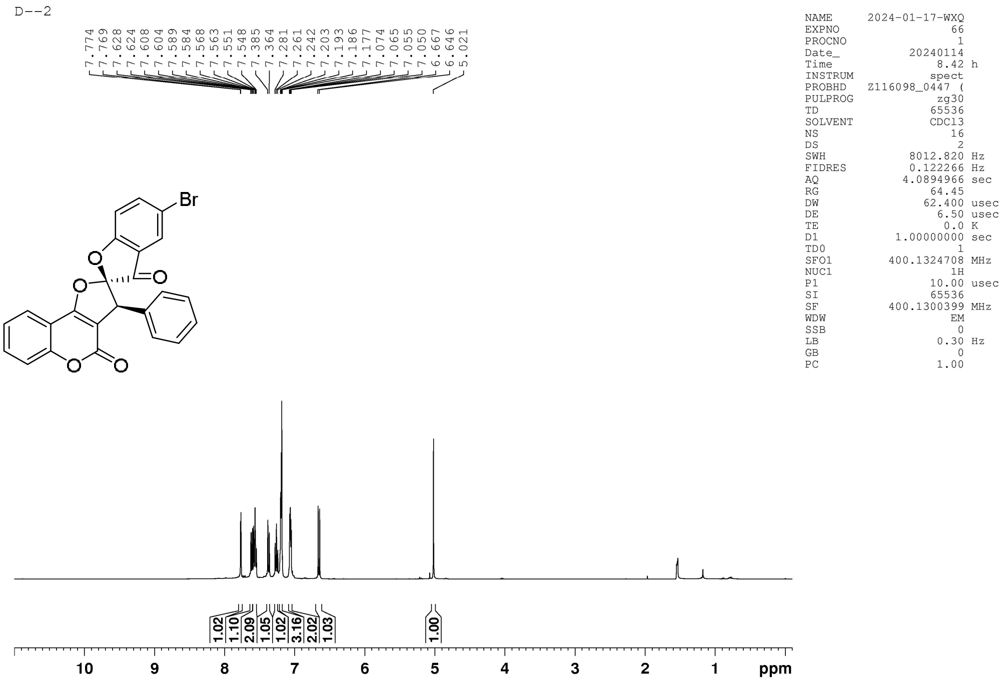

**Figure S29.** $^{1}$H NMR (400 MHz, $CDCl_3$) spectra of compound **3ao**

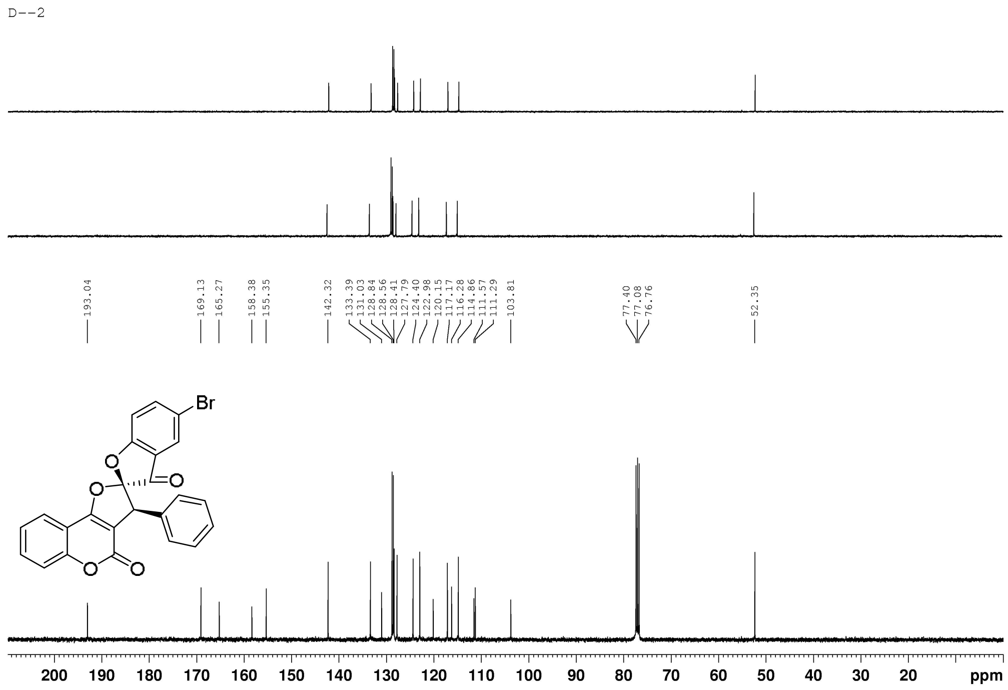

**Figure S30.** $^{13}$C NMR (100 MHz, $CDCl_3$) spectra of compound **3ao**

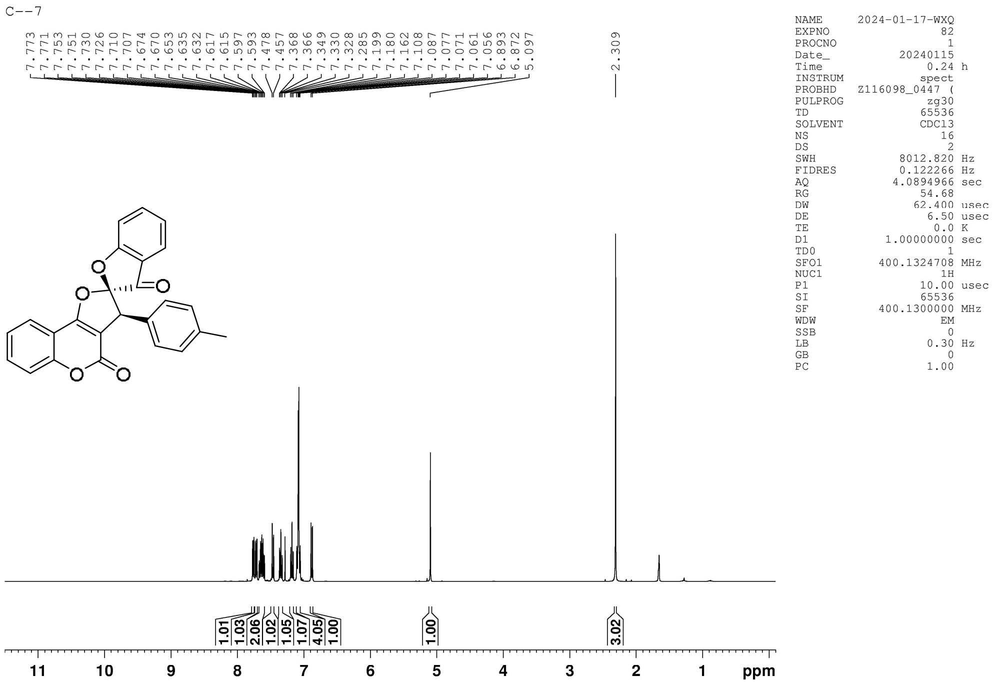


**Figure S31.** $^{1}$H NMR (400 MHz, $CDCl_3$) spectra of compound **3ap**

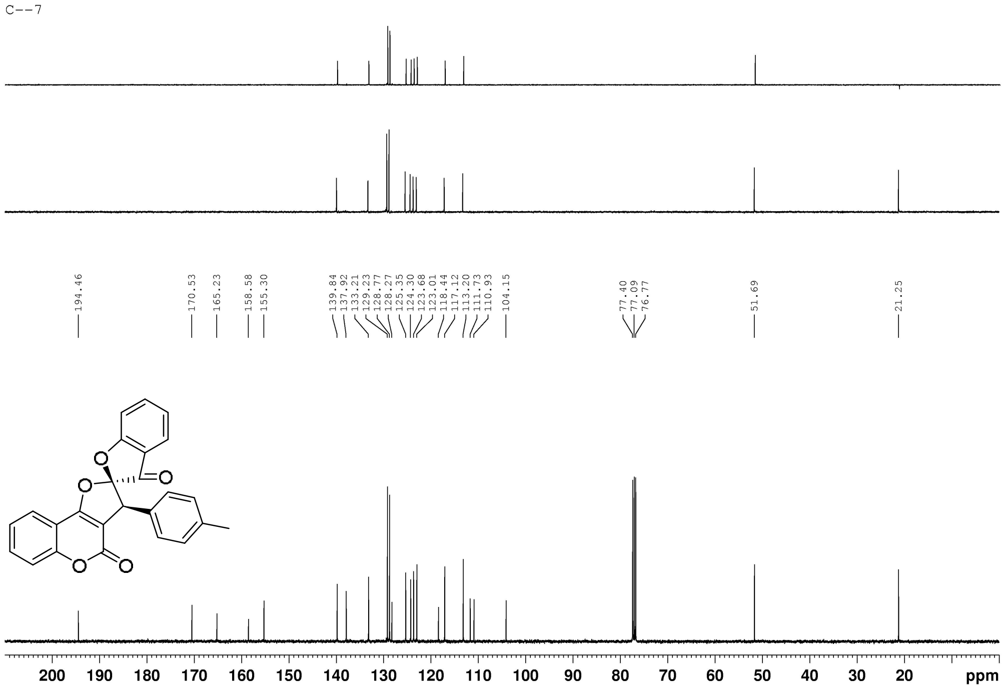


**Figure S32.** $^{13}$C NMR (100 MHz, $CDCl_3$) spectra of compound **3ap**

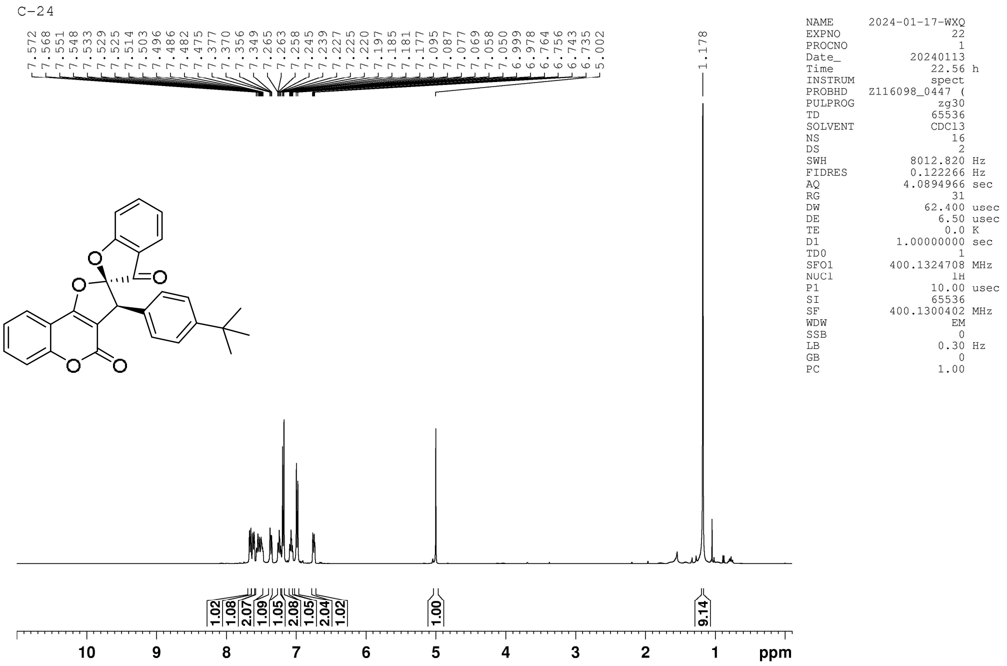


**Figure S33.** $^{1}H$ NMR (400 MHz, $CDCl_3$) spectra of compound **3aq**

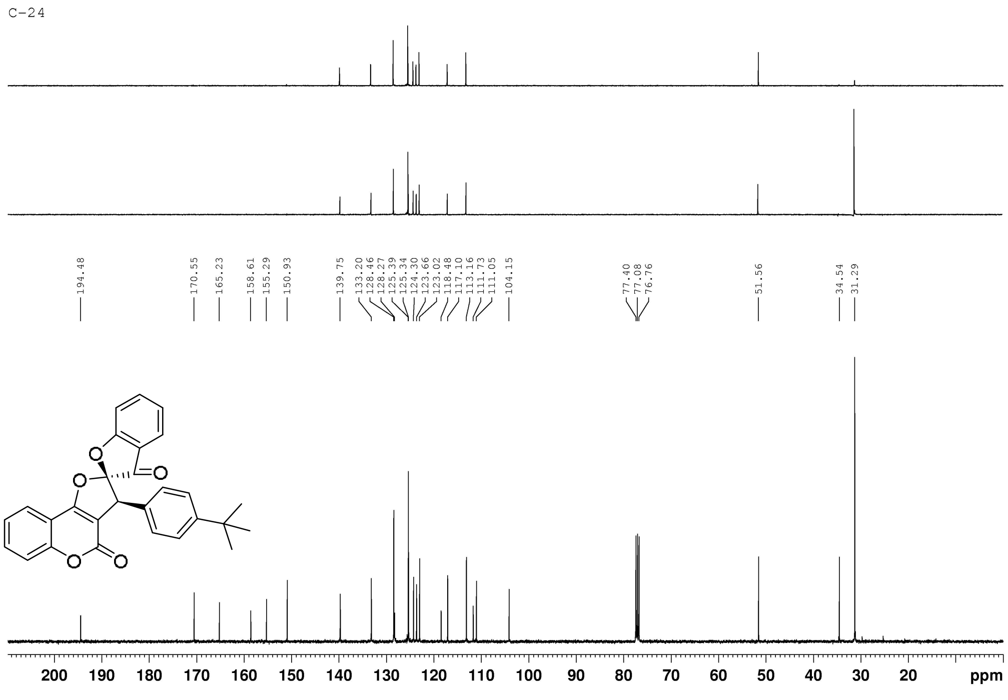


**Figure S34.** $^{13}C$ NMR (100 MHz, $CDCl_3$) spectra of compound **3aq**

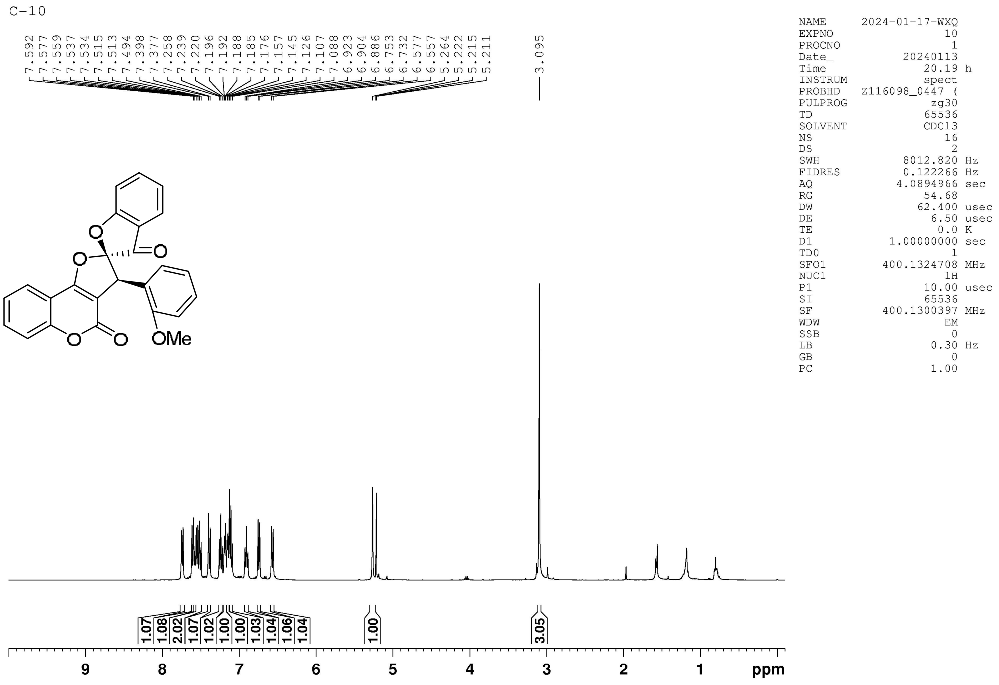

**Figure S35.** $^{1}$H NMR (400 MHz, $CDCl_3$) spectra of compound **3ar**

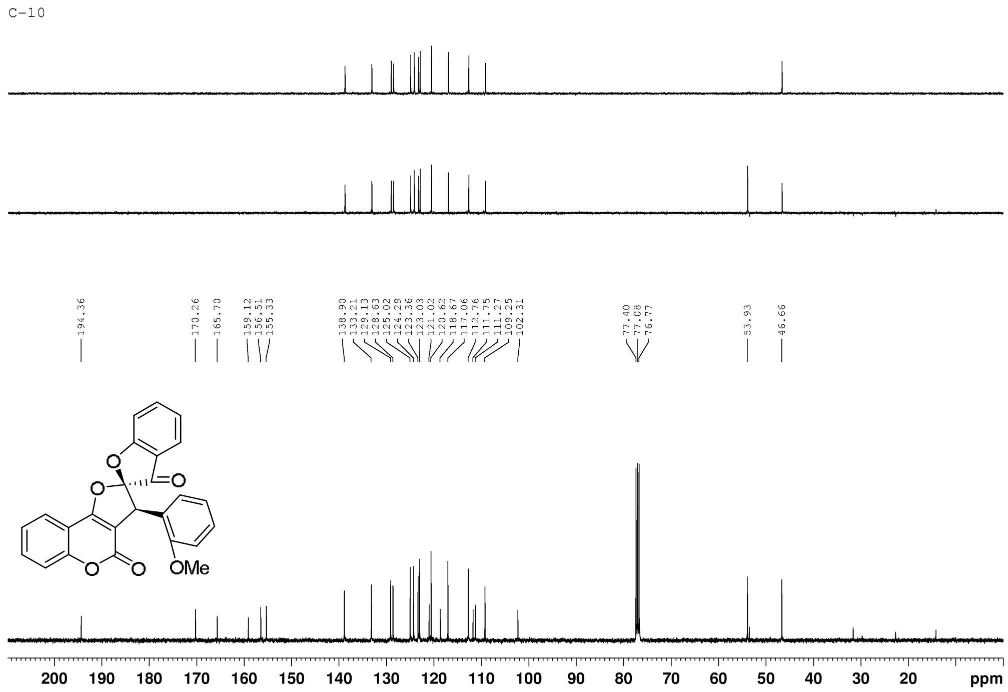

**Figure S36.** $^{13}$C NMR (100 MHz, $CDCl_3$) spectra of compound **3ar**

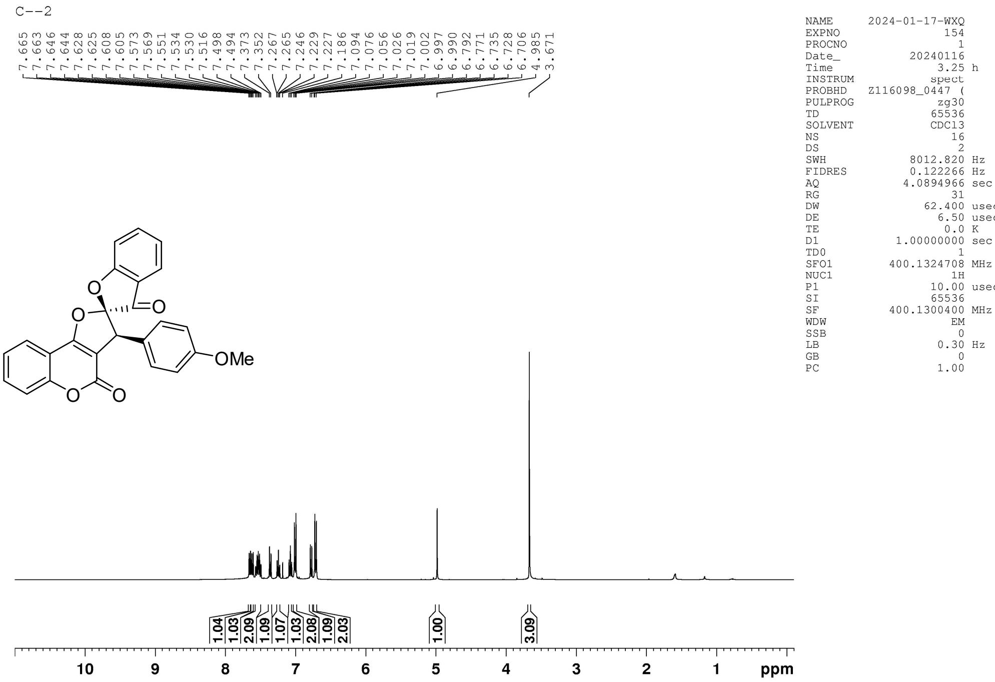

**Figure S37.** $^{1}H$ NMR (400 MHz, $CDCl_3$) spectra of compound **3as**

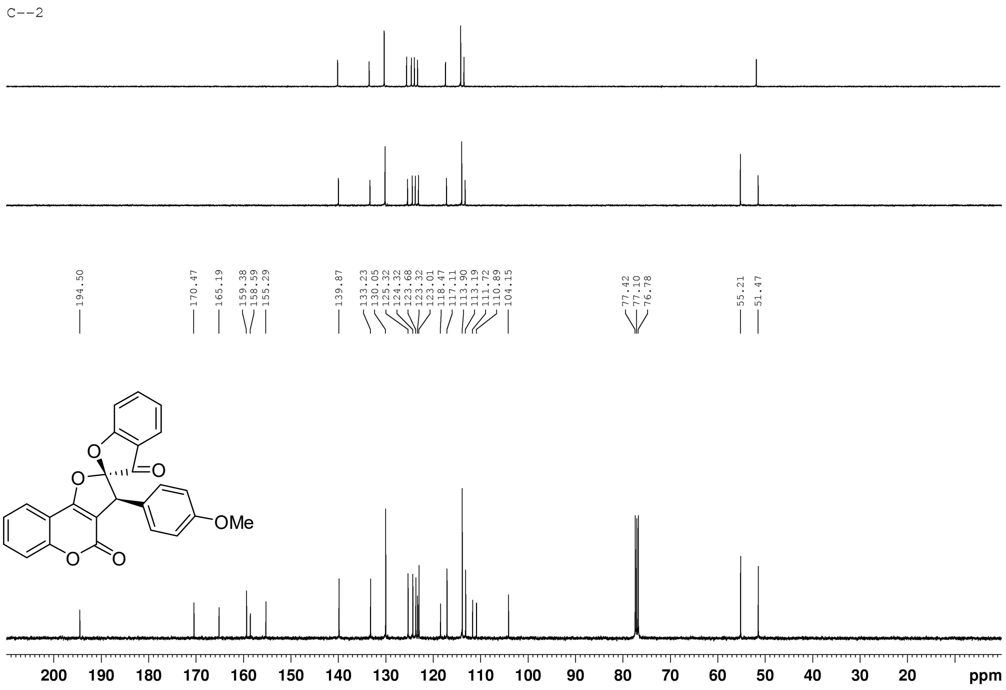

**Figure S38.** $^{13}C$ NMR (100 MHz, $CDCl_3$) spectra of compound **3as**

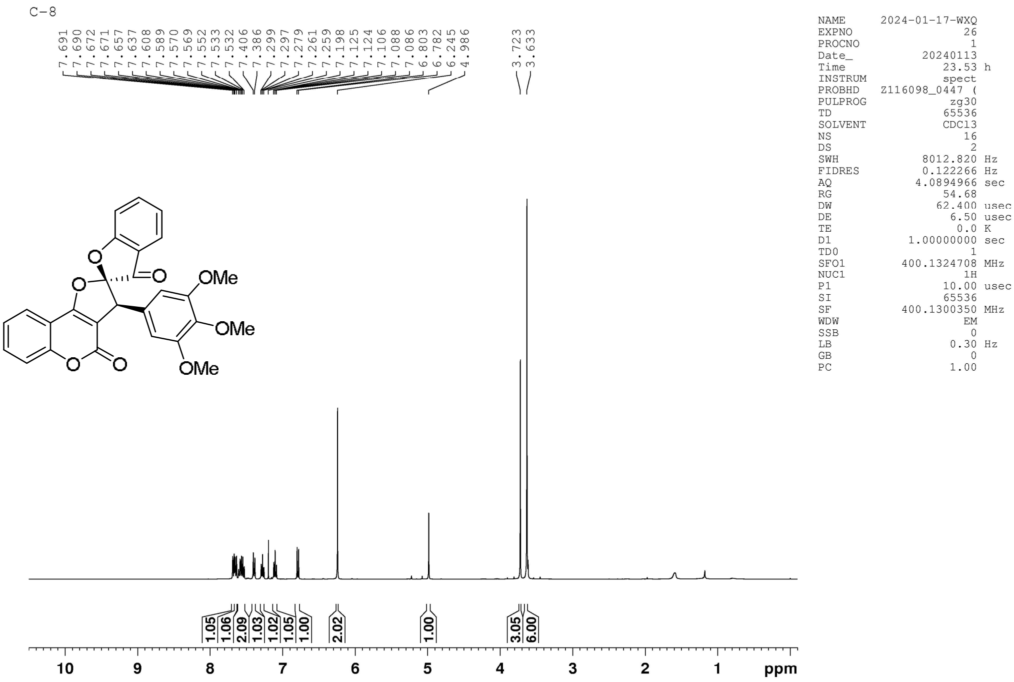


**Figure S39.** $^{1}$H NMR (400 MHz, $CDCl_3$) spectra of compound **3at**

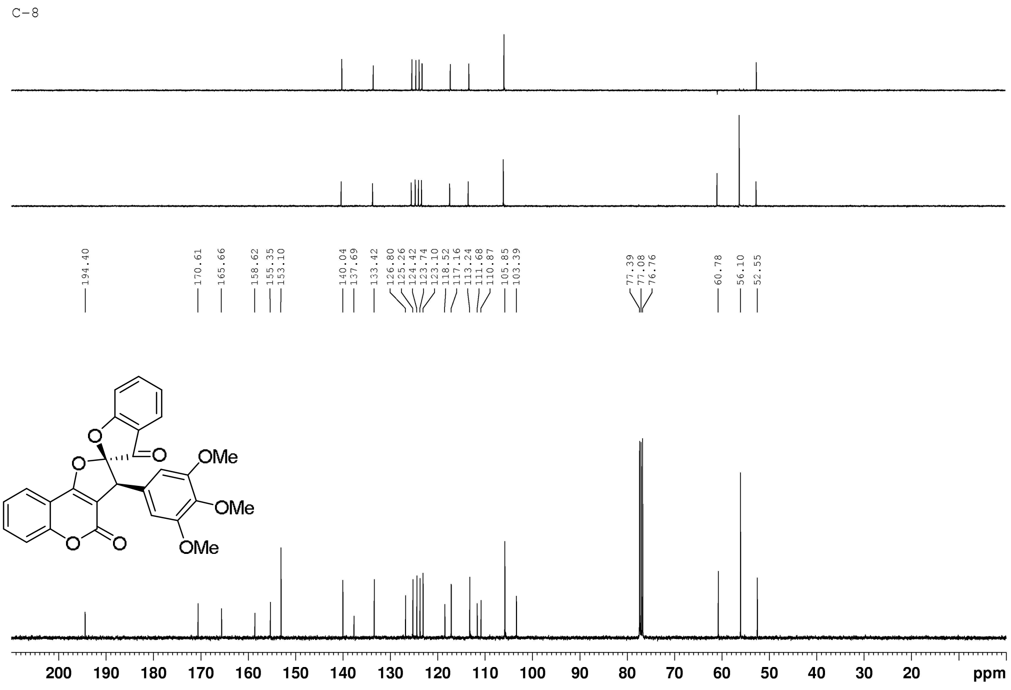


**Figure S40.** $^{13}$C NMR (100 MHz, $CDCl_3$) spectra of compound **3at**

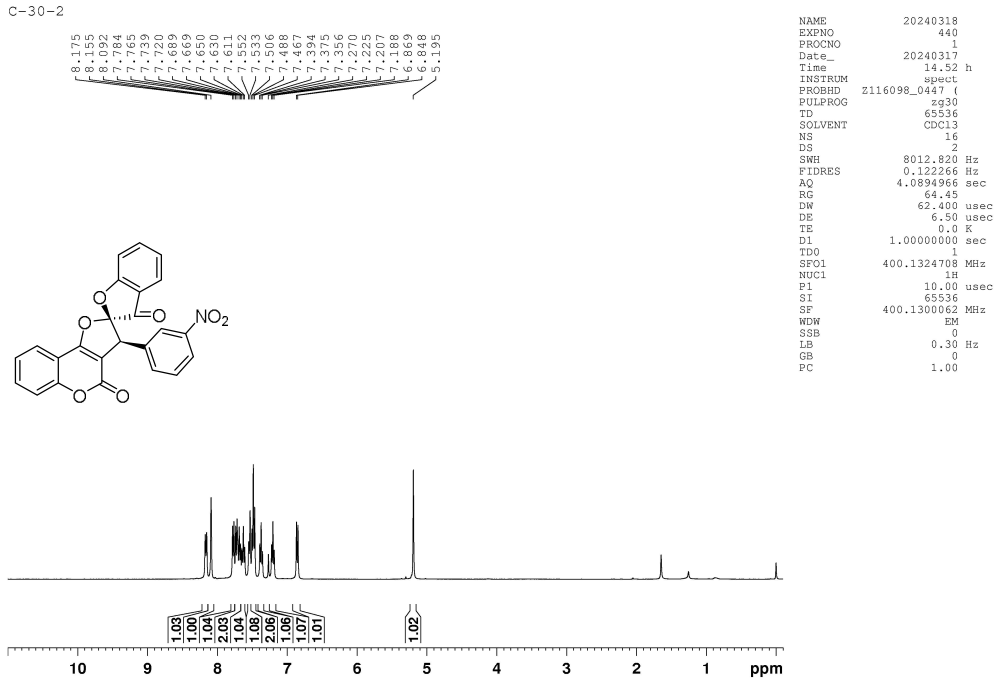


**Figure S41.** $^{1}$H NMR (400 MHz, $CDCl_3$) spectra of compound **3av**

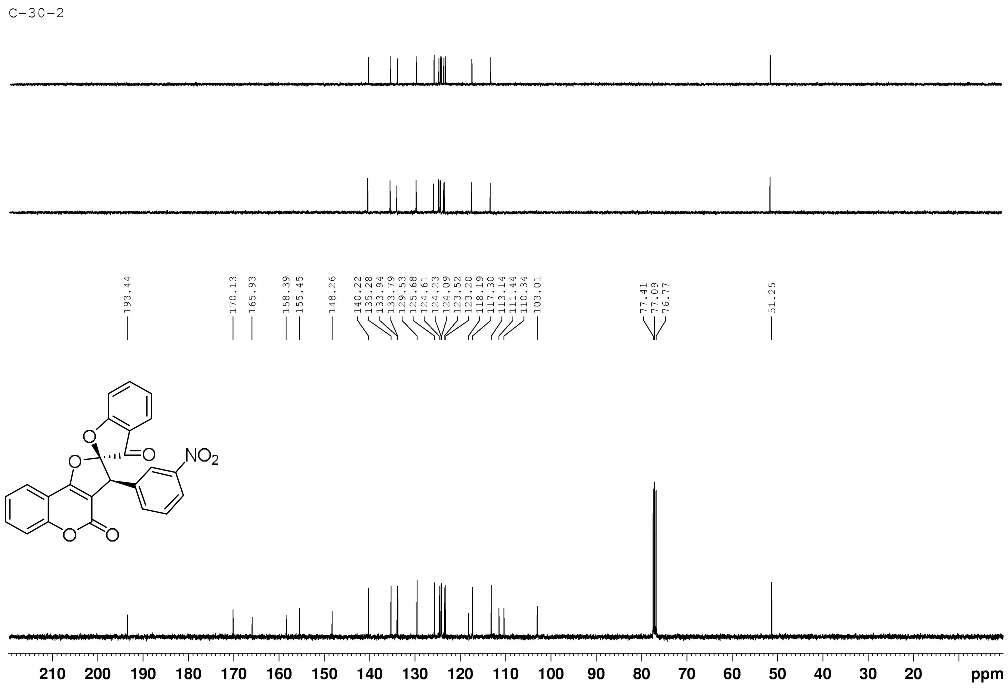


**Figure S42.** $^{13}$C NMR (100 MHz, $CDCl_3$) spectra of compound **3av**

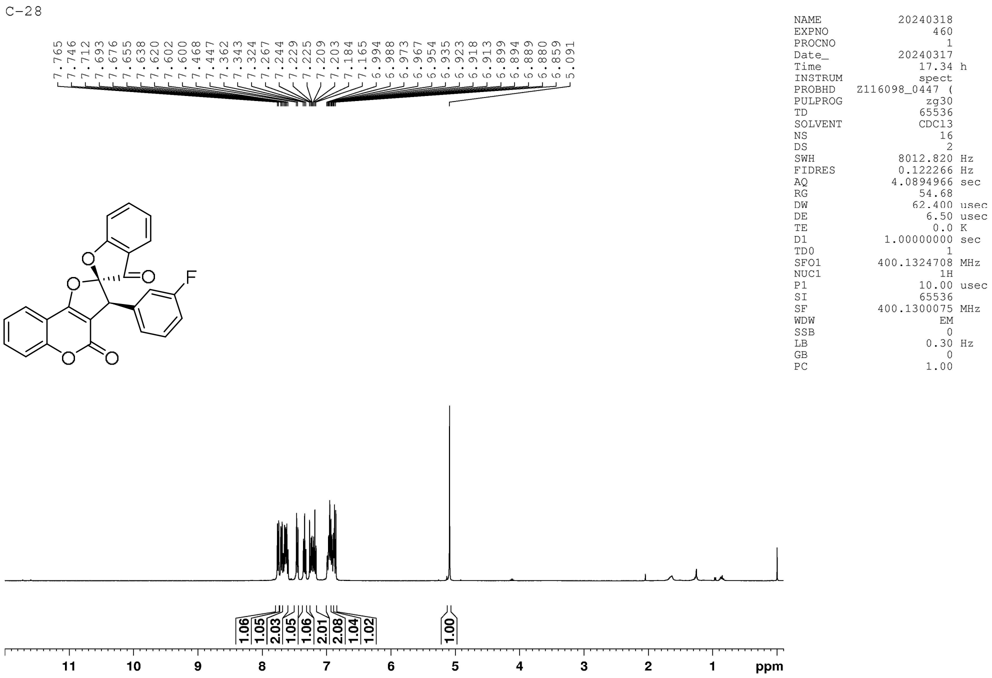

**Figure S43.** $^{1}$H NMR (400 MHz, $CDCl_3$) spectra of compound **3aw**

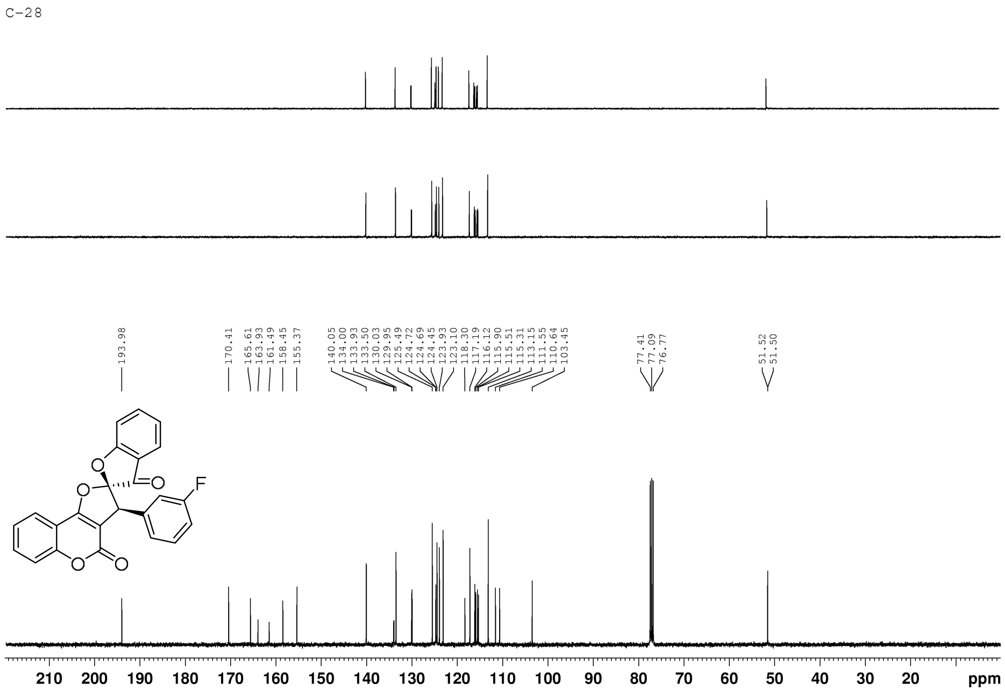

**Figure S44.** $^{13}$C NMR (100 MHz, $CDCl_3$) spectra of compound **3aw**

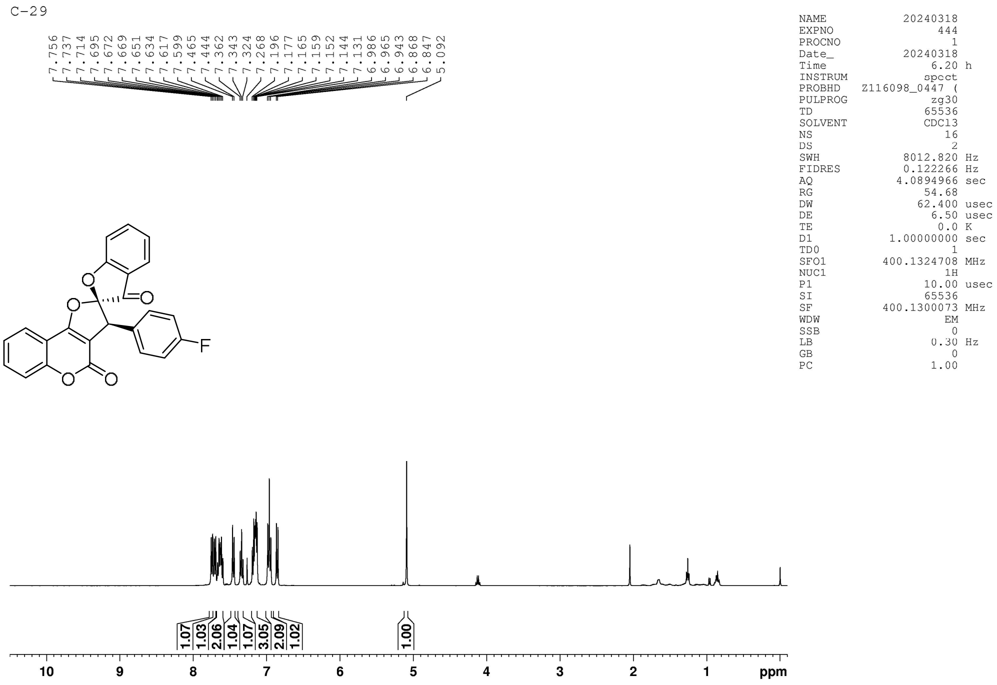

**Figure S45.** $^{1}$H NMR (400 MHz, $CDCl_3$) spectra of compound **3ay**

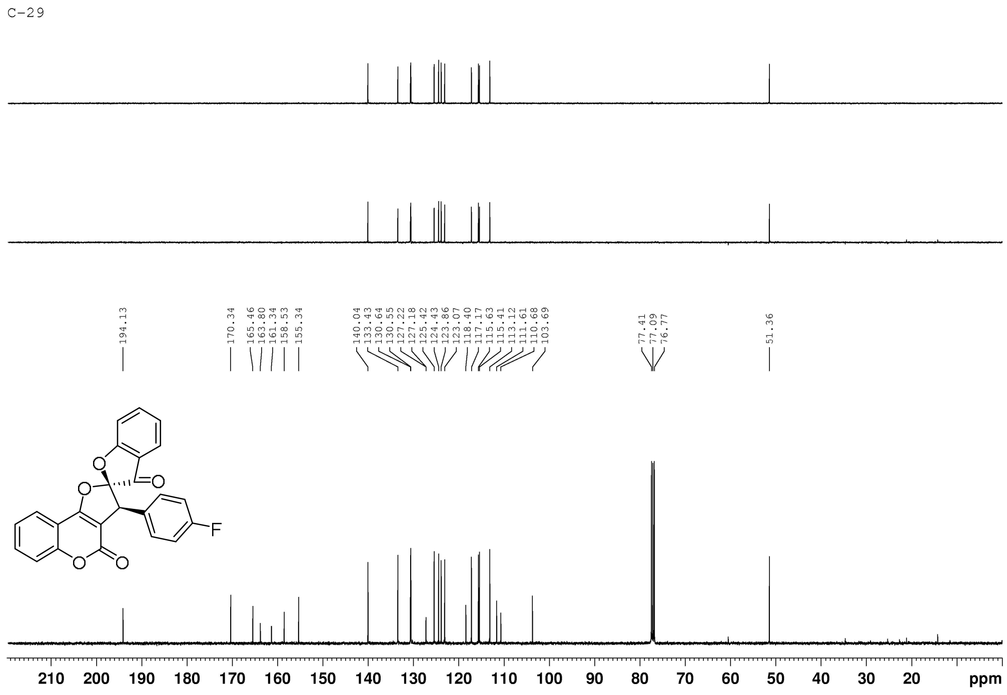

**Figure S46.** $^{13}$C NMR (100 MHz, $CDCl_3$) spectra of compound **3ay**

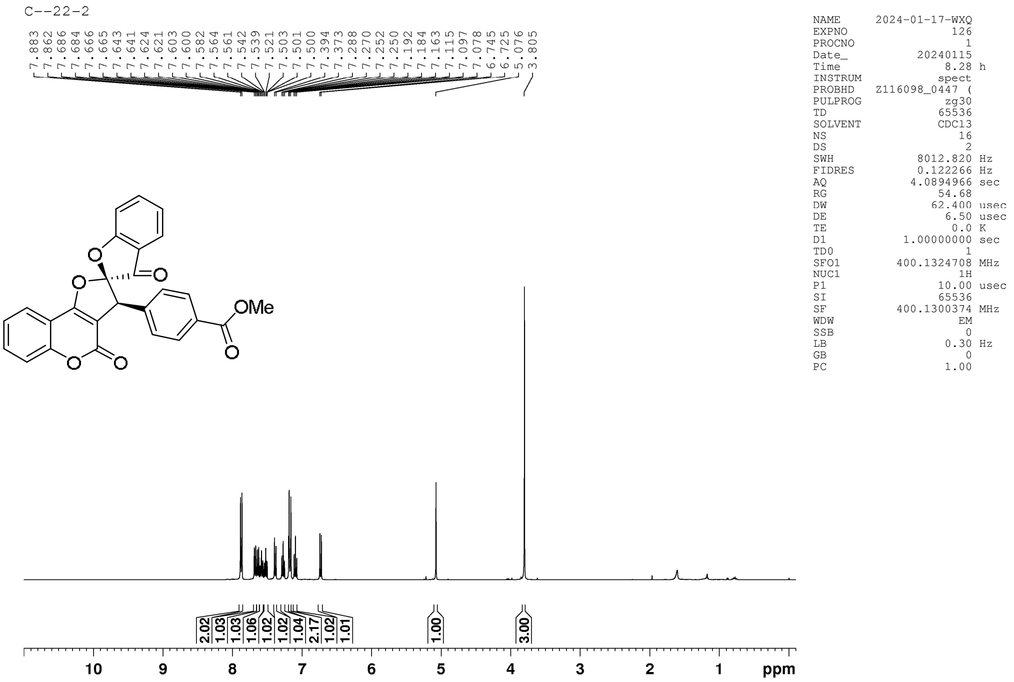


**Figure S47.** $^{1}H$ NMR (400 MHz, $CDCl_3$) spectra of compound **3az**

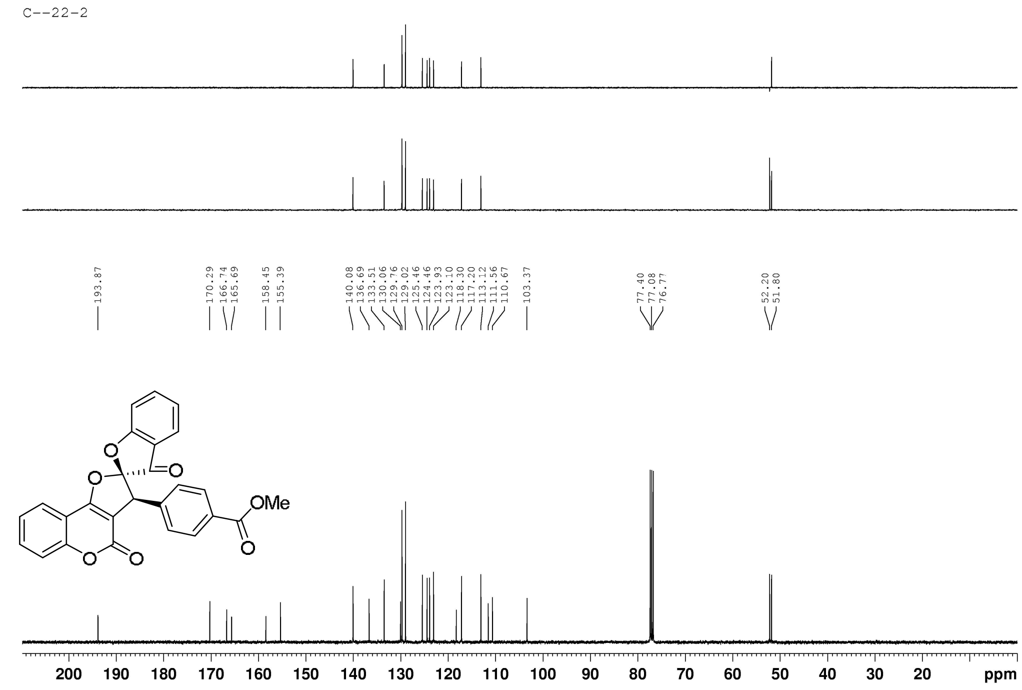


**Figure S48.** $^{13}C$ NMR (100 MHz, $CDCl_3$) spectra of compound **3az**

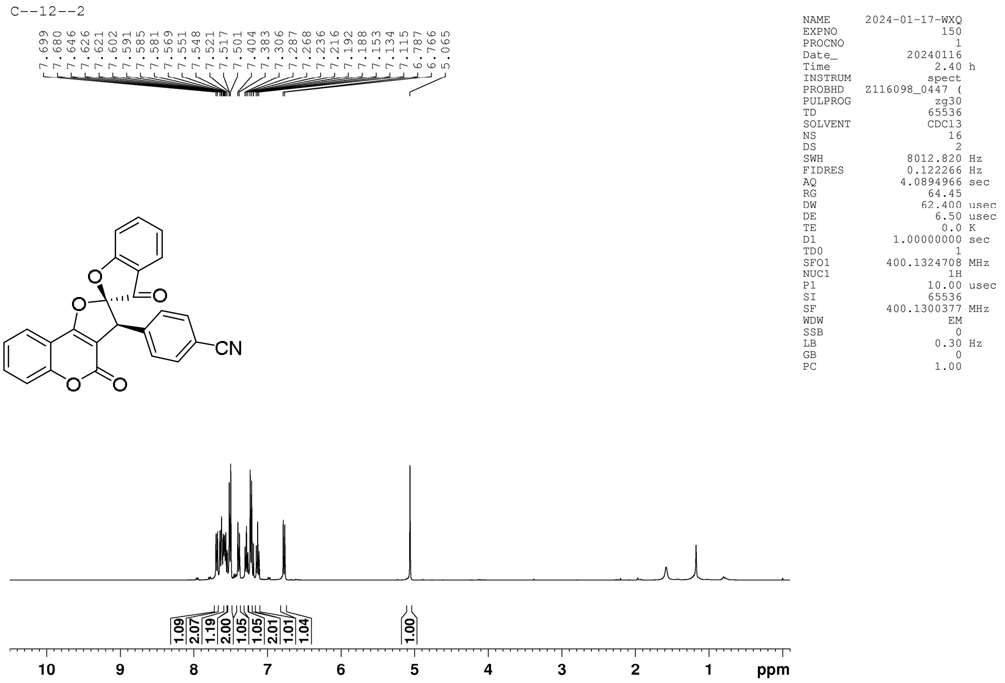


**Figure S49.** $^{1}$H NMR (400 MHz, $CDCl_3$) spectra of compound **3ba**

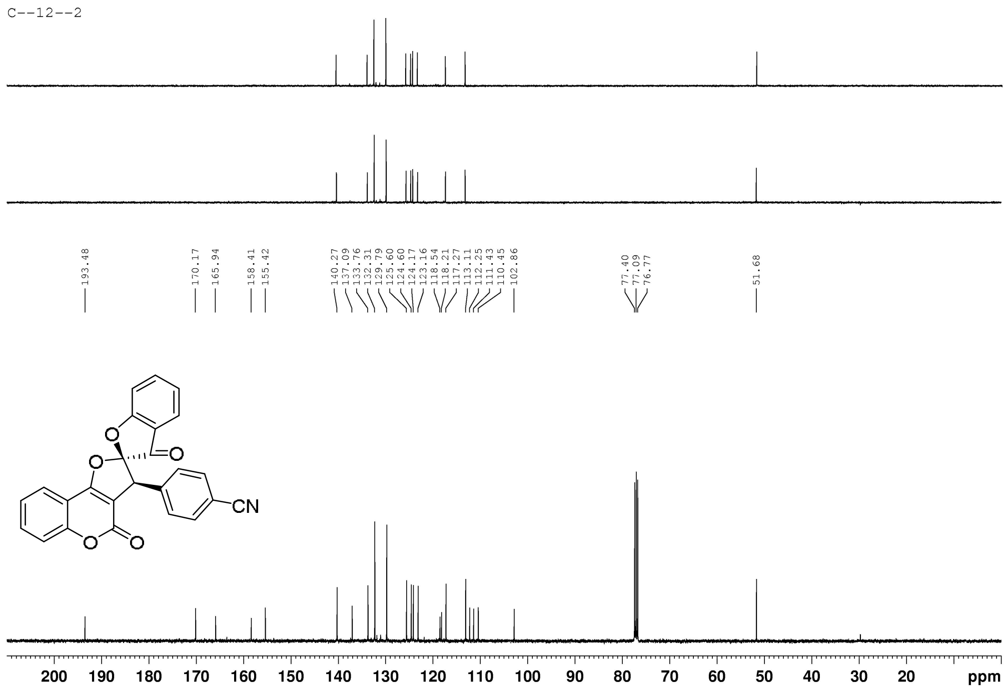


**Figure S50.** $^{13}$C NMR (100 MHz, $CDCl_3$) spectra of compound **3ba**

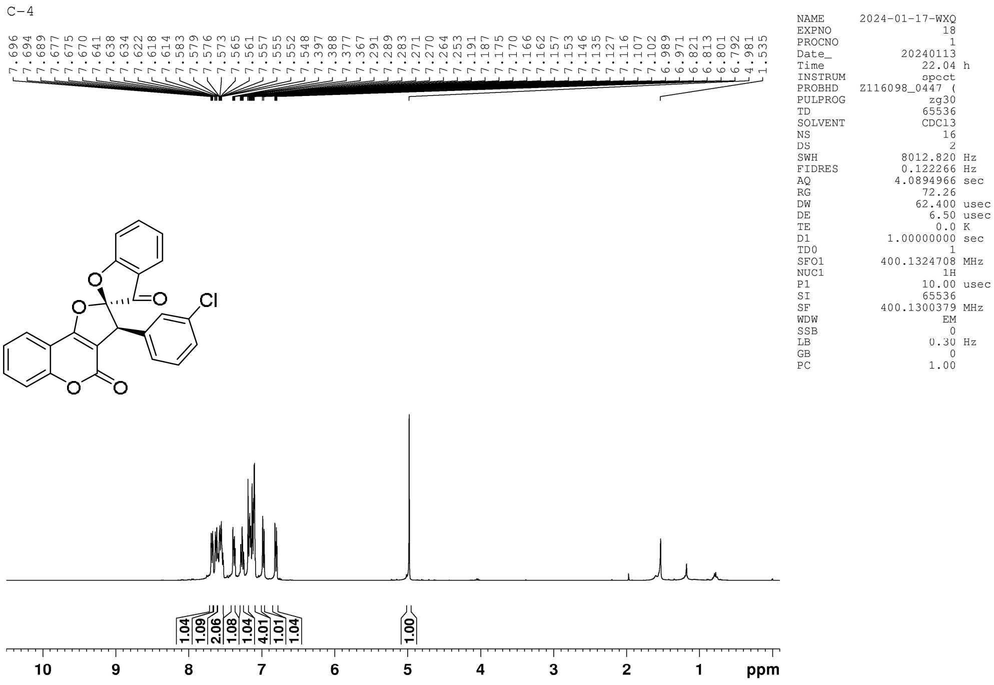


**Figure S51.** $^{1}H$ NMR (400 MHz, $CDCl_3$) spectra of compound **3bb**

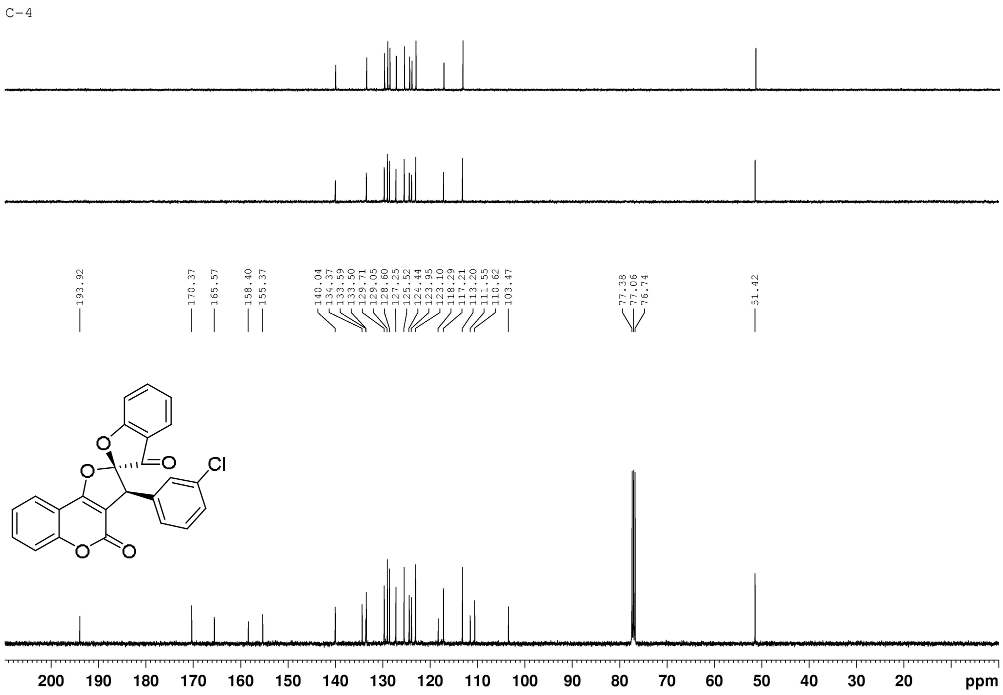


**Figure S52.** $^{13}C$ NMR (100 MHz, $CDCl_3$) spectra of compound **3bb**

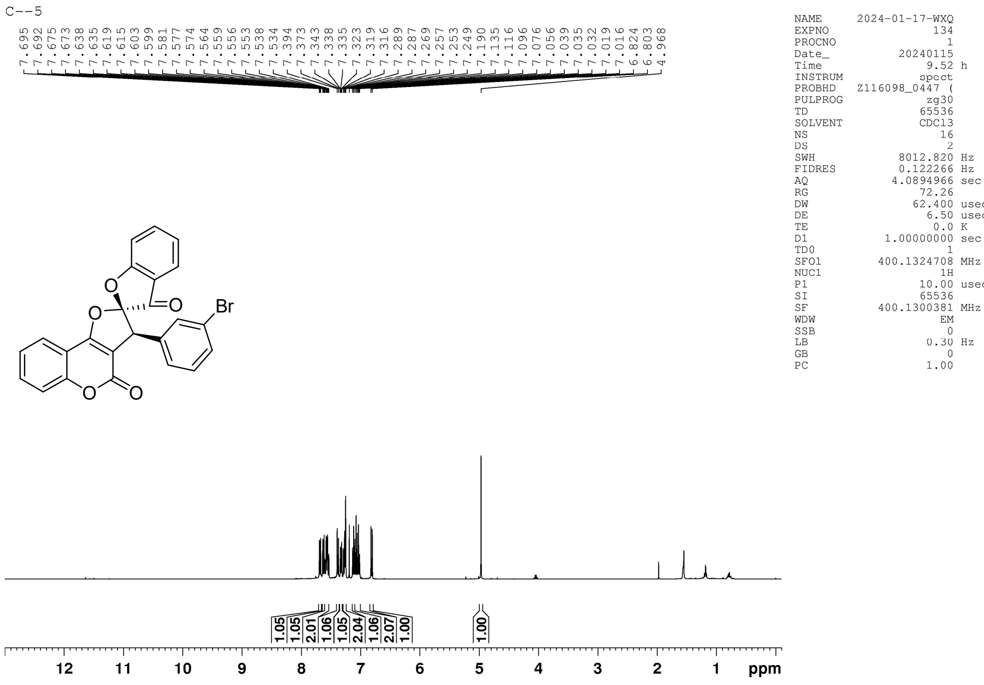


**Figure S53.** $^{1}H$ NMR (400 MHz, $CDCl_3$) spectra of compound **3bc**

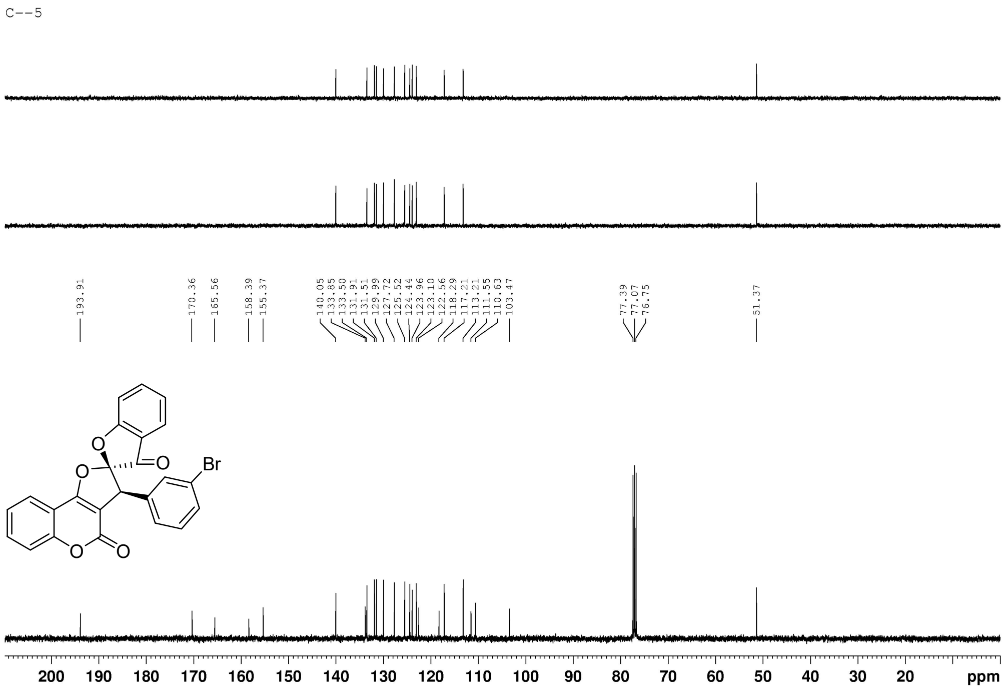


**Figure S54.** $^{13}C$ NMR (100 MHz, $CDCl_3$) spectra of compound **3bc**

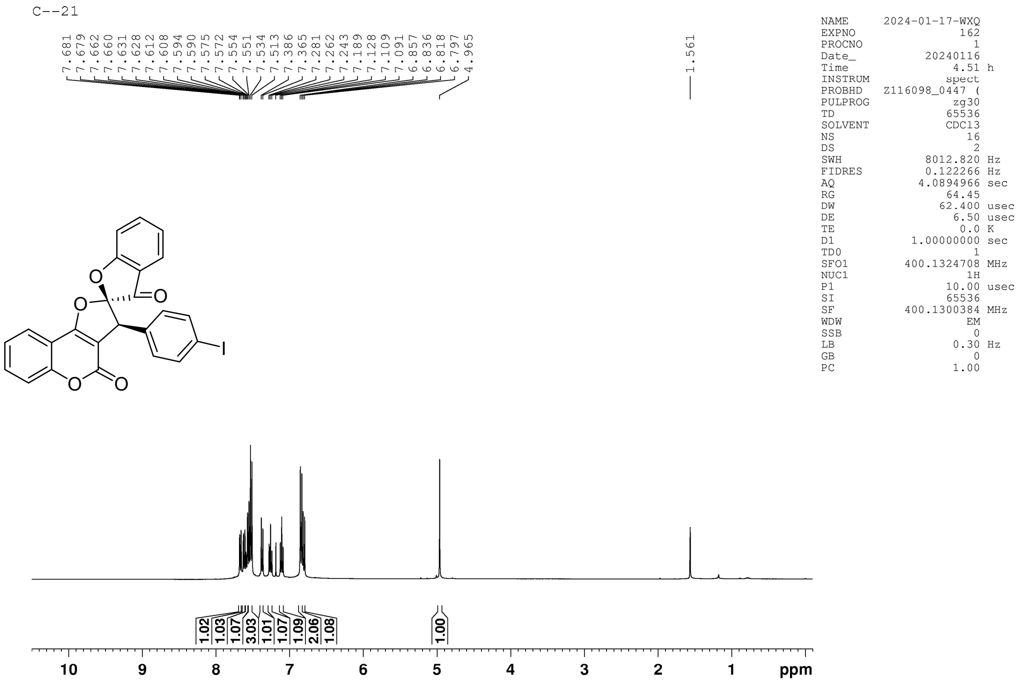

**Figure S55.** $^{1}$H NMR (400 MHz, $CDCl_3$) spectra of compound **3bd**

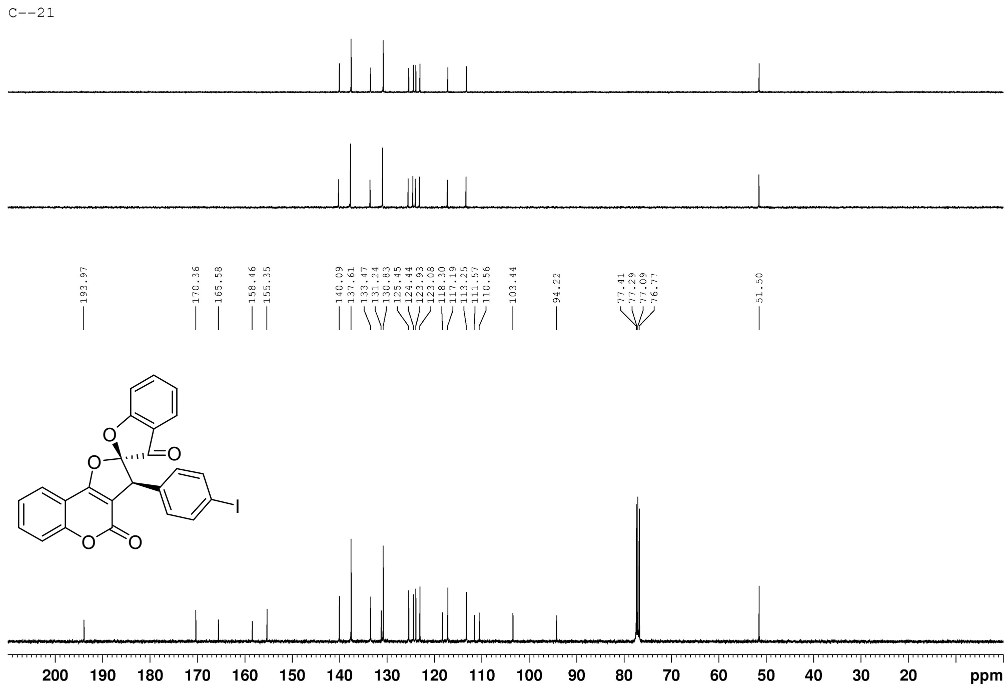

**Figure S56.** $^{13}$C NMR (100 MHz, $CDCl_3$) spectra of compound **3bd**

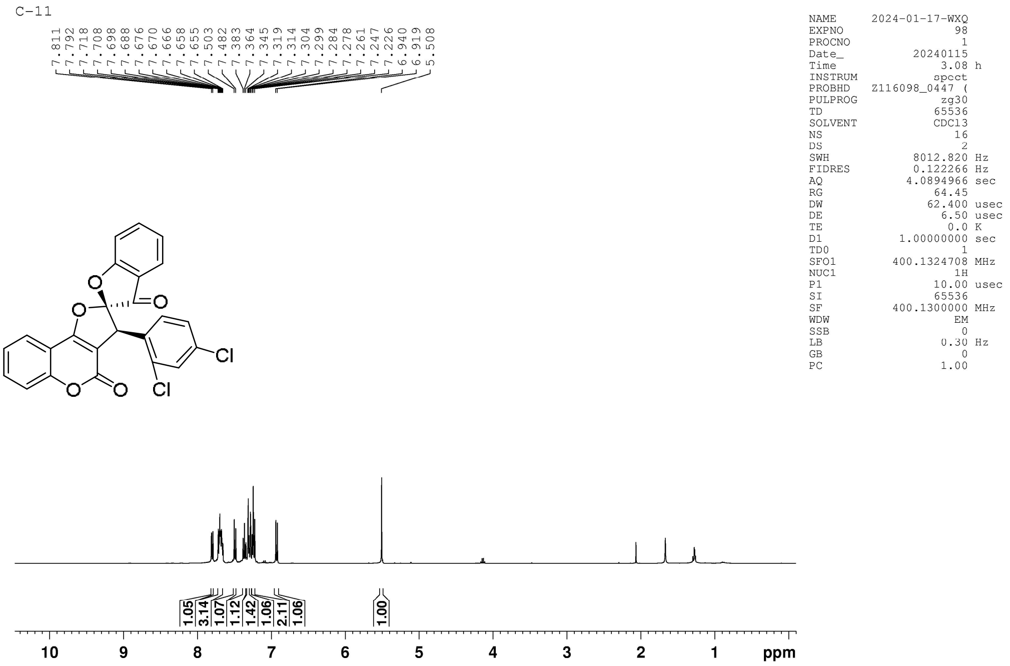


**Figure S57.** $^{1}H$ NMR (400 MHz, $CDCl_3$) spectra of compound **3be**

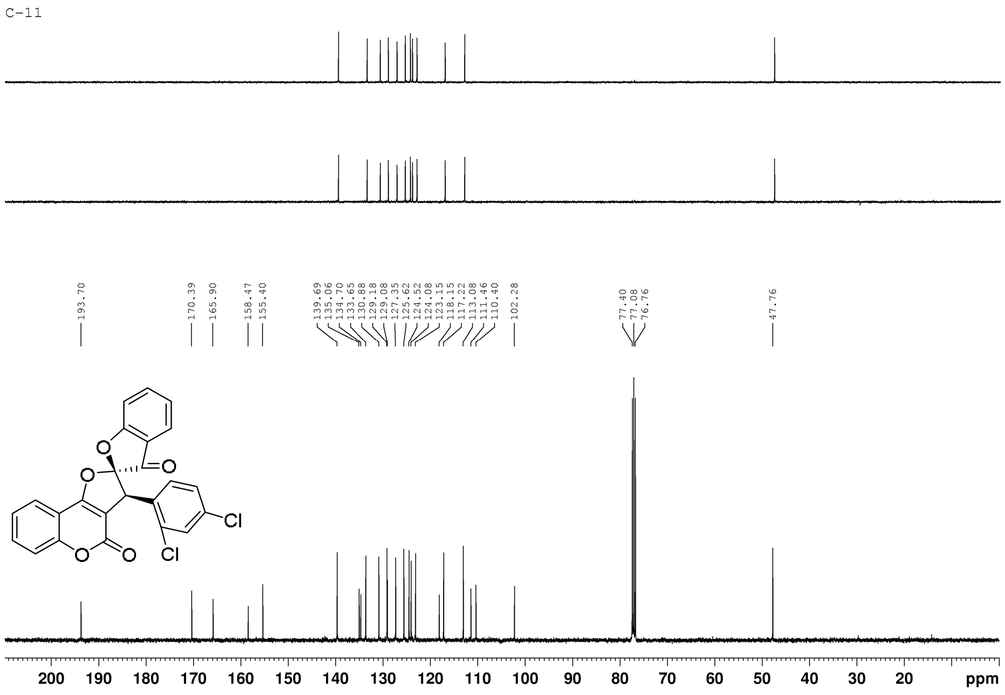


**Figure S58.** $^{13}C$ NMR (100 MHz, $CDCl_3$) spectra of compound **3be**

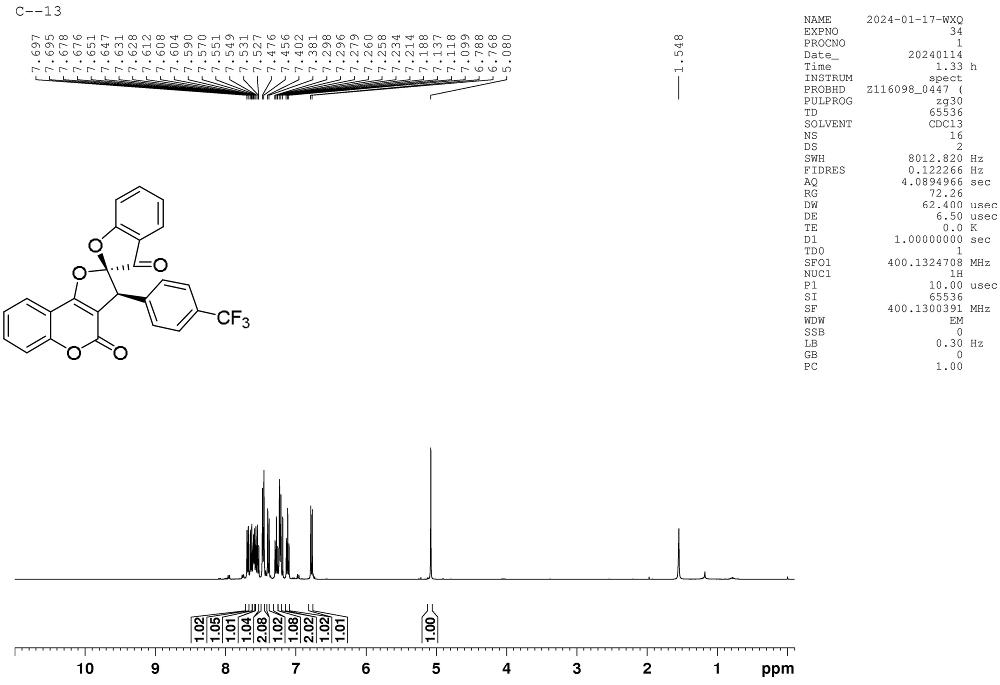


**Figure S59.** $^{1}H$ NMR (400 MHz, $CDCl_3$) spectra of compound **3bf**

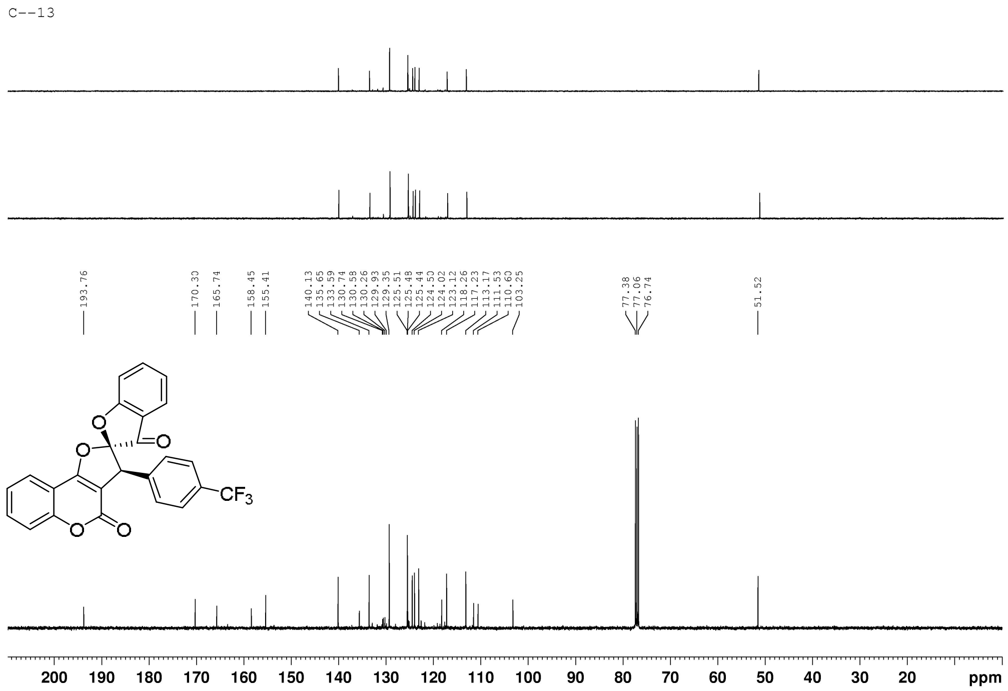


**Figure S60.** $^{13}C$ NMR (100 MHz, $CDCl_3$) spectra of compound **3bf**

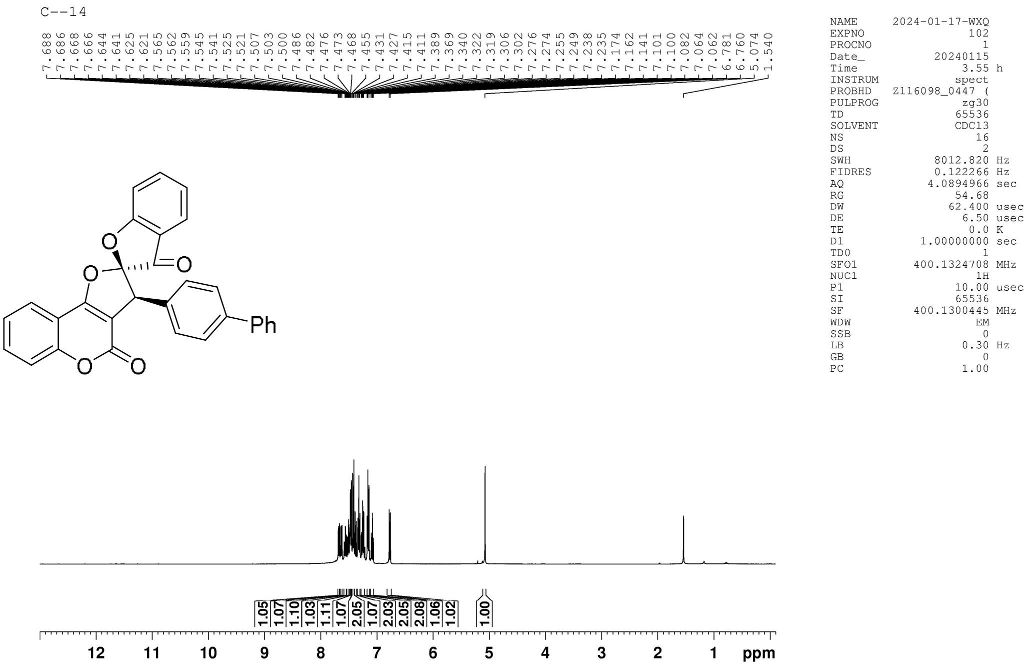


**Figure S61.** $^{1}H$ NMR (400 MHz, $CDCl_3$) spectra of compound **3bg**

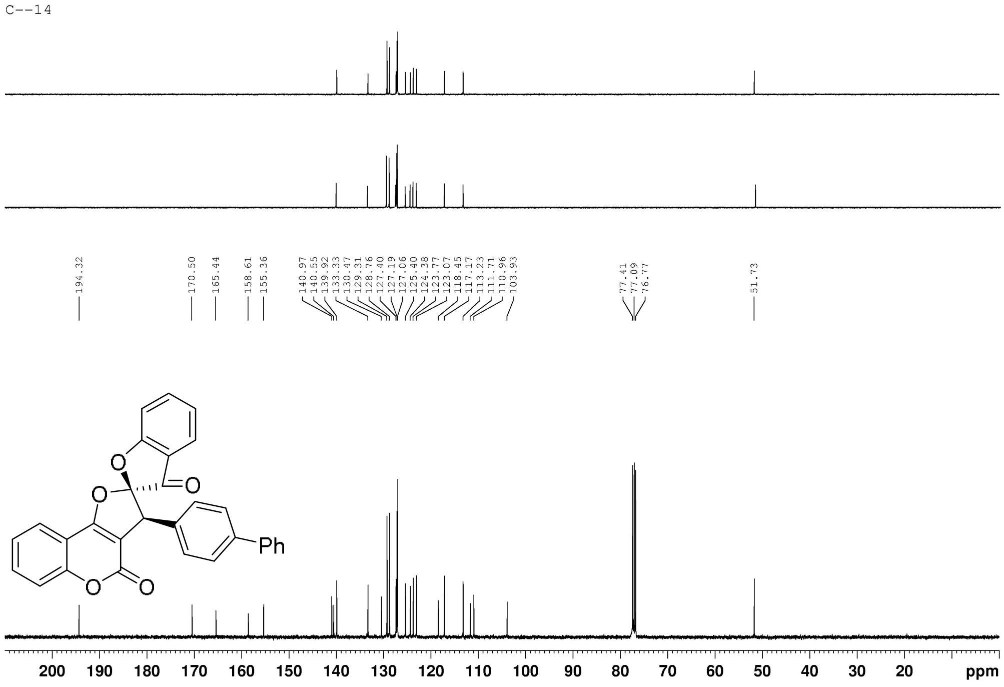


**Figure S62.** $^{13}C$ NMR (100 MHz, $CDCl_3$) spectra of compound **3bg**

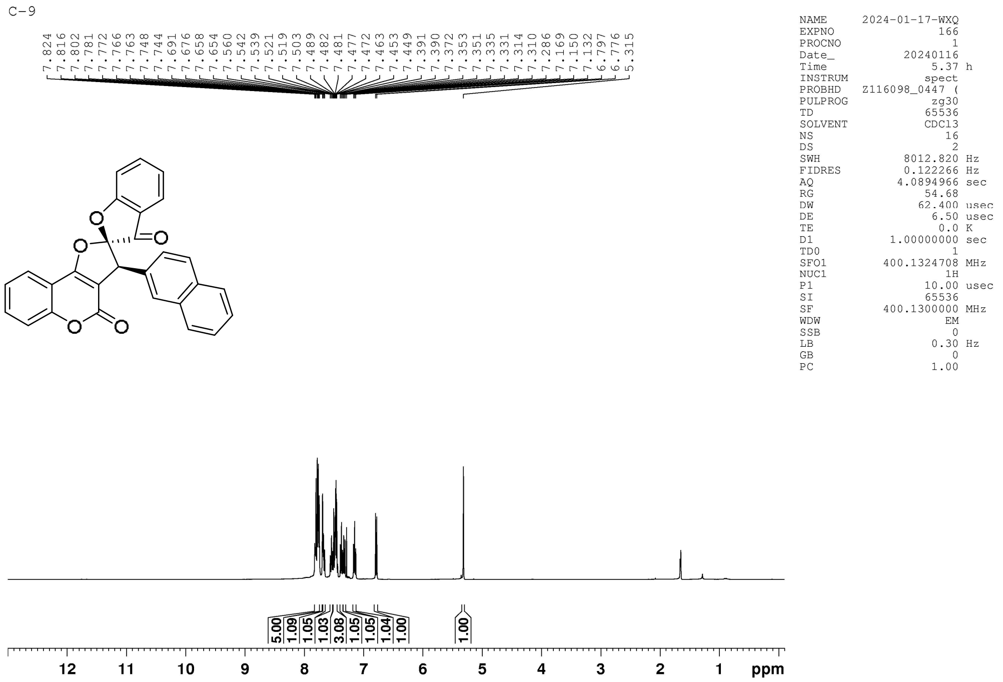


**Figure S63.** $^{1}H$ NMR (400 MHz, $CDCl_3$) spectra of compound **3bh**

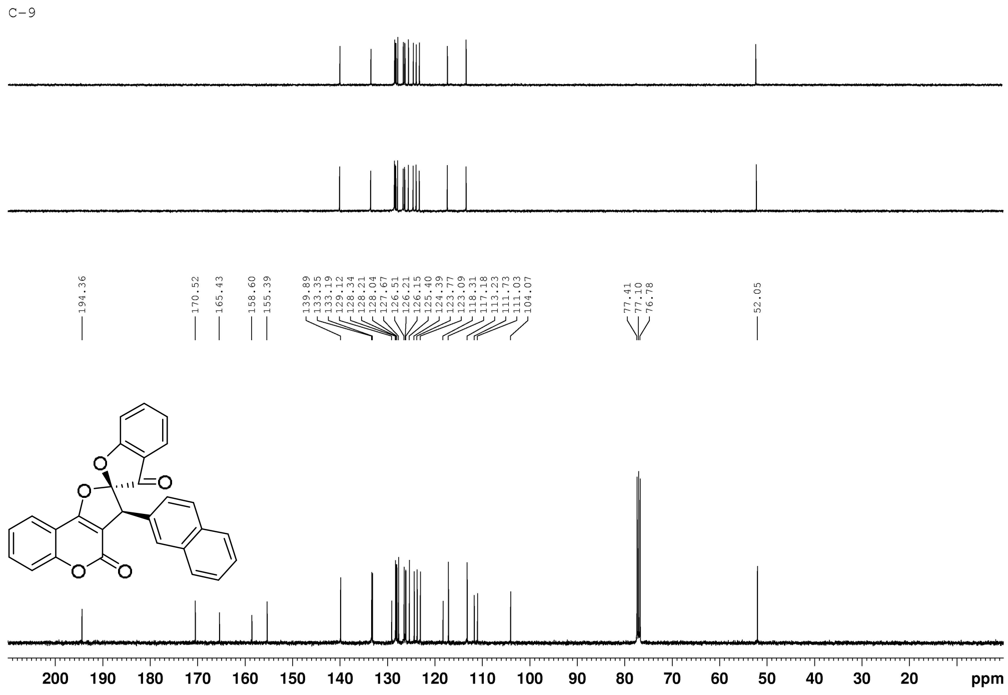


**Figure S64.** $^{13}C$ NMR (100 MHz, $CDCl_3$) spectra of compound **3bh**

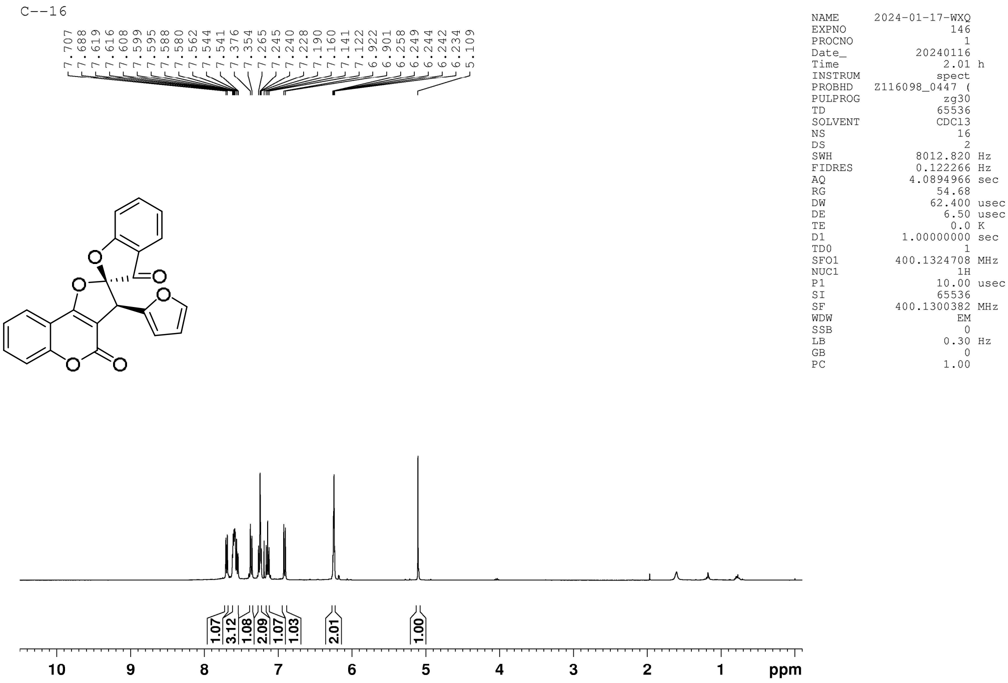


**Figure S65.** $^{1}$H NMR (400 MHz, $CDCl_3$) spectra of compound **3bi**

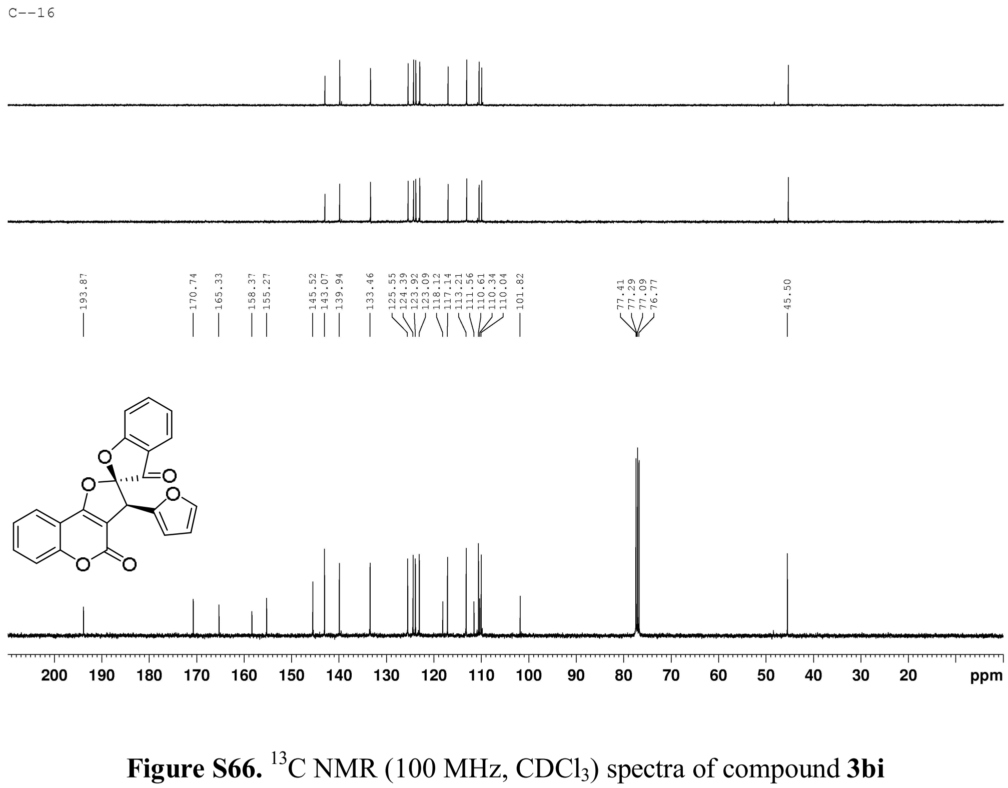


**Figure S66.** $^{13}$C NMR (100 MHz, $CDCl_3$) spectra of compound **3bi**

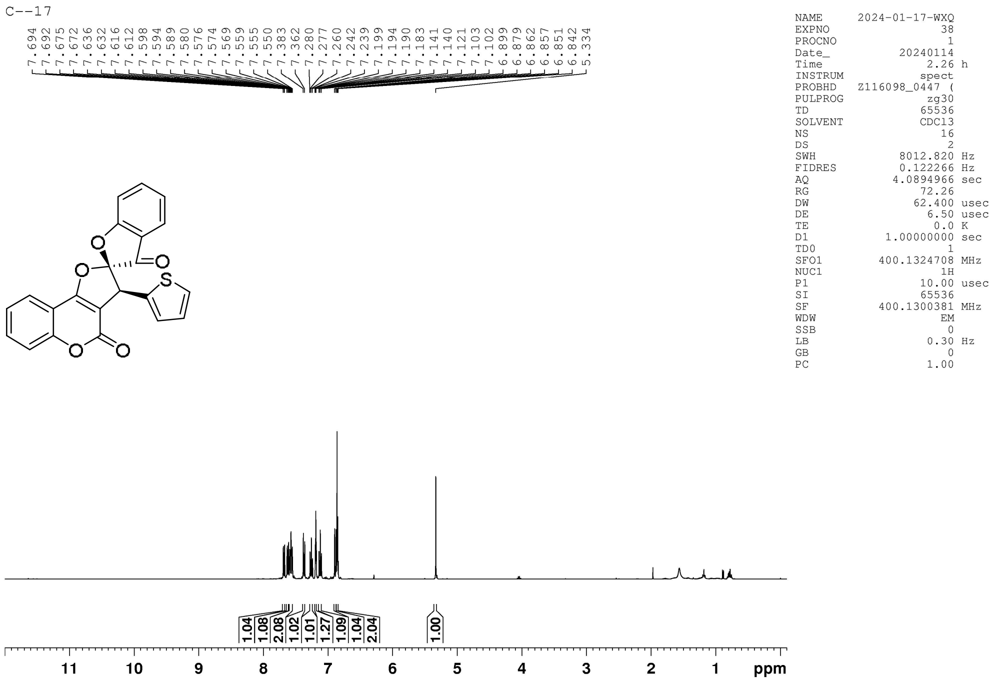

**Figure S67.** $^{1}$H NMR (400 MHz, $CDCl_3$) spectra of compound **3bj**

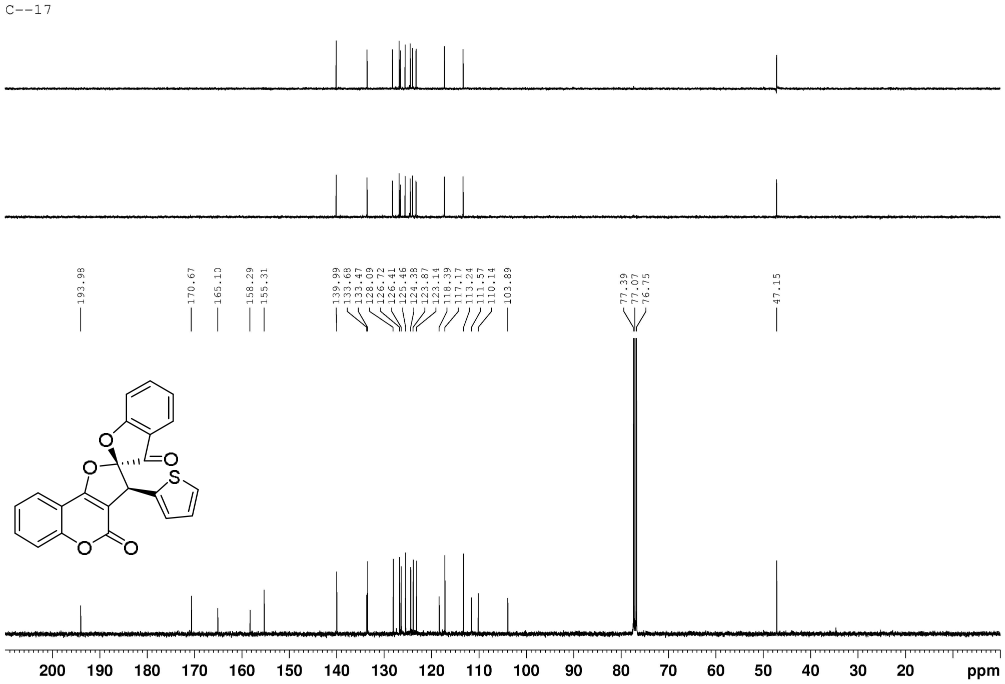

**Figure S68.** $^{13}$C NMR (100 MHz, $CDCl_3$) spectra of compound **3bj**

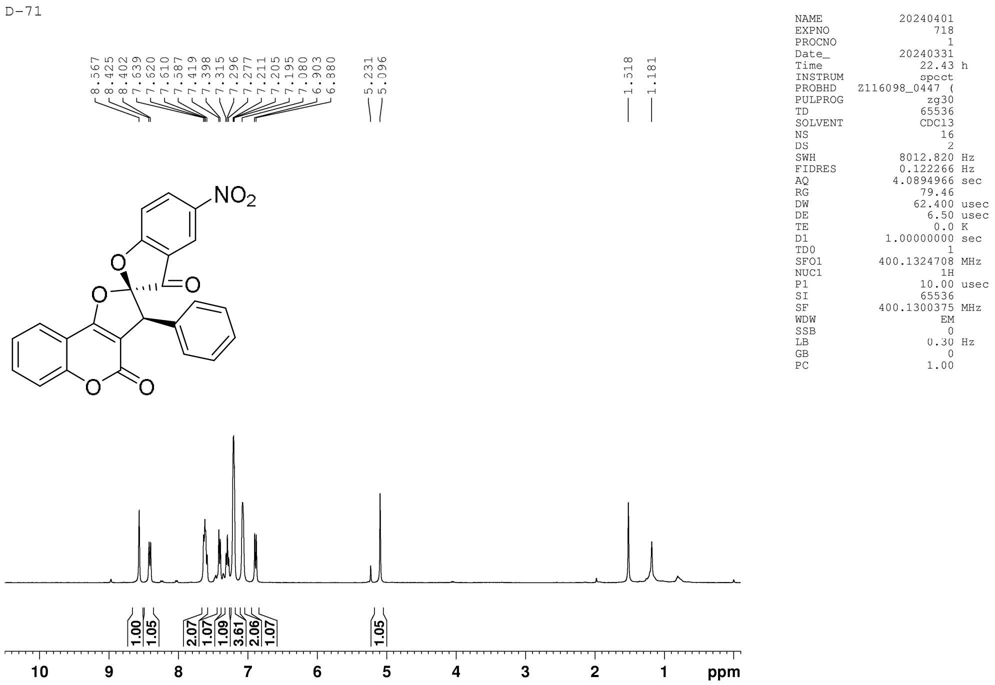

**Figure S69.** $^{1}H$ NMR (400 MHz, $CDCl_3$) spectra of compound **3bk**

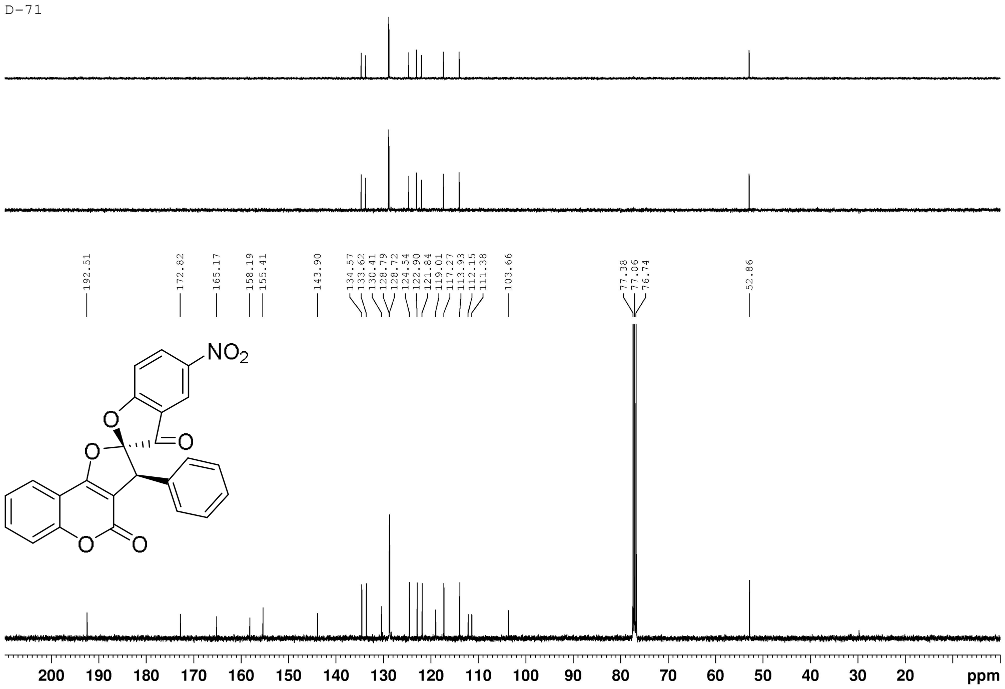

**Figure S70.** $^{13}C$ NMR (100 MHz, $CDCl_3$) spectra of compound **3bk**

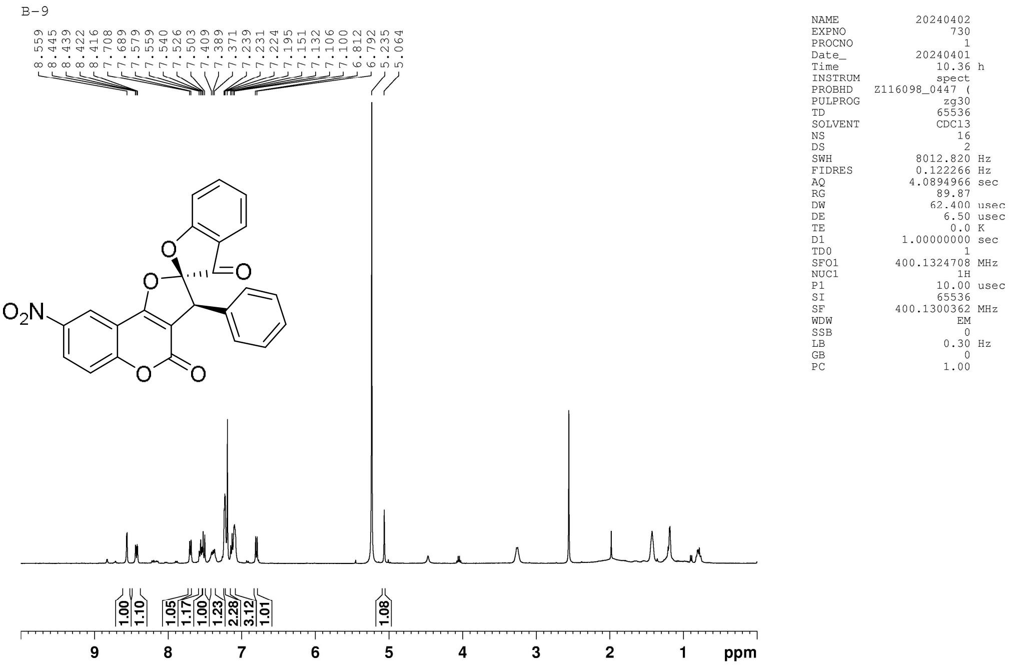


**Figure S71.** $^1$H NMR (400 MHz, $CDCl_3$) spectra of compound **3bl**

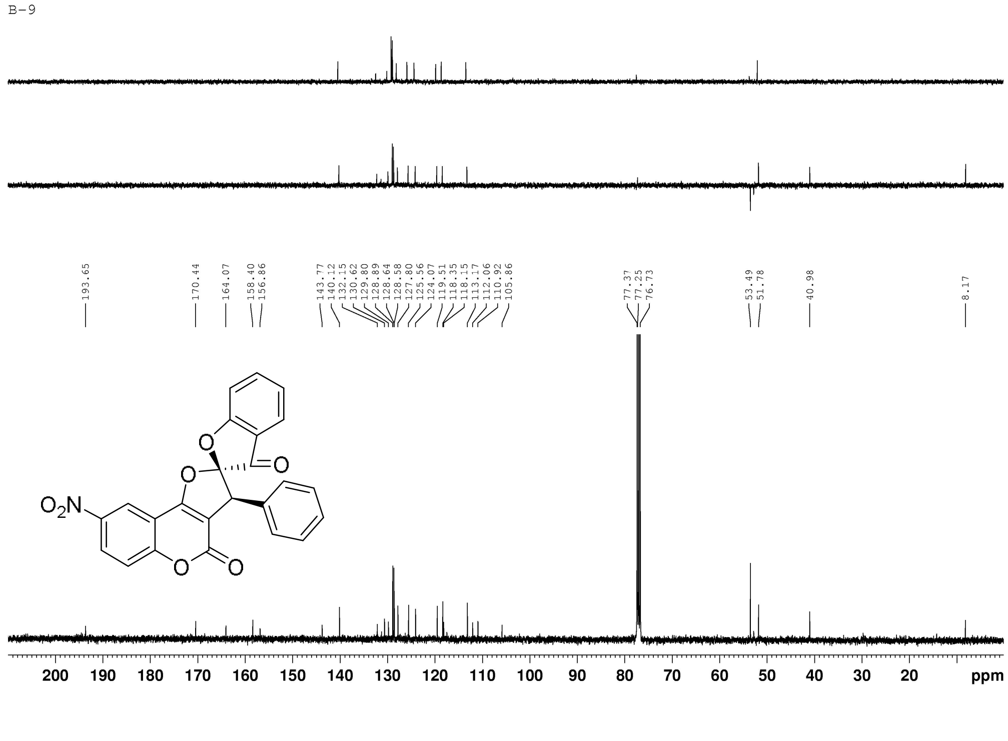


**Figure S72.** $^{13}$C NMR (100 MHz, $CDCl_3$) spectra of compound **3bl**

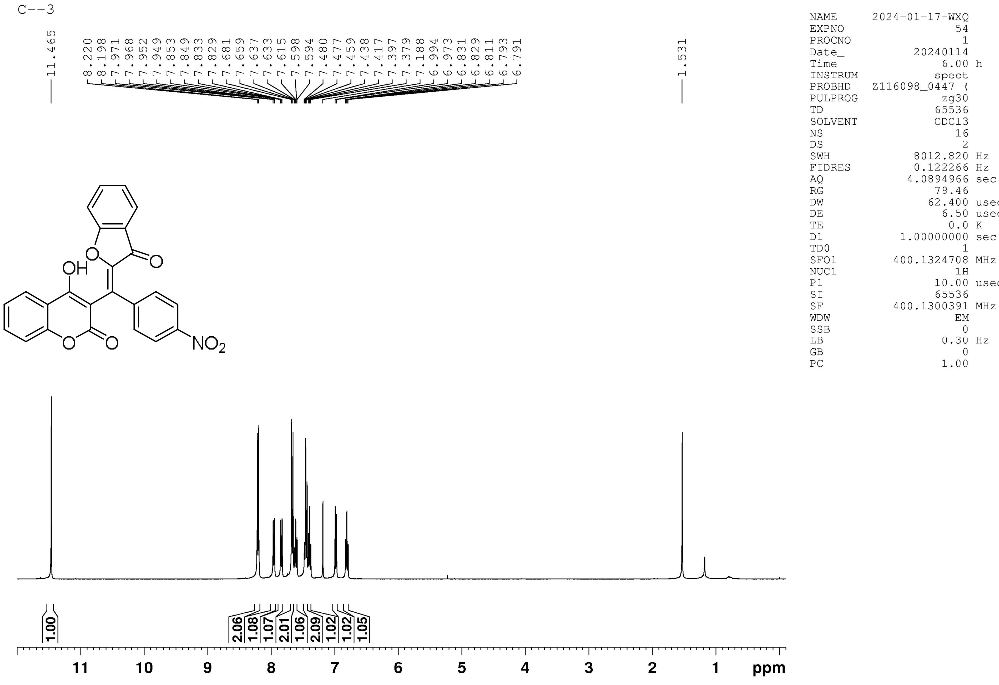

**Figure S73.** $^{1}$H NMR (400 MHz, $CDCl_3$) spectra of compound **4au**

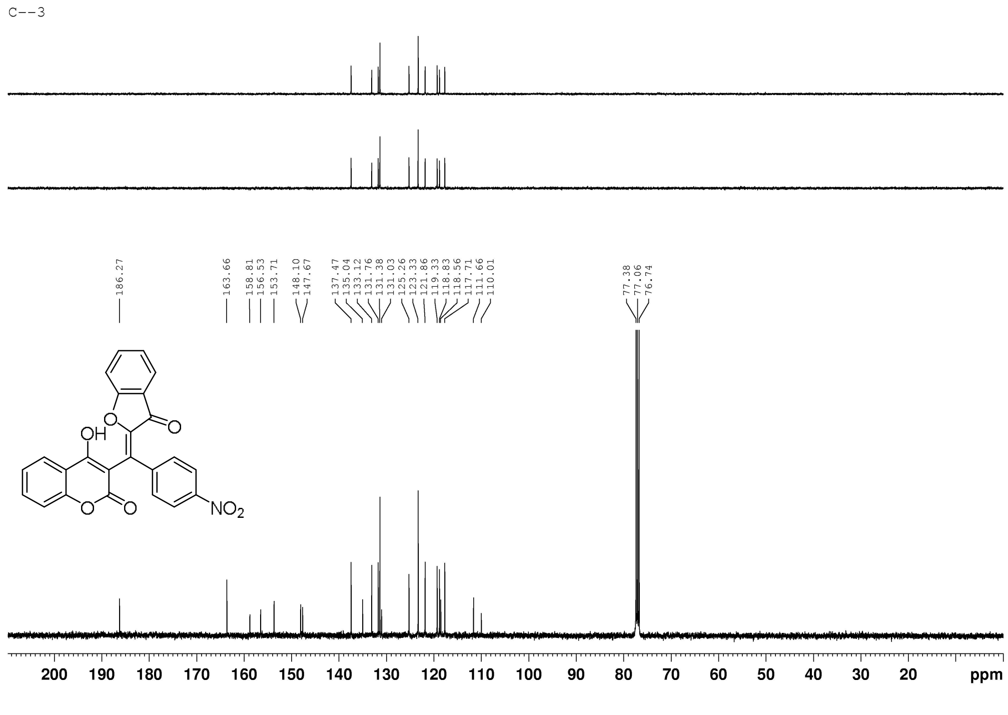

**Figure S74.** $^{13}$C NMR (100 MHz, $CDCl_3$) spectra of compound **4au**

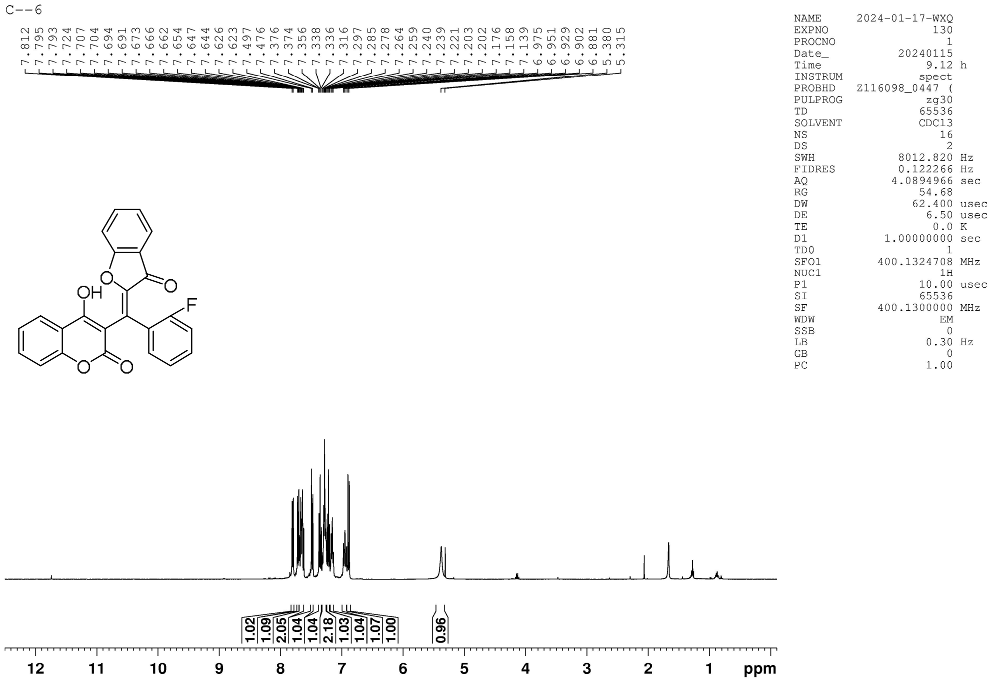


**Figure S75.** $^{1}$H NMR (400 MHz, $CDCl_3$) spectra of compound **4ax**

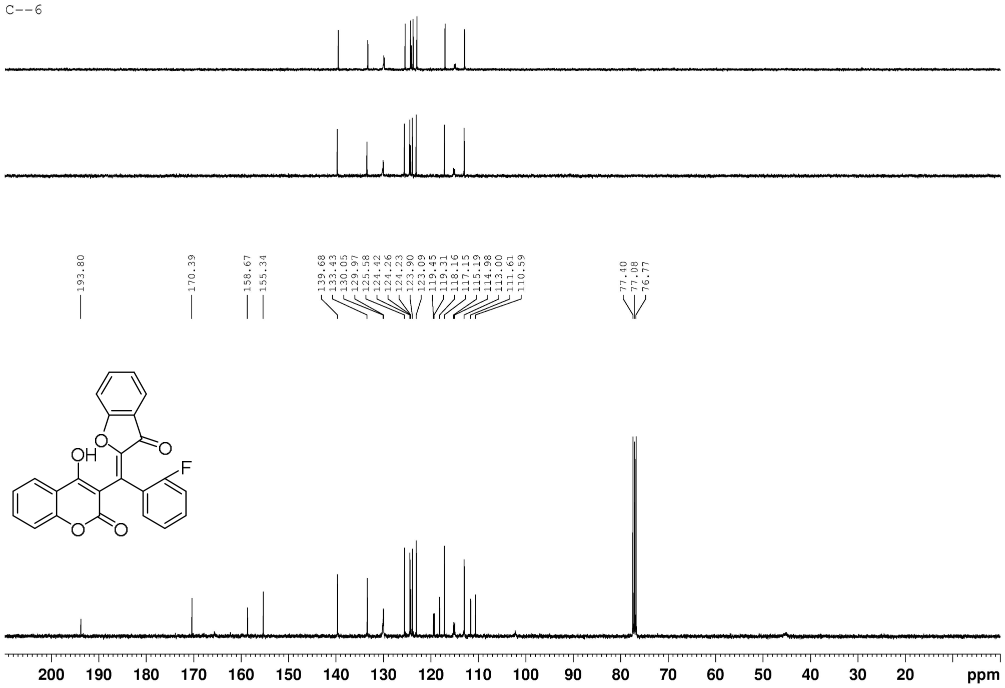


**Figure S76.** $^{13}$C NMR (100 MHz, $CDCl_3$) spectra of compound **4ax**

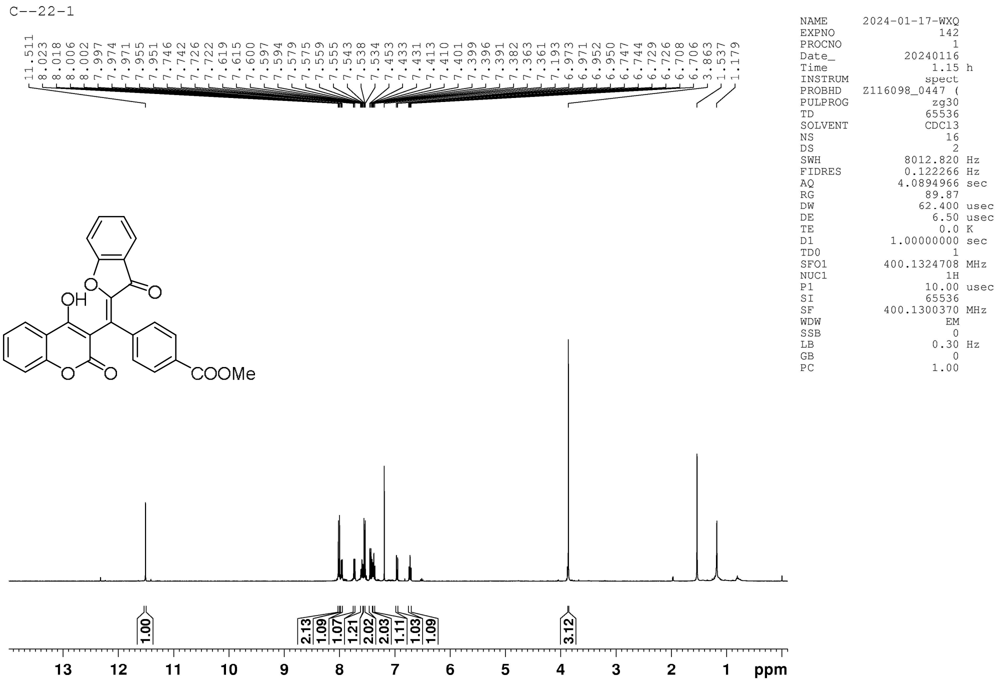

**Figure S77.** $^{1}$H NMR (400 MHz, $CDCl_3$) spectra of compound **4az**

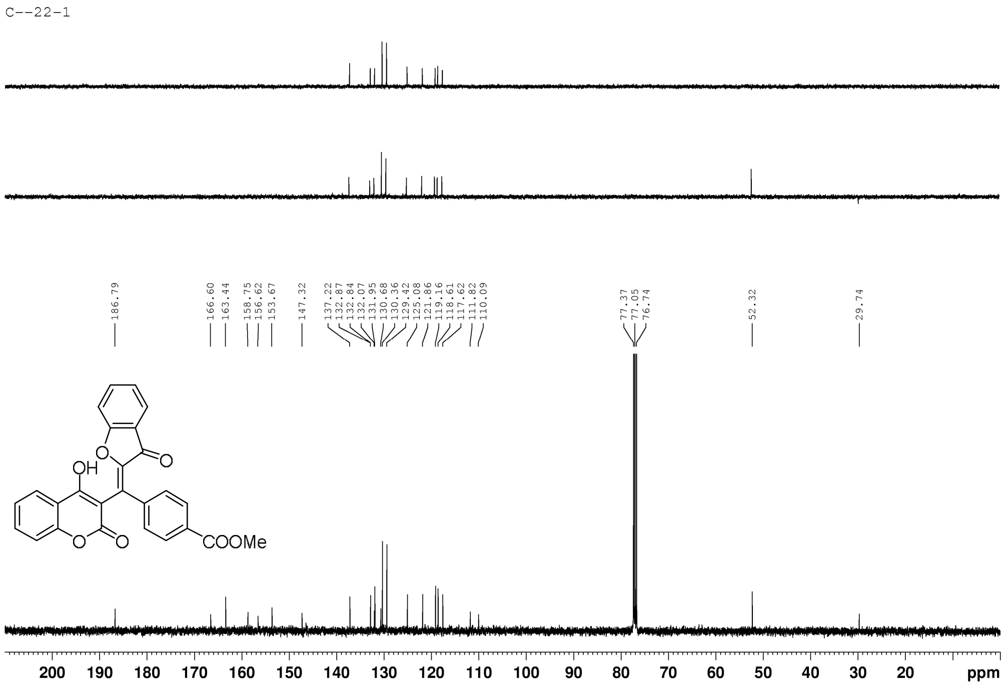

**Figure S78.** $^{13}$C NMR (100 MHz, $CDCl_3$) spectra of compound **4az**

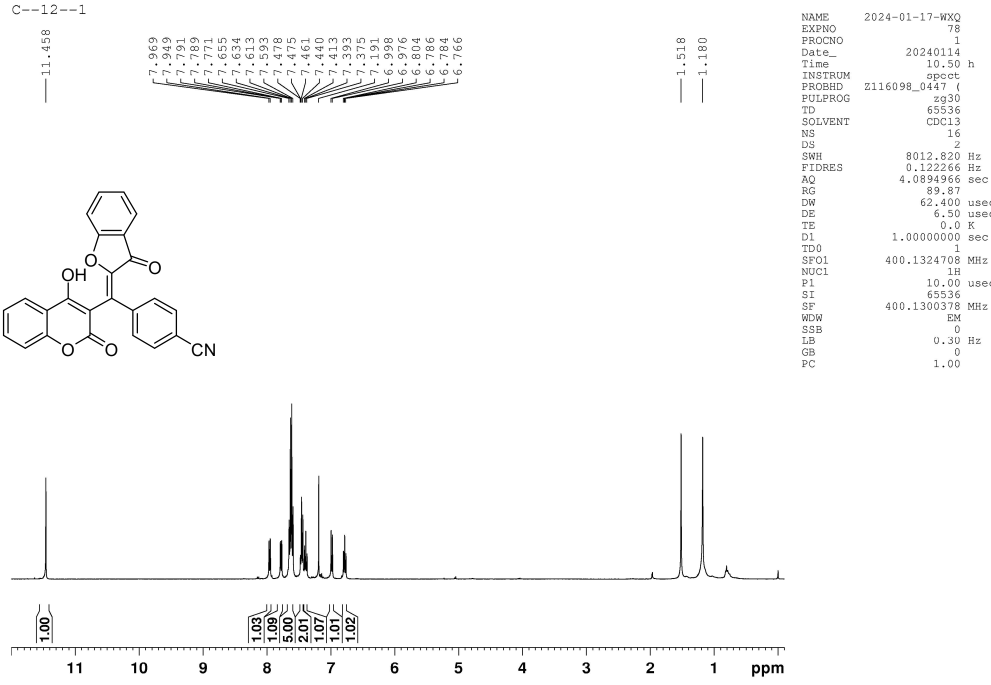


**Figure S79.** $^{1}$H NMR (400 MHz, $CDCl_3$) spectra of compound **4ba**

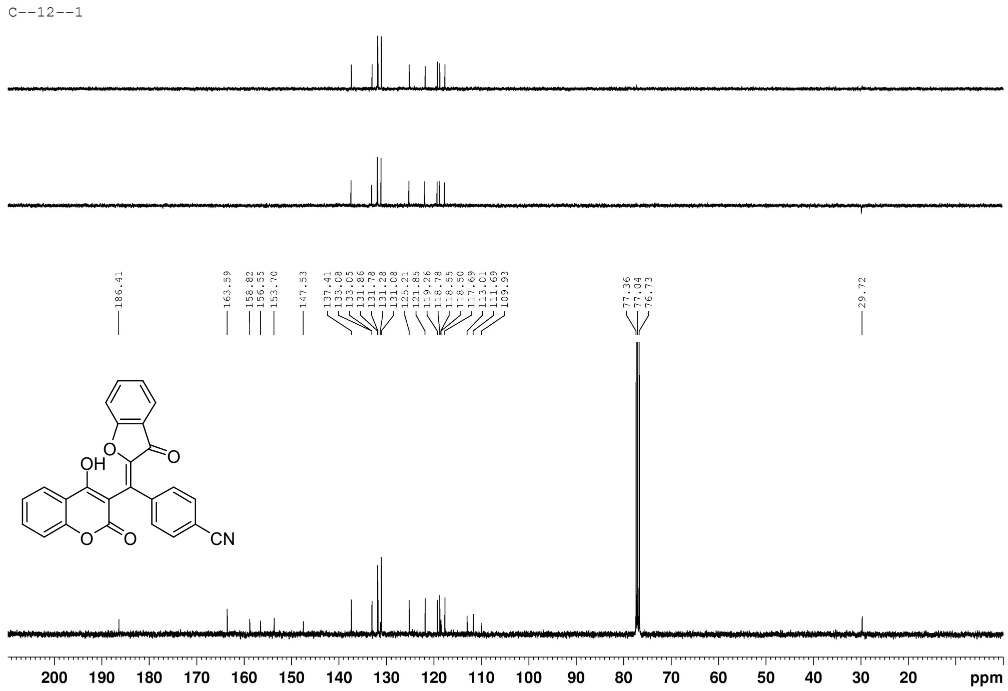


**Figure S80.** $^{13}$C NMR (100 MHz, $CDCl_3$) spectra of compound **4ba**
